# Multifunctional Self-Assembled Peptide Hydrogels for Biomedical Applications

**DOI:** 10.3390/polym15051160

**Published:** 2023-02-25

**Authors:** Mahsa Sedighi, Neha Shrestha, Zahra Mahmoudi, Zahra Khademi, Alireza Ghasempour, Hamideh Dehghan, Seyedeh Fahimeh Talebi, Maryam Toolabi, Véronique Préat, Bozhi Chen, Xindong Guo, Mohammad-Ali Shahbazi

**Affiliations:** 1 Department of Pharmaceutics and Nanotechnology, School of Pharmacy, Birjand University of Medical Sciences, Birjand 9717853076, Iran; 2 Cellular and Molecular Research Center, Birjand University of Medical Sciences, Birjand 9717853076, Iran; 3 Advanced Drug Delivery and Biomaterials, Louvain Drug Research Institute, Université Catholique de Louvain, 1200 Brussels, Belgium; 4 Department of Biomedicine and Translational Research, Research Institute for Bioscience and Biotechnology, Kathmandu P.O. Box 7731, Nepal; 5 Research Center for Molecular Medicine, Hamadan University of Medical Sciences, Hamadan 6517838636, Iran; 6 Department of Pharmaceutical Biotechnology, School of Pharmacy, Mashhad University of Medical Sciences, Mashhad 9177948954, Iran; 7 Student Research Committee, Birjand University of Medical Sciences, Birjand 9717853076, Iran; 8 Department of Biomedical Engineering, University Medical Center Groningen, University of Groningen, Antonius Deusinglaan 1, 9713 AV Groningen, The Netherlands; 9 State Key Laboratory of Organic-Inorganic Composites, Beijing University of Chemical Technology, Beijing 100029, China; 10 Beijing Laboratory of Biomedical Materials, College of Materials Science and Engineering, Beijing University of Chemical Technology, Beijing 100029, China; 11 W.J. Kolff Institute for Biomedical Engineering and Materials Science, University of Groningen, Antonius Deusinglaan 1, 9713 AV Groningen, The Netherlands

**Keywords:** peptide-based hydrogels, biocompatibility, biodegradability, biomedical applications, self-assembly

## Abstract

Self-assembly is a growth mechanism in nature to apply local interactions forming a minimum energy structure. Currently, self-assembled materials are considered for biomedical applications due to their pleasant features, including scalability, versatility, simplicity, and inexpensiveness. Self-assembled peptides can be applied to design and fabricate different structures, such as micelles, hydrogels, and vesicles, by diverse physical interactions between specific building blocks. Among them, bioactivity, biocompatibility, and biodegradability of peptide hydrogels have introduced them as versatile platforms in biomedical applications, such as drug delivery, tissue engineering, biosensing, and treating different diseases. Moreover, peptides are capable of mimicking the microenvironment of natural tissues and responding to internal and external stimuli for triggered drug release. In the current review, the unique characteristics of peptide hydrogels and recent advances in their design, fabrication, as well as chemical, physical, and biological properties are presented. Additionally, recent developments of these biomaterials are discussed with a particular focus on their biomedical applications in targeted drug delivery and gene delivery, stem cell therapy, cancer therapy and immune regulation, bioimaging, and regenerative medicine.

## 1. Introduction

Self-assembling in biological systems has attracted immense attention for creating functional supramolecular structures from individual macromolecules. One of the most essential biomaterials, which exhibits excellent self-assembling behaviors, is peptide hydrogels [1,2,3]. Peptide hydrogels are a class of soft materials that use amino acids and peptides as material building blocks and can capably trap the water or fluids in their molecular structure and change into a nanoscale hydrogel under physiological conditions [4,5,6,7,8]. The molecular interactions for the formation of these systems are primarily non-covalent such as hydrogen bonding, hydrophobic, aromatic π-π stacking, and electrostatic interactions [9,10]. The most routine methods for the preparation of this class of hydrogels are sonication, heating–cooling, and adjusting the pH of the solutions, as well as the addition of a suitable salt to the peptide solutions at high pH [7,11,12,13,14]. In addition, the self-assembled peptide hydrogels can gain stimuli-responsivity (pH, temperature, mechanical, ionic strength, biological fluids), varied sol-gel transition (thixotropic gel), and the potential to entrap drug molecules with different properties through physical or chemical linkage [6,15,16]. These properties are highly dependent on the molecular structure of the primary peptides, such as β-sheets, α-helices, coiled secondary structure, and intermolecular interactions [5,6,17,18]. Peptide hydrogels can be designed into several arrangements of amino acids to exert responsiveness toward different stimuli. Such a trigger allows unique temporal and spatial control over the gelation process, thus widening its applicability [19].

Nowadays, inspired by nature, researchers have designed a type of peptide hydrogel that can form a fibrous hydrogel network similar to extracellular matrix (ECM) components in terms of morphology and size and be used in cell constructs or microtissue in regenerative medicine and cancer research [19,20,21]. From the application point of view, peptide-based hydrogels have immense importance due to their impressive use in biomedicine. Recently, there have been many publications that used peptide hydrogels for different applications related to regenerative medicine, gene delivery, controlled drug delivery, biosensors, tissue engineering, and wound healing due to their low immunogenicity, biocompatible features, ease of synthesis, high water content, desirable structures, and stability in the physiological condition [22,23,24,25,26,27,28,29,30]. Additionally, the unique mechanical properties of peptide hydrogel have led to their use in the treatment of different types of wounds. Peptide hydrogels are involved in the wound healing by preventing bacterial infection, creating a suitable environment for cell proliferation and rapid drug release, and the possibility of gas exchange [10,31].

In the present review, a comprehensive overview of the recent developments in peptide hydrogels and the structures of different self-assembling building blocks are described. Moreover, diverse applications of peptide hydrogel systems such as wound regeneration, targeted gene delivery and drug delivery, cancer therapy and immune regulation, bioimaging, the generation of three-dimensional (3D) peptide hydrogel scaffolds for tissue engineering, and stem cell therapy are explained.

## 2. Self-Assembling Peptides: The Building Blocks and Secondary Structures

Amino acids are the “*building blocks*” of peptides and proteins, and their extensive range generates the possibility of a wide variety of diverse peptide/protein structures with different biomedical applications [32,33,34,35]. For example, more than 3 million sequences/structures can be foreseen for a five amino acid-long peptide. Based on their structure, different amino acids have diverse characteristics and, thus contribute differently to the formation of complex structures (Table 1) [6,18]. Accordingly, hydrophilic amino acids are involved in hydrogen bonding, aromatic amino acids play a crucial role in protein folding, and thiol-containing amino acids, such as cysteine, provide a site for the modification of the peptide structure [6,36]. By altering the number and sequence of the amino acids with various physicochemical properties (electrical charge, size, and polarity), peptide structures can be produced with unique properties [18,34,35,37].

The intra-peptide interactions help the primary amino acid chain to self-assemble and form folds within its structure, creating varied secondary structures such as α-helices, β-sheets, β-turns, and random coils [36]. Such systems can be constructed through a careful design of amino acid sequences with the self-assembly ability to provide a variety of structures such as fibers, micelles, tapes, ribbons, and vesicles [38]. The self-assembly process is driven by non-covalent interactions, and a combination of repulsive and attractive interactions can control this process to achieve a well-defined structure in the tubular, fibrillar, or particulate form [9,39]. In addition to intra-peptide interactions, conjugating amino acids with other molecules, such as alkyl chain (peptide amphiphile) or aromatic groups (allow π-π interactions), also produces self-assembled structures [4,5]. Several molecular interactions are involved during the hydrogel formation, such as hydrogen bonds among the amide bonds, -COOH, and -OH groups. Additionally, hydrophobic interactions among the hydrophobic groups, such as the long alkyl chain and benzene ring, also contribute to the non-covalent bonding. π-π stacking interaction among aromatic groups such as fluorenyl, naphthyl, and phenyl plays a vital role in forming peptide-based hydrogels. Ionic interactions between oppositely charged amino acids are also critical non-covalent interactions exploited during the construction of peptide hydrogels. Electrostatic interaction between glutamic acid (Glu) and lysine (Lys) and also aspartic acid and lys/arginine (Arg) are some examples of effective gelation strategies for the construction of peptide hydrogels [40,41,42]. Overall, several interactions work synergistically during the formation of peptide hydrogels and must be critically evaluated during the design and optimization of such hydrogels.

With advancements in the protein chemistry field, scientists have been able to gain complete control of the peptide self-assembly, thus forming a wide array of structures, from delicate polyhedral cages and rings to 3D crystalline or hydrogel structures [23]. Such a degree of control and extensive prospects has led peptide self-assembly to emerge as a potential biofunctional material with applicability in different fields such as regenerative medicine, gene/drug delivery, bioimaging, and tissue engineering [22,23,43,44,45].

### 2.1. Peptides Building Blocks

β-sheets form by a series of hydrogen bonds between amides and carbonyl groups in the backbone arrangement of multiple peptide strands [6,46]. β-sheets have either parallel (C-termini at one end of the structure) or antiparallel structures (alternate N- and C-termini) [46,47,48]. With the increase in the number of strands, the rigidity and toughness of the resulting sheet increase proportionally. Further, varied hierarchical structures, such as tapes, ribbons, and fibers, can be formed with various sheets [18,46,49]. The ability of peptides to create such various β-sheet structures expands their applicability in the field of drug delivery [50,51,52].

β-hairpins are usually formed when two anti-parallel β-sheets are linked by a β-turn sequence [53,54]. These structures can form high-ordered fibrils and stimuli-responsive hydrogel owing to intramolecular folding and intermolecular assembly [55,56]. Owing to the cytocompatibility and biocompatibility of β-hairpin peptides, they have been extensively utilized for various biomedical applications, such as the delivery of active pharmaceutical molecules (e.g., protein/peptide drugs, cells, poorly soluble drugs, chemotherapeutics, and genetic material) [57,58,59,60]. Some of the peptide hydrogels based on β-hairpins have also demonstrated antibacterial activity [61], and they are also used to culture different cells such as fibroblasts and mesenchymal stem cells (MSCs) [62].

α-helix is a common motif of protein secondary structure comprising 3.6 amino acids per right-handed turn. The stabilization of the helix structure is due to the presence of hydrogen bonding among the carbonyl oxygens (*i*) and amide hydrogen atoms (*i* + 4), van der Waal’s forces, and hydrophobic interactions [9,63]. Coiled coils are basic protein folding patterns and comprise at least two α-helixes [63]. These structures are usually characterized by seven residues (***abcdefg****)*, known as heptad, where ***a*** and ***d*** are hydrophobic residues (spacing of 3.5 residues), and the ***e*** and ***g*** positions are occupied by charged residues. So, this sequence can decrease the number of residues in the helical repeats with a slight left-handed spiral [64]. Filaments formed by coiled coils are usually observed in the cytoskeletons and ECM and in some viral coatings. With inspiration from nature, these structures have also been utilized in developing several biomaterials [63,65]. One of the significant benefits of coiled coils is their flexibility and high level of control on their structure and stability, which is impossible in other secondary structures. Owing to these benefits, controlled and precise nanostructures, such as fiber, tubes, gels, or their combinations, can be obtained [64]. In the case of coiled-coil hydrogel systems, it has been shown that by selecting appropriate residues at positions ***a, d, e*,** and ***g***, the produced hydrogels can be responsive to external stimuli, such as temperature, pH, and ionic strength [66,67]. Coiled-coil self-assembled peptides are an emerging and exciting area of research with demonstrated applicability in different fields, such as bioconjugation, drug delivery, and immune therapies [46,64,65].

### 2.2. Self-Assembling Peptides

To form different nanostructures, the self-assembly technique is the primary method that requires special peptide building blocks (short amino acid sequences or repeated amino acid sequences) with the capability to self-assemble. According to applied self-assembling building blocks, peptide nanostructures exhibit distinctive physical, chemical, and biological characteristics primarily dependent on their size, morphology, and surface functional groups [68,69]. Here, the self-assembling building blocks are described by which the design and fabrication of various nanostructures are possible.

#### 2.2.1. Dipeptides

Dipeptides with the interactions of only two amino acids are the simplest self-assembling building blocks in peptide nanostructures. For example, the β-amyloid peptide in Alzheimer’s disease possesses the core recognition motif based on the diphenylalanine (di-Phe) peptide [70]. Several reports suggest that dipeptides can be self-assembled into different highly ordered nanostructures [70,71,72,73,74]. For instance, the di-Phe motif of Alzheimer’s β-amyloid peptide could self-assemble in stiff and discrete nanotubes, and then discrete nanowires could be produced via the reduction of silver (Ag) within the self-assembled nanotubes and enzymatic degradation of dipeptide-based backbone [70]. In another study, the self-assembly of D-Phe-D-Phe molecules led to generate porous nanotubes with the capability to form unique peptide-nanotube platinum-nanoparticle (NP) composites [71]. These peptide nanotubes were also attached to gold electrodes to improve their performance. It was demonstrated that the fabricated composite electrodes provided a direct response to the NADH and hydrogen peroxide at a specific potential, and it could be applied as a glucose biosensor by measurement of produced hydrogen peroxide during the enzymatic reaction of glucose oxidase and glucose. Furthermore, this biosensor was evaluated for detecting ethanol using NAD^+^ and ethanol dehydrogenase [73].

Self-assembled hydrogels were also reported with significant mechanical rigidity produced by the Fmoc–di-Phe peptide. The designed hydrogel had excellent stability under extreme conditions and was suggested for different applications such as tissue engineering and regenerative medicine [74]. N-terminal modifications of di-Phe were also produced, including tert-butoxycarbonyl (Boc)-Phe-Phe-COOH, N-Carbobenzoxy(Z)-Phe-Phe-COOH and Fmoc-Phe-Phe-COOH by which other tubular structures could be achieved [75]. Diphenylglycine is a very simple aromatic dipeptide that can self-assemble and produce stable spherical nanostructures. It was revealed that nanospheres could also be prepared by introducing a thiol group into the di-Phe [76]. Instead of α-amino acids, β-amino acids are applied in dipeptide-based self-assembly to provide remarkable structural diversity. It was previously shown that the hydrogels formed by β-amino acids had a prolonged bioavailability compared to α-amino acids [77,78].

#### 2.2.2. Peptide Amphiphiles with an Alkyl Group

Peptide amphiphile (PA) is a category of self-assembling structures composed of two distinct regions: hydrophobic alkyl chain and hydrophilic short peptide sequence [9,18,79,80]. The PAs have the potential to assemble into cylindrical or fibril geometries with a hydrophobic core and hydrophilic peptide presented on the surface [6,9]. The formation of such structures allows its administration in the encapsulation of hydrophobic and hydrophilic drugs. Furthermore, this feature also permits designing various bioactive moieties on the surface of the nanostructures. There are four main regions in a PA [44,81]. The first region as a hydrophobic part can be designed using alkyl chains with varied lengths, multiple alkyl chains, or other hydrophobic components. The rigidity of the nanorods formed from these structures is influenced by the presence of phospholipids. Adding a low proportion of phospholipids results in increased mechanical strength, and high ratios subsequently lead to disruption of the hydrogen bonding network in β-sheet conformation [82]. The second region, adjacent to the alkyl chains, is composed of hydrophobic amino acids with a high probability of forming β-sheet conformation. This region is very crucial for the formation of self-assembled nanostructures. Further, studies have demonstrated the influence of this region on the mechanical properties of the gels and other nanostructures [44,83]. The third region is composed of charged amino acids. By suitable selection of amino acids, the solubility of the PA and its ability to respond to the salt composition and pH of the solution can be governed. Such stimuli responsivity in a system allows for developing advanced systems, such as in situ gelling 3D structures. In the last region, bioactive peptide epitopes impart biological functionalities, such as cellular adhesion and active targeting. The placement of such bioactive moiety at the end of the peptide chain allows tailored bioactivity without altering the desired cylindrical/fibrillar structure [5]. Further, short spaces amino acids comprising one or two glycine molecules have also been used to separate peptide epitope from the charged groups, which allow better access to the epitope [84].

#### 2.2.3. Surfactant-like Peptides

A surfactant is defined as a molecule with the ability to significantly reduce the surface tension of water, causing its solubility in both aquatic and organic solvents in very low concentrations. De novo designed surfactant-like peptides (SLP) are acting as surfactants with some hydrophobic residues as the tail and hydrophilic charged residues as the head, and their amphiphilic structure results in their solubility [29,85]. Self-assembly of SLPs is critically dependent on amphiphilicity to regulate the process of hydrophobic attractions of peptides. In a study, the self-assembly and cellular effects of isomeric SLP-based nanostructures were investigated composed of Phe and Arg. It was demonstrated that the modulation of different cellular responses was mediated by the amphipathic design of SLPs [86]. Stimuli-responsive nanostructures were also prepared by SLPs. Peptide Arg_3_-Leu_12_ revealed a pH-dependent self-assembly feature and could form peptide nanotubes at pH 9 and below. At higher pHs, vesicular aggregates were produced by these peptides [87]. In recent years, Gemini surfactant-like peptides have received much attention due to their advantages in the self-assembly process to fabricate nanostructures. In a study, a simple method was reported to design Gemini-like peptides based on natural amino acids with the linear sequence of Ac-AAAAAAPKKPAAAAAA-NH_2_ (APK). This peptide showed great potential to self-assemble and encapsulate hydrophobic drugs such as paclitaxel (PTX), doxorubicin (DOX), etomidate, and propofol, and the designed formulations presented antitumor, antibacterial, or anesthetic efficiency [88].

#### 2.2.4. Bolaamphiphilic Peptides

SLPs and bolaamphiphiles differ in the number of hydrophilic heads of the self-assembly building block. There is only one hydrophilic head in the SLPs, while two are in the bolaamphiphiles connected by a hydrophobic section [89]. This kind of structure with two heads leads to unique characteristics and a complex assembly process. Different head groups can be applied at either end of the hydrophobic section to create asymmetric bolas [90]. There are different sequences of bolaamphiphiles peptides that are related to amyloid-like aggregation. For instance, in Lys-Ala_4_-Lys, Lys-Ala_6_-Lys, and Arg-Ala_6_-Arg bolaamphiphilic peptides, Lys and Arg have a hydrophilic property connected by hydrophobic Ala amino acids, and they can self-assemble to a fibrous structure [91]. By changing the charge of amino acids at different pHs, these self-assembled structures can be applied as pH-responsive materials [91]. Recently, the self-assembly and antimicrobial effect of two bolaamphiphilic peptides, Arg-Ala_6_-Arg and Arg-Ala_9_-Arg, were studied. The high hydrophobicity of the Ala_9_ section caused it to self-assemble into ordered nanofibers, while Arg-Ala_6_-Arg could not self-assemble in water because of its high solubility. It was also shown that the cytocompatibility of Arg-Ala_6_-Arg was higher than Arg-Ala_9_-Arg. Arg-Ala_6_-Arg demonstrated antibacterial activity against *Pseudomonas aeruginosa*, but Arg-Ala_9_-Arg had little antimicrobial activity [92].

#### 2.2.5. Cyclic Peptides

Peptide cyclization usually imparts the peptide structures with more rigidity and stability. Compared to linear peptide counterparts, the new generation of cyclic peptides is demonstrated to be less prone to proteolysis and has also shown higher binding affinity and better entropy in receptor binding [93]. Cyclic peptides have been shown to form self-assembled nanotubes by stacking the peptides, which are stabilized by hydrogen bonding [23]. The cyclic peptides must have a flat conformation with the side chains protruding outwards and the amide and carbonyl groups in the backbone oriented perpendicular to the ring [94]. Generally, the cyclic peptides are composed of alternating D,L-α-amino acids, β-amino acids, alternating α,β-amino acids, alternating α,γ-amino acids, and δ-amino acids [23,94,95,96].

Ghadiri et al. were the first to report a well-characterized peptide open-ended nanotube with a uniform shape and internal diameter comprised of octapeptide cyclo-[(L-Gln-D-Ala-L-Glu-D-Ala) 2-] [97]. Nanotubes prepared from self-assembled cyclic peptides have a high degree of control over the nanotube diameter by choosing the number of amino acids in the cyclic polypeptide. They can also alter the characteristics of the nanotube surface by selecting particular amino acids or modifying their side chains [98]. Such control allows the design and development of nanotubes with specific properties aimed at definite applications, from biosensors to drug carriers and from electronic devices to transmembrane transporters for ions, small molecules, or hydrophilic drugs [98,99,100]. Cyclic peptide scaffolds have also been studied as high-density lipoprotein complexes to remove cholesterol from blood circulation [101].

#### 2.2.6. Fluorenylmethoxycarbonyl Peptides

Self-assembling characteristics can also be imparted on peptides by chemically modifying the N-terminus with aromatic groups, such as fluorenylmehoxycarbonyl (Fmoc). The addition of the aromatic group assists in forming stable self-supporting β-sheets hydrogels with rheological behavior like solid-gel [46]. The gel formation is aided the π-π stacking of the aromatic groups, which results in β-sheet formation and fibrillation [46]. The peptides are present in anti-parallel arrangements of β-sheets, where Fmoc moieties act as a zipper to bring two adjacent sheets together, forming a cylindrical architecture [102]. The use of aromatic groups allows the formation of self-assembled structures with shortened peptide sequences. The gel formation triggers by changes in pH, solvent polarity, and enzymatic actions, which also govern the morphology of the resultant hydrogels. The nature of building blocks influences the morphology of the Fmoc-based nanostructure. Thus, many nanostructures can be designed from Fmoc peptides [103]. The use of Fmoc-modified short peptides was demonstrated by Ulijn and co-workers, with Fmoc- di-Phe and Fmoc-arginine–glycine–aspartic acid (Fmoc-RGD), which formed hydrogels at neutral pH [104]. Fmoc- di-Phe and Fmoc-RGD hydrogels demonstrated the ability to encapsulate and induce the proliferation of chondrocytes and dermal fibroblasts, respectively [104,105]. Furthermore, the combination of Fmoc- di-Phe and Fmoc-RGD has been shown to form dense hydrogel scaffolds with dermal fibroblasts that closely mimic ECM. The presence of Fmoc moiety is a crucial part of forming self-assembled structures [5].

By altering the number and sequence of amino acids linked to the Fmoc group, hydrogels with different characteristics can be obtained. For example, when Fmoc- di-Phe was gelled in the polysaccharide solution of konjac glucomannan (KGM), a highly stable hydrogel was formed. Such hydrogels have great potential in colonic delivery of drugs as KGM degraded in the presence of β-glycosidase, mainly found in the colon [106,107]. In a study by Chu et al., photo-responsive supramolecular hydrogels were prepared using Fmoc-RGDS [108]. The hydrogel was stabilized by host-guest interaction. Fmoc-RGDS were used as the peptide backbone owing to their ability to form hydrogel and also serve as a cell adhesion motif. Cyclodextrin vesicles (CDVs) are used as non-covalent cross-linkers, and arylazopyrazole (AAP) is used as a water-soluble photo switch guest, which is linked to Fmoc-RGDS (Fmoc-RGDS-AAP). A supramolecular reversible photo-responsive hydrogel was formed by mixing Fmoc-RGDS, Fmoc-RGDS-AAP, and CDV in optimal fractions. The supramolecular hydrogel was not only able to serve as a reservoir but also demonstrated step-wise release of three different payloads at different release rates: FITC-Isomer I, FITC-Dextran4000, and Nitrobenzoxadiazolyl -cholesterol (NBD-Cholesterol) [108].

#### 2.2.7. Peptide-like Structures

Two major chemical methods are applied for peptide production including solid phase peptide synthesis (SPPS) and solution phase synthesis (SPS) [109]. In SPS technique, single amino acids are coupled in solution and long peptides are subsequently synthesized via the fragment condensation method. In principle, short peptide sequences are first synthesized, then coupled together to prepare a long-desired peptide [110,111]. In the SPPS technique, resin is applied as a support for anchoring the growing peptide. To synthesize a peptide, first, an amino acid with temporary protecting groups and the α-amino group is attached to the resin via its C-terminus and then the protection group is removed. This process is repeated to complete the peptide sequence [112]. Microwave-assisted SPPS is developed to improve SPPS method for synthesizing long peptides [113]. In chemical synthesis, the number of coupling steps causes a decrease in the purity of the final products. To solve its limitation, novel protecting groups and new techniques are introduced to enhance the quality and quantity of peptide products [109]. Additionally, the cleaved deprotected peptide in the chemical synthesis forms insoluble resistant clumps upon dessication [114]. In this case, the optimization of peptide sequences for their solubility and functionality can lead to create branched amphiphilic peptides as reported by Natarajan et al. with liposome-like behavior in self-assembly process [115]. Furthermore, functional branched polyaminoacids can be built up through a facile way with tunable physicochemical and biological characteristics [116]. Branched polyaminoacids were constructed by the ring opening reaction of polysuccinimide with L-Arg or Gly at controlled pH condition. It was demonstrated that the optimization of the pH influences the physicochemical properties of copolymers.

De novo peptides have been explored to self-assemble into supramolecular nanostructures and it can show how differences in peptide design can translate to relatively small changes in the final structure and self-assembled topologies [117,118]. In a study, bioinspired de novo design was performed to obtain a coiled-coil-forming helical heptapeptide serving as the basic module in some biological recognition processes. By considering catalytic residues into the heptapeptides, a metal-free phosphatase mimic was created through the hierarchical self-assembly into supramolecular assemblies [119].

## 3. Peptide Hydrogels: Stimuli-Responsive Properties

**Figure 1:**
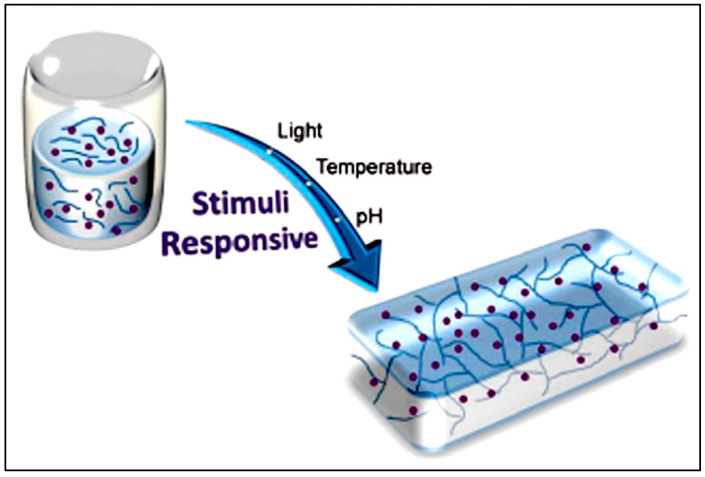
Schematic illustration of a stimuli-responsive hydrogel which shows a change in properties against internal or external stimuli. Reprinted with permission from Ref. [120]; Copyright 2013, De Gruyter.

Peptide hydrogels can be designed into several arrangements of the varied amino acid to exert responsiveness towards different stimuli such as pH, solvent, enzyme, and light (as depicted in Figure 1). The use of such a trigger allows unique temporal and spatial control over the gelation process, thus widening its applicability [120].

pH-triggered gelation usually occurs due to the protonation/deprotonation of the amine and carboxyl group in the peptide chain, which results in a shift between the hydrogel and solution state. The ionic peptides are also sensitive to pH changes, which influence the charge on the amino acid. Besides these ionic peptides, other peptides (such as Fmoc peptides) also show responsivity towards pH changes and peptide amphiphile [40,104]. Wang et al. have developed Fmoc- di-Phe and Fmoc-RGD peptides that self-assemble to form pH-sensitive hydrogel with considerable potential for the attachment, proliferation, and multi-differentiation of MSCs [121]. Black et al. have also used a PA (C_16_GSH)-based hydrogel to mimic endogenous ECM for Schwann cells [122]. These hydrogels were biocompatible and biodegradable, with the ability to support angiogenesis. Compared to commercially available collagen gel, PA hydrogel demonstrated improved spreading, proliferation, and migration of the Schwann cells [122].

Light-triggered hydrogels are another exciting category for 3D cell culturing models, as it is very straightforward and convenient to control the source and intensity of the light. Irradiating the peptide solution with a light source results in the sol-gel transition to form a hydrogel. It can also enable physical and chemical changes to mimic the cell microenvironment closely. Furthermore, in addition to triggering the formation of hydrogels, light can also be used to tune the properties and behavior of cells. Light sensitivity is imparted on a peptide sequence by including photoactive moieties such as 2-nitrobenzyl, -C=C- group, and tetrazole moiety [120,123]. Stupp et al. demonstrated that conjugation of a photosensitive moiety, 2-nitrobenzyl with RGD-functionalized PA induced the formation of hydrogels by light-triggered sol-gel transition [124]. It has recently been reported that light-triggered gelation significantly enhanced the encapsulation of NIH/3T3 mouse embryonic fibroblast cells [124].

The presence of divalent ions is also another trigger to stimulate catalytic activity of proteins. In a study, Ca^2+^-triggered structurations of peptide copolymers were reported by which conformational changes can be induced in peptidomimetic structures to improve biological activities and physicochemical properties. High amount of carboxyl groups in peptides have the potential to interact with divalent ions and influence on folding process by changing the size and net charge [116].

Another possible trigger is enzymes, which are abundant in the physiological condition, making enzyme-triggered hydrogels an attractive vista to explore. In the presence of an enzyme, one segment can be cleaved off, leaving a hydrogelator, which can self-assemble into hydrogels. Hydrogelators are a subset of small molecules that can self-assemble in the water to form 3D supramolecular hydrogels [3]. FEFK and FEFKEFK are a type of hydrogel that interacts with metalloproteinase to form the hydrogel. In the presence of the enzyme, the short peptide splits into smaller segments which then reunite to form longer chains that form the gel. These enzyme-triggered hydrogels have been previously reported for osteoblasts and fibroblasts without any detrimental effects from the enzymes [125]. In contrast approach mentioned above, Palocci et al. have reported using lipases from microbial sources to form hydrogelators [126,127]. They studied the use of lipases from different sources of *P. Cepacia* and *Pseudomonas genus,* to develop the hydrogelator Fmoc(Phe)_3_ (tripeptide of Phe) by combining two precursors Fmoc(Phe_3_) and (Phe)_2._ The hydrogelators from both sources could self-assemble into the hydrogel and be biocompatible with rat microglial cells. The hydrogels formed using the *P. genus* also showed enhanced cell proliferation and increased production of the neurotrophic factor [126,127].

Nanostructures based on silk elastin-like polypeptides (SELP) are recognized as stimuli-responsive carriers combining the stimuli-sensitivity and biocompatibility of tropoelastin with the mechanical strength of silk. In a study, the self-assembly capability of these polypeptides and their response to thermal stimuli was evaluated and fabricated nano-gels responded to stimuli through size changes and aggregation [128]. SELPs can be self-assembled in nanostructures by temperature-mediated gelation process useful in different biomedical applications, including drug delivery [129,130,131], gene delivery [131,132,133,134,135,136] stimuli-responsive carriers [137,138], and as a scaffold for tissue engineering [139]. Stimuli-responsive polypeptide-based hydrogels are an attractive candidate as dynamically tunable biomaterials because of the possibility of structural and functional control and genetic tailorability. Recently, a photo-responsive SELP-based hydrogel was reported, and the hydrogels demonstrated a partial collapse of the cross-linked network with decreased loss and storage moduli under visible light [140].

Overall, the biocompatibility and biodegradability associated with peptide hydrogels, along with their versatility, customizable properties, and stimuli responsivity, have made them a promising candidate for biomedical applications.

## 4. Biomedical Applications of Peptide-Based Hydrogels

### 4.1. Targeted Drug Delivery

Targeted treatments allow site-specific delivery of drugs while eliminating unwanted non-specific side effects. In general, non-targeted therapies require a high drug dosage, which leads to more expensive products with higher toxicity. With the advancement in nanotechnology, various systems have been developed to target specific cells and tissues, either actively or passively [141]. Active targeting encompasses targeting moieties attached to the surface of NPs, which can interact with specific targeted tissues [142]. In passive targeting, the nanocarriers are deposited in targeted sites due to distinctive features inherent to the targeted tissues, such as tumor microenvironment, enhanced permeation, and retention effect observed in cancer [143,144]. Polypeptide-based nanosystems offer possible targeted delivery of various cargos [6] and have been explored extensively in targeted cancer therapy and gene delivery, which are discussed here.

Self-assembled peptide nanostructures offer various advantageous properties, such as tailored physicochemical characteristics, surface ligand modification, and high biocompatibility, which have made them a suitable choice for application in active or passive targeted delivery of chemotherapeutic agents [145,146,147]. Self-assembled peptide-based drug delivery systems such as hydrogels, fibers, and NPs have been explored for targeted cancer therapy. Several targeting approaches have been studied based on pH changes, thermal targeting, and using targeting moieties such as RGD, folic acid, gastrin-releasing peptide (GRP), and Tat peptide for nuclear targeting [148,149,150,151].

An example of pH-responsive systems composed of self-assembling peptides was demonstrated by Raza et al., who developed a pH-responsive hydrogel using FER-8 peptide to deliver PXT [152]. The PXT-loaded hydrogel was able to demonstrate a high amount of drug in the tumor sites and prolonged retention time in H22-bearing mice. The drug release is triggered by the degradation of the hydrogel at the acidic pH of the tumor microenvironment. The system demonstrated great potential as targeted cancer therapy by allowing sustained and local drug delivery [152].

Another interesting approach to target tumor cells is phototherapy using phototherapeutic agents, including photodynamic therapy (PDT) or photothermal therapy (PTT). In PDT, irradiation results in the conversion of molecular oxygen to reactive oxygen species, which causes oxidative stress leading to cell death. On the other hand, PPT results in heat generation, which is responsible for cell ablation. Both approaches allow targeted site-specific therapy to a confined area by illumination [153]. A study demonstrated a short peptide-based system that comprises protoporphyrin (PpIX) as the photosensitizer, cell-penetrating peptide (R9), (GPLGLAG), and E_8_ as masking peptide sequence [154]. In the tumor environment, matrix metalloproteinase-2 (MMP-2) is cleaved, removing the masking peptide sequence and exposing the cell-penetrating peptide sequence to interact with the cell membrane. This multistage system allowed the accumulation of the complex at the target site and resulted in significant suppression of tumor size and weight with low systemic toxicity [154]. Similarly, Han and co-workers have reported an MMP-2-a sensitive sequence, which was developed for aggregation-induced emission-guided (AIE) PDT. The system showed preferential accumulation in tumor tissues, with prolonged blood circulation time [155]. A recent example of advanced multifunctional PTT demonstrated by Zhao et al. applied Ag2S quantum dot@polypeptide hybrid hydrogel, which mainly comprises Ag2S quantum dot entrapped in peptide hydrogel composed of expressing RGD (PC_10_ARGD) [156]. These hybrid nanogels showed tumor necrosis and ablation after laser irradiation, leaving black scars at tumor sites and displaying their potential for PTT. This nanosystem has also demonstrated the potential to be used for targeted near-infrared (NIR) II fluorescence imaging, photoacoustic imaging (PAI), and PTT for cancer diagnosis [156]. Overall, polypeptide-based nanostructures have not only been demonstrated to be developed as the targeted delivery system but also used as a multifunctional system, which has several targeting strategies combined hybrid systems, genetically engineered systems, in situ forming systems, pH responsivity, active targeting using ligands, enzyme responsivity, and phototherapy.

### 4.2. Peptide Hydrogels as Templates for Nanofabrication

Peptide hydrogels can provide self-assembling bio-inspired structures, which can spontaneously create 2D and 3D structures. These structures can be used as templates/scaffolds to form nanostructures, including wires, particles, ribbons, tubes, nanoreactors, etc., composed of a wide array of materials such as metal, silica, and polymers [63,157,158,159]. For instance, water-filled peptide nanotubes can act as a template to form nanowires and metallic or polymeric structures. Further, such fabrication can also yield exciting composite materials such as metal-peptide-metal nanowires with unique electromagnetic properties or peptide nanotubes with platinum NPs attached to the walls [63,157,158].

**Figure 2:**
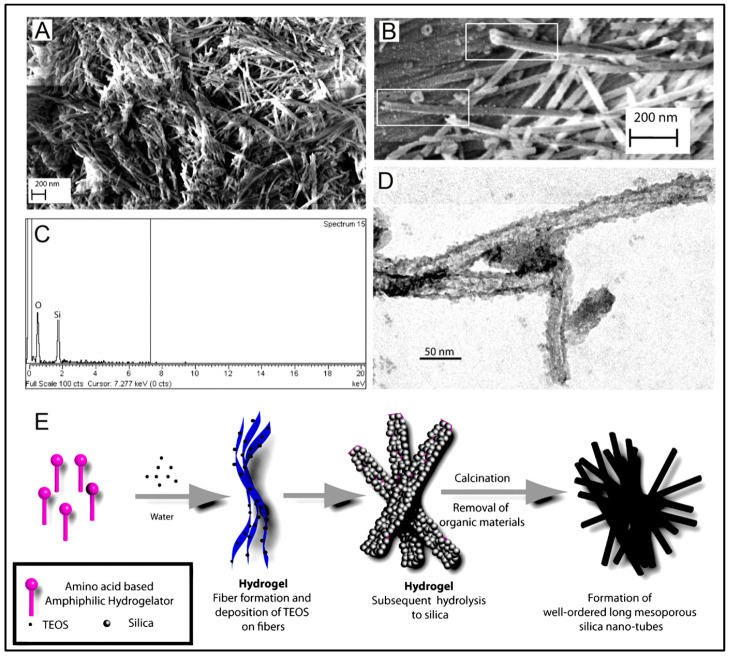
(**A**) Scanning electron microscopy image of the silica nanotubes; (**B**) Field Emission Scanning Electron Microscope image of silica nanotubes showing the open end of the fibers; (**C**) Energy-dispersive X-ray spectrum of the silica nanotubes; (**D**) Transmission electron microscopy image of the silica nanotubes. (**E**) Schematic representation of a possible mechanism of nanotube formation. Reprinted with permission from Ref. [163]; Copyright 2013, American Chemical Society.

With the selective screening of amino acids, self-assembling peptides can be prepared with the ability to bind with metals, whose features can be controlled by peptide sequence and solution composition [160]. An example of such a study is the development of polyaniline polymer-based core-shell nanowires using amyloid nanofiber hydrogel, which can act as a template for nanofabrication [161]. Wang et al. have also demonstrated the formation of long, ultrathin copper (CuS) nanowire using peptide hydrogel as a template [162]. A new hairpin peptide comprising four histidine residues was used, and the self-assembly process was triggered by copper (II) ions. The developed CuS wire demonstrated a near-infrared laser-induced thermal effect [162]. Another study used a simple lysine-based peptide amphiphile linked to a C16 hydrophobic tail to prepare self-assembled nanofibrous hydrogel, which acted as a template to prepare mesoporous single-walled silica nanotubes [163]. The nanotubes were visualized and imaged, as demonstrated in Figure 2A–D. The possible mechanism of silica nanotube formation is schematically depicted in Figure 2E. The silica nanotubes were open-ended and mesoporous, with a few microns long and an average diameter of ~10 nm. Such nanotubes have a wide variety of applicability owing to their remarkable physicochemical properties [163].

Another exciting product that can be assembled by using peptide hydrogels as templates is NPs. A hydrogel template allows the formation of homogenous nano/microstructures with various geometries and sizes, with the ability of drug encapsulation and controlled release kinetics [164]. Adhikari et al. demonstrated using ultrashort peptide hydrogels as a template for in situ formation of Ag nanoclusters by using sunlight [165]. An ultrashort peptide, Fmoc-Val-Asp-OH, was used to prepare transparent and stable Ag-ion-encapsulating hydrogel. These hydrogels can spontaneously produce fluorescent Ag nanoclusters in physiological pH. Under sunlight, the Ag ions were reduced carboxylate group in aspartic acid residues present in the peptide [165]. There are several other ultrashort peptides with many applications, such as bioelectric wires, nanofabrication, bioimaging nanoprobes, etc. [166]. In a study, Jain and co-workers used ultrashort amyloid-based peptides to fabricate gold NPs [167]. The authors were the first to report the crucial role of aliphatic and aromatic -OH moieties of the peptide for in situ synthesis of gold NPs. The shape-controlled nanofabrication aims to prepare the 3D nanostructure of the hydrogel and presents a promising bottom-up approach to produce NPs with tailor-made features [167]. In another study, Reithofer and co-workers demonstrated the synthesis of stable AgNPs within ultrashort peptide (Ac-LK6-NH_2_) hydrogels using UV irradiation [168]. The strict control of size and release of the NP is attainable via peptide hydrogel as the template [168]. Such an AgNP-releasing hydrogel has an enormous scope as an antibacterial agent in wound healing and bioimaging applications. It was demonstrated that the Ag NP composite hydrogel could efficiently inhibit bacterial growth for *Pseudomonas aeruginosa*, *Escherichia coli*, and *Staphylococcus aureus* using only 10 mM Ag NP hydrogels. The biocompatibility studies were also evaluated on primary human dermal fibroblasts, adult (HDFa) cells demonstrating no significant impact on cell viability. This kind of nanocomposite can be recommended for wound healing, especially for chronic wounds, because of its ability to prevent infection, reduce inflammation, and ease of application [165,168].

In a recent article by Zhang e al., an antifouling and sensitive electrochemical biosensor was reported based on multifunctional peptide and urease@zeolite imidazole frameworks (urease@ZIFs) for MMP-7. In this regard, the multifunctional peptide was applied to construct an antifouling electrode interface along with sodium alginate-graphene oxide-Pb^2+^ gel, and then, a carboxyl-rich pyrrole-doped and urease-loaded ZIF coupled with the fabricated electrode interface. Using this biosensor, the conductivity of the sensing interface was significantly decreased as a result of the reaction between Pb^2+^ and CO_2_ (product of urea decomposition). MMP-7 was applied as the model with the ability to recognize specific hydrolytic sites in the multifunctional peptide. This biosensor demonstrated outstanding antifouling performance, high sensitivity, and excellent accuracy for clinical serum samples [169]. In another recent study, Kim et al. reported the design and fabrication of different DNA nanostructures via sequence-specific peptide interactions. Phe- and di-Phe-based monomers were applied to synthesize three different amino acid-based polymers. After coupling to oligonucleotides, they self-assembled into nanofibers, nanosheets, and ribbons via environment-responsive and sequence-specific amino acid interactions. It was shown that the programmable morphology changes could be induced under specific conditions, and it can be helpful in smart drug delivery to release the cargo in response to a particular change in the environment [170]. The influence of physical parameters, including size, shape, mechanical characteristics, surface texture, and compartmentalization on biomaterial design, was reviewed by Mitragotri and Lahann, and they present several examples to show the importance of these parameters in different biomedical applications such as drug delivery, tissue engineering, and imaging [171].

### 4.3. Peptide Hydrogels as Versatile Matrices for 3D Cell Culture

**Figure 3:**
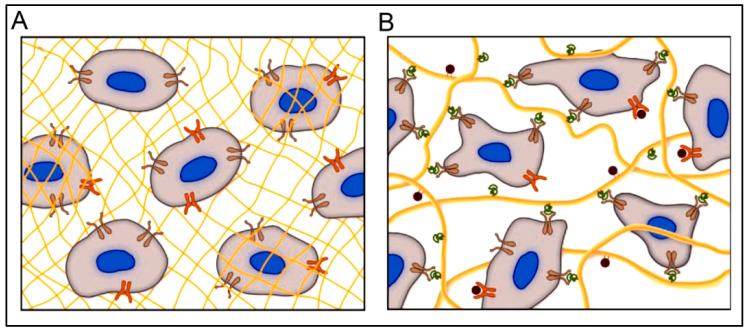
A hydrogel matrix composed of (**A**) synthetic polymers (yellow mesh) provides a 3D environment for culturing cells; however, they fail to activate integrins (brown) and other surface receptors (orange), and (**B**) formed from naturally derived polymers present a myriad of integrin-binding sites (green) and growth factors (red) coordinated to the ECM (yellow fibers). Reprinted with permission from Ref. [174]; Copyright 2009, Wiley-VCH.

Similar to its application as scaffolds for micro- and nano-fabrication, peptide hydrogels could also provide optimal conditions/templates for 3D cell cultures [20,22,40]. Recently, 3D cell culture techniques have been extensively studied owing to their close resemblance to the in vivo cellular environment compared to 2D cell culturing methods and their affordability for in vivo models. Some examples of using 3D cell culturing are related to the differentiation process, drug responses, cell proliferation, signaling process, cellular microenvironment, and cell motility [172]. In current practice, Matrigel and Collagen are the most commonly used matrices. However, using short and self-assembling peptide hydrogels is emerging as a potential matrix for 3D cell culture. A peptide hydrogel, with its cross-linked networks and a large amount of water content, allows for the incorporation of several components (oxygen, nutrients, growth factors), which are essential for imparting many cellular functions [22,173]. Peptide hydrogels are highly versatile and biocompatible/biodegradable and can be easily modified to have tailor-made biological interactions [40]. Furthermore, peptide hydrogels can be developed with a high resemblance to the structure and functions of ECM with optimal design. Compared to conventional polymeric hydrogels, peptide hydrogels have several advantages, such as controllable structure and assembly, reversibility, easy modification, and stimuli responsivity. A schematic representation of different hydrogel types as cell culture matrices is shown in Figure 3 [174]. With the information gathered from the cell microenvironment, cell behavior, and migration, hydrogels can be designed as ECM mimics. Although no single network can completely mimic the complex ECM, bioinspired cues in the hydrogels can help develop diverse and robust 3D scaffolds for different cell culture systems, from which biologically relevant conclusions could be drawn [174].

**Figure 4:**
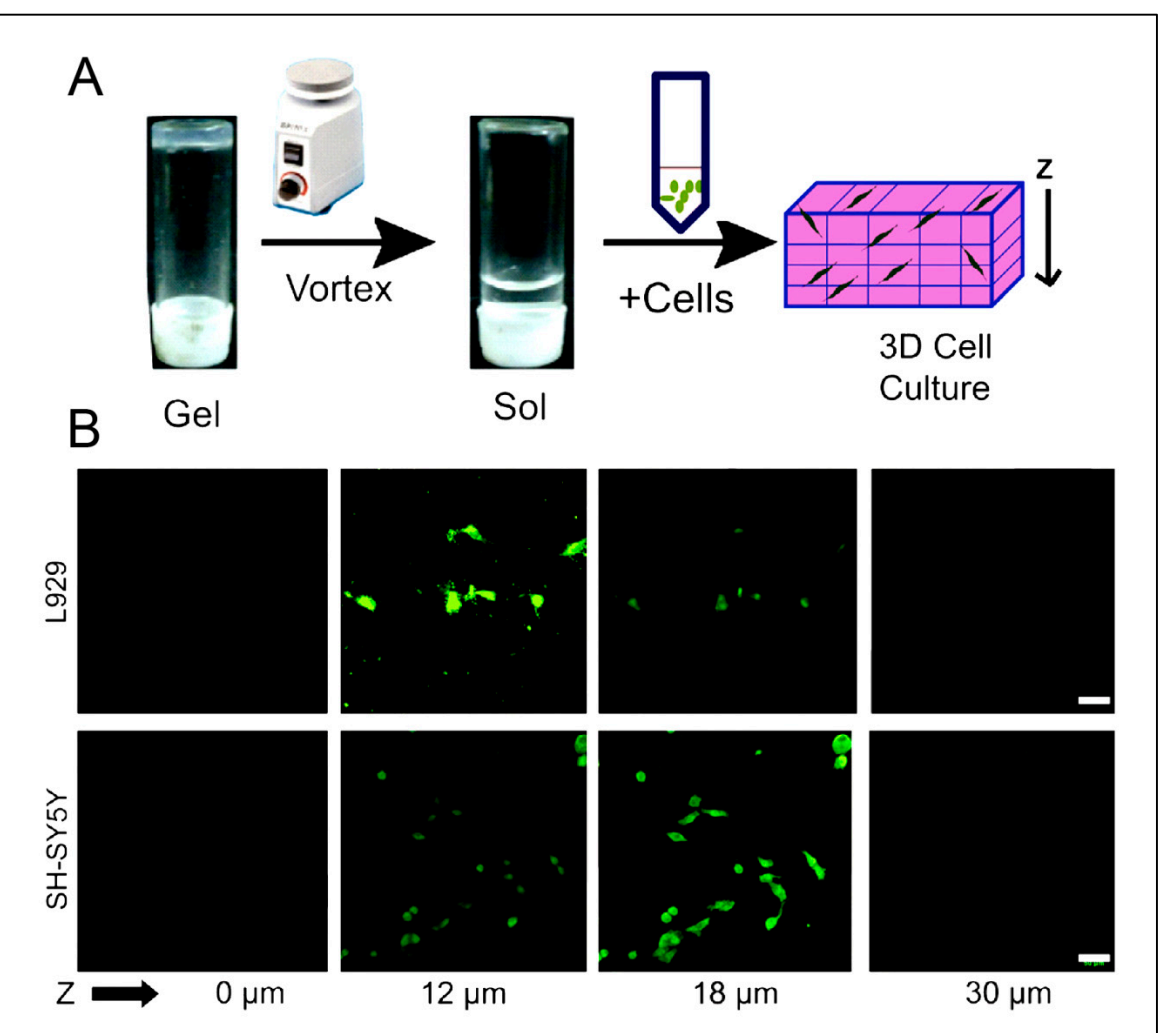
(**A**) Schematic depicting entrapment of cells inside thixotropic peptide gels. (**B**) 3D cell culture using hydrogel showing cell viability of both SH-SY5Y and L929 cells indicated by calcein-AM staining (green) inside the 3D gel matrix. Scale bars are 50 mm. Reprinted with permission from Ref. [178]; Copyright 2015, Elsevier.

Some studies are reporting other types of peptide-based hydrogels. A fascinating study was performed with a novel h9e peptide to form a hydrogel, which can homogenously encapsulate MCF-7 cells. The 3D cell culture model was also successfully used as a carrier for the anticancer drug Cisplatin [175]. Furthermore, the 3D model allowed cell isolation and its downstream proteomic analysis, demonstrating its potential application in drug testing. Again, in another study, the addition of Ca^2+^ions to h9e hydrogels not only promoted the formation of hydrogel and improved gel strength but also imparted a special shear-thinning and recovery property to the hydrogel. This interesting phenomenon is observed due to the Ca^2+^ions occupying the charged Asp residue on the fiber, thus further promoting the inter-fiber interactions of the hydrogel [176]. Several other studies also exploit the rheology of hydrogels. For example, amyloid hydrogel demonstrating thixotropic behavior can be used to homogenously seed cells in the hydrogel matrix [167]. A similar thixotropic effect was also observed in the hydrogel formed through an activated diester building block (formed by reacting PA and p-hydroxybenzyl alcohol in the presence of lipases). This hydrogel was able to encapsulate and promote the proliferation of human umbilical cord MSCs by providing anchorage to cells similar to ECM [40,177]. Similarly, Jacob et al. developed amyloid nanofibril-based hydrogel, with thixotropic properties, for cell culture and stem cell differentiation [178]. The thixotropic property of the hydrogel was used to incorporate cell suspensions with the amyloid gelators using agitation (vortexing). Confocal imaging demonstrated the viability of the cells entrapped in the gels, attributed to its similarity to the natural ECM matrix (Figure 4). Despite showing potential for 3D cell cultures, the use of amyloid hydrogels for in vivo cell culture is limited due to the biocompatibility point of view, as the degraded amyloid peptide may be accumulated in the body [178].

Despite a large volume of research, the use of these hydrogels as 3D templates for cell culture models is still in its preliminary stage. It requires further studies to overcome some of the limitations associated with them, such as the precise control of gelation, mechanical properties, toxicity, the chirality of the hydrogel, the spontaneous release of water, and finding the optimal combination between the type of cells and type of hydrogel. Thus, there is a considerable void in developing the rational design of hydrogelators.

### 4.4. 3D Bioprinting of Peptide Hydrogels

**Figure 5:**
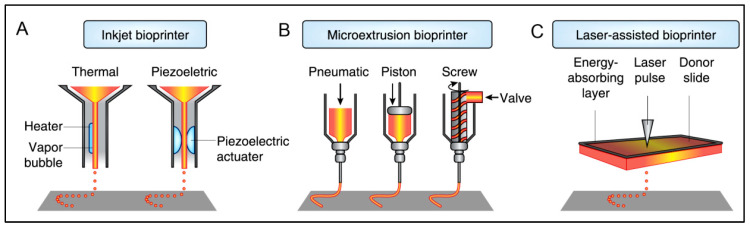
Schematic representation of different types of bioprinting technologies. Reprinted with permission from Ref. [181], Copyright 2014, Nature Publishing Group.

Since its first description in 1986 by Charles Hull, 3D printing has gained much momentum regarding its application in the biomedical field [179]. Types of 3D printing include additive manufacturing, rapid prototyping, or solid-free form. It commences with meshed computer-aided design, which is then used to acquire the product usually formed by layer-by-layer (LbL) addition [180,181]. 3D bioprinting represents a technology that uses biomaterials, cells, and biological molecules to generate 3D constructs/scaffolds and is primarily used for developing organotypic constructs and regenerative medicine [182,183]. The emerging interest in 3D bioprinting is fueled by the high degree of freedom in design, high-precision and reproducible results, and the availability of affordable printers [184]. Several fabrication methods could be applied for 3D printing, such as inkjet printing, extrusion-based, Laser-Induced Forward Transfer, and robotic dispensing, as illustrated in Figure 5 [180,181]. A detailed overview of these methods has been explained elsewhere [184]. In the case of bioprinting, several other advanced techniques with high resolution and reproducibility have emerged, such as cellular inkjet, lithography, and extrusion bioprinters [185].

Among several bioinks, hydrogels have risen as a popular candidate. According to a study by Jungst and co-workers, an ideal hydrogel for 3D printing must have these properties: (i) gelation before printing, with shear-thinning, but not thixotropic, rheology to allow printing, (ii) fast gelation after printing for shape conformity at high resolution, and (iii) minimal swelling of the hydrogel extrude [184,186]. In addition, in the case of bioinks for 3D bioprinting, the hydrogels must localize the cells and provide the environment that ensures the survival and physiological functions of the cells. Further, the bioinks must have instantaneous gelation after printing to preserve the homogenous distribution of the cells in the matrix [185]. Based on these criteria, peptide hydrogels are an ideal candidate to be used as bioink. As discussed previously, peptide hydrogels are very versatile and allow manipulation to add tailor-made characteristics, such as customizable surface features, stimuli-triggered gelation, and controllable mechanical properties. Further, as described in the previous section, peptide hydrogel greatly resembles the native ECM, thus making it a feasible microenvironment for cells to proliferate and function [185]. Thus, peptide hydrogels as bioinks will make 3D scaffolds that are biocompatible and have similar dynamic and complex properties as biological tissue, which is of utmost importance for cells.

**Figure 6:**
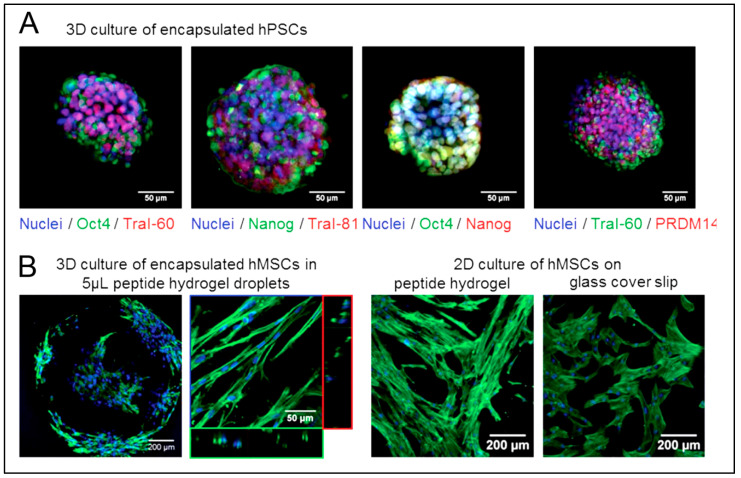
Ultrashort peptide hydrogels encourage the proliferation of encapsulated stem cells for regenerative medicine. (**A**) Human H1 embryonic stem cells encapsulated in 8 mg/mL Ac-ILVAGK-NH2 hydrogels retain their pluripotency, as reflected by the staining of nuclear transcription factors Oct4 and Nanog (red) and surface biomarkers Tra-I-60 and Tra-I-81 (green). (**B**) Human mesenchymal stem cells (hMSCs) encapsulated in 5 μL 10 mg/mL Ac-ILVAGK-NH2 hydrogel droplets and cultured on hydrogel films elongated along the peptide fibers, as reflected by the staining of their actin cytoskeleton (green). On glass coverslips, the cells are well spread out and non-aligned. Reprinted with permission from Ref. [187], Copyright 2015, American Chemical Society.

Despite the many advantages of peptide hydrogels as bioinks, the number of studies is limited. Loo et al. have demonstrated the suitability of self-assembling peptide-based hydrogel as bioinks for constructing 3D scaffolds for cell proliferation and differentiation. Lys-containing hexapeptides used as bioinks can form a 3D scaffold that supports human mesenchymal stem cells (hMSCs) and organotypic differentiation of primary cells [187]. The authors demonstrated the successful proliferation of Human H1 embryonic stem cells (ESCs) into 3D spheroids (Figure 6A) and hMSCs (Figure 6B) when using Lys-containing hexapeptide-based hydrogels as bioink [187]. Raphael et al. described a new and optimized extrusion-based 3D bioprinting method for mammary epithelial cells in a commercially available self-assembling peptide hydrogel (PeptiGelDesign Ltd., Cheshire, United Kingdom). The cells could survive and proliferate in 3D-printed constructs during the seven-day culture [188].

In a recent study by Graham et al., a combination of ultra-low-gelling-temperature (ULGT) agarose and Fmoc protected dipeptide gelators, with or without gelatin, were used as biocompatible bioinks [189]. A low-cost 3D printing technique was used to print aqueous droplets (resolution of 1 nL) of bioink-containing cells (Human embryonic kidney (HEK) cells and ovine MSCs (oMSCs)). The cells remained highly viable in the constructs and retained their biological functions; further, oMSCs were observed to differentiate and generate cartilage-like structures. The size of the constructs is reasonable for their application in high throughput screening techniques. Additionally, the method also allows for the production of higher volumes of the bioinks as well to get larger constructs for its application in printed cellular constructs and disease models [189].

Bioprinting has a lot of potential for developing sophisticated organotypic cultures that could be used for regenerative medicines, implants, and 3D organotypic cell cultures that closely resemble the endogenous environment. The bioprinting technique can be combined with the use of peptide hydrogel as bioinks, thus capitalization on the biomimicry, biocompatibility, and customizable properties afforded by the peptide hydrogels. Nonetheless, several concerns must be addressed when using peptide hydrogels as bioinks. For example, clinical translation of the device would require the construct to be sterile and free of endotoxins.

### 4.5. Molecularly Imprinted Peptide Hydrogels

Molecular imprinting is a technique that fabricates constructs with highly precise chemical architectures with specific target recognition and binding ability by differentiating amongst similar molecules with enantiomeric resolution [190]. The process involves crosslinking of the monomers in the presence of a template, which is later removed to leave a space that fits and could be occupied by the target molecule. In addition to target recognition sites, molecular imprinting also yields stimuli-responsive systems. Molecular imprinting has found its application in different biomedical fields such as chemical sensing, immunoassays, antibody mimicking, artificial enzymes, and catalysis processes [191]. Molecularly imprinted hydrogels have garnered several research interests. However, owing to the inherent movement of hydrogels, there are more challenges than molecularly imprinting solid structures, which could lead to the distortion of the binding sites. Nonetheless, mainly molecularly imprinted polymeric hydrogels have been thoroughly studied for their application in drug delivery with high drug loading or enhanced controlled drug release and in tissue engineering [191,192].

**Figure 7:**
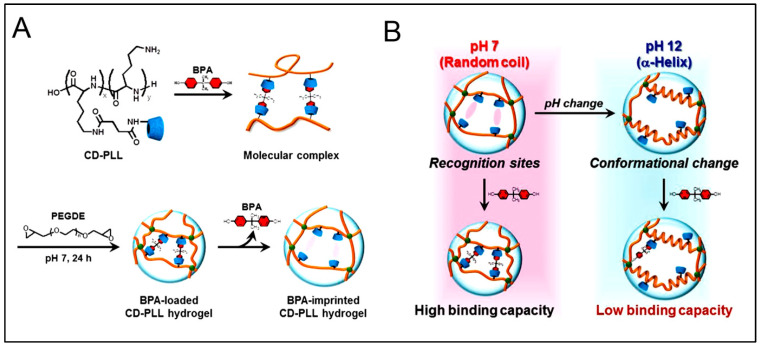
Schematic representation of (**A**) the preparation of the (Bisphenol A) BPA-imprinted β-cyclodextrin-poly(L-lysine) (CD-PLL) hydrogel by molecular imprinting. (**B**) BPA-responsive behavior of the BPA-imprinted CD-PLL hydrogel at neutral or basic pHs. Reprinted with permission from Ref. [193], Copyright 2017, American Chemical Society.

Despite the promising approach, the first mention of the use of molecularly imprinted self-assembling peptides was only in 2016 by Wang and co-workers [121]. Herein, they demonstrated that by using the molecular imprinting technique, the catalytic activity of peptide-based artificial hydrolase could be significantly improved (ca. seven-folds) when compared to a co-assembled system. P-nitrophenyl acetate (pNPA) was used as the template to precisely arrange catalytic residue (Ser/His/Asp) in proper orientation in Fmoc-FF, which assembled to form nanofibers. It was the first time the molecular imprinting method was used to construct enzyme mimetics using self-assembly peptides as supramolecular structures [121]. This study was followed by Matsumoto et al. [193]. In this study, poly(L-Lys) (PLL) was used with β-CDs as ligands. Bisphenol A (BPA) was used as a template molecule (as shown in Figure 7A). The resultant system showed a change in the volume when BPA was added, owing to the complexations between BPA and CD. Further, the hydrogel also demonstrated pH-sensitive BPA adsorption and the stability of the complex, as a pH change resulted in the transition of random coils of the CD-PLL to α-helix and conformational change of the molecular recognition sites (as shown in Figure 7B) [193].

These exciting results established the potential of molecularly imprinted peptide hydrogels as drug carrier systems. However, these systems must be further studied and evaluated for efficacy under in vitro and in vivo conditions. Furthermore, despite observing exciting pH-sensitive binding capacity, the pH conditions tested in this study are not relevant to physiological conditions. Nonetheless, the beneficial properties of both peptide hydrogels and molecular imprinting could be synergized together to develop advanced drug delivery systems and for other biomedical applications.

### 4.6. Cancer Therapy and Immune Regulation

Various self-assembled peptide supramolecular structures have recently been introduced for tumor drug delivery [194,195,196,197]. In a study performed by Nie et al., injectable DOX-loaded hydrogels with antiparallel β-sheet structure were fabricated using a hexapeptide hydrogelator (FEF3K), maintenance of strong π–π interaction between the filaments and sustained-release of DOX. It led to significant tumor growth inhibition in breast cancer mice models while dramatically reducing the side effects of free DOX administration [198]. Kalafatovic et al. developed DOX-loaded peptide-based mixed micelles, which degraded into fibrous nanostructure in response to highly expressed MMP-9 on the MDAMB- 231 turmeric cells surface [199].

Cancer immunotherapy, a newly introduced research field, recruited immunomodulatory agents to increase human immune system activation leading to cancer cell arrest and death [200,201]. The bioavailability and biodegradability of self-assembled peptide structures while performing a controlled drug release make these structures promising candidates to be used in this research field [202,203,204,205,206,207]. While acting as a carrier for delivery of immunomodulatory agents, supramolecular peptide structures can also provide a feasible method to flexibly regulate the immune system individually using their innate characteristics, either in the form of an immune-potentiator or an immune-blocker.

Cyclic dinucleotides, STING (stimulator of interferon genes) agonists, were loaded in a peptide-based nanofibrous injectable hydrogel with sequence K2(SL)6K2 based on electrostatic interactions. Peptide-based hydrogels showed more durable release behavior and increased therapeutic efficacy than collagen hydrogels using head and neck murine tumor models [208].

Dual stimuli-responsive, self-assembled peptide NPs were fabricated and loaded with a short d-peptide antagonist of programmed cell death-ligand 1 (DPPA-1), and an inhibitor of indoleamine 2,3-dioxygenase (NLG919), for effective combinatorial cancer therapy. In this research, functional 3-diethylaminopropyl isothiocyanate (DEAP) molecule, peptide substrate of MMP-2, co-assembled to form amphiphilic peptide NPs encapsulated DPPA-1. NP cargo release happened in response to low pH and high amounts of turmeric site MMP-2. Upon NP administration, simultaneous blockade of immune checkpoints and Trp metabolism caused boosting of the level of tumor-infiltrated cytotoxic T cells, leading to the efficient inhibition of melanoma tumor growth [209].

In a recent study, NPs targeted αvβ3-integrin receptors routinely overexpressed on a tumor cell’s surface. Self-assembled RGD-linked pro-apoptotic peptide coupled with a pH-dependent cyanine 5.5 probes as NIRF-dye was fabricated. Results indicated a significant increase (25.6% to 96.3%) of apoptosis for f-SAPNs, while a decreased degree of necrosis was observed from 51.7% to 0.2% compared with its parent peptide analog (Cy5.5-c [RGDKLAK]; f-CP). NPs also manifested high uptake by U87MG glioblastoma cells suggesting their potential to be recruited in glioblastoma brain tumor theranostic treatment [205].

In another attempt, a combination of immune checkpoint blockade (ICB) with chemotherapeutic drug delivery was performed to hinder tumor progression in B16F10 melanoma xenograft mice models. To do so, co-encapsulation of PTX and immune-adjuvant αGC in liposomes was performed, followed by the modification of liposomes with a pH-sensitive cell penetrating TH peptide. The results indicated a significant increase in the free cholesterol level of blood, and hence, CD8^+^ T cells suppression attenuation, leading to enhanced CTL responses and anti-tumor effects [210].

The delivery of indocyanine green (ICG) and JQ1, a small molecule inhibitor that prevents PD-L1 expression, was conducted using Fmoc-KCRGDK (FK), a self-assembled peptide that is responsible for tumor penetration. The results indicated a strong promotion in dendritic cell maturation and cytotoxic T lymphocytes tumor infiltration upon NIR light-triggered antigen release from peptide hydrogels [211].

### 4.7. Biosensing by Peptide Hydrogels

Clark and Lyons have been the pioneers of biosensors since their inception in the 1960s [212]. Biosensors are devices that “incorporate a biologically active element in intimate contact with an appropriate transduction element to detect (reversibly and selectively) the concentration or activity of chemical species in any type of sample”. There are different types of biosensors, such as enzyme-based, tissue-based, immune-sensors, DNA biosensors, and thermal- and piezoelectric-based biosensors [212]. The increasing use of bioresponsive hydrogels as biosensors can be attributed to the easy manipulation of hydrogel in micro-and nano-patterns to achieve lab-on-a-chip devices [213]. However, using biosensor hydrogels composed of synthetic materials such as polymer will limit its application due to the possible toxicity, degradation, and interaction with the tissue components. These drawbacks can be easily overcome by using small peptide-based hydrogels, which have better predictability and biocompatibility [213].

Self-assembled peptide hydrogels can be used for biosensing by designing them to sense a target molecule, which would trigger self-assembly or disassembly of the hydrogel, or alter the hydrogel properties, thus exhibiting optical, mechanical, or electrochemical outputs [18]. A self-assembled Fmoc-diPhe-based nanofibrous hydrogel used as enzyme-based optical biosensors. In the hydrogel, enzymes and quantum dots were physically immobilized to have enzyme-based biosensing with fluorescent reporters. This study showed the detection of analytes such as glucose and toxic phenolic compounds that were working as an alternative optical biosensing platform with benefits such as simple fabrication, efficient diffusion of target analytes, and high loading of fluorescent reporters and bioreceptors [214]. A more recent study by Fusco et al. used a similar Fmoc (Phe)-based hydrogel to develop an electrochemical biosensor [215]. The results demonstrated the enhanced electrochemical biosensor performance of an Fmoc-Phe3-based hydrogel with Trametes Versicolor Laccase (TvL) immobilized in a hydrogel matrix with gold NPs, as compared to a hydrogel-based graphite biosensor [215]. In another study, an enzyme-based electrochemical biosensor was developed for detecting hydrogen peroxide. Horseradish peroxidase (HRP) was effectively immobilized stably in Fmoc-diPhe peptide hydrogel, which also performed as a robust substrate for cell adhesion. The resultant biosensor demonstrated a low detection limit (18 nM), high stability, and selectivity [216]. Other peptides based on hydrogel have also been introduced in the literature for biosensing. King et al. described an octapeptide (Gly-Gly-Val-Lys-Val-Lys-Val-Glu-Val-Lys) covalently linked to an oligonucleotide CGATTCTGTGTT recognition sequence using thiol-maleimide chemistry. The simple bio-recognition system helps to detect hybridizing DNA using fluorescence as output [217].

**Figure 8:**
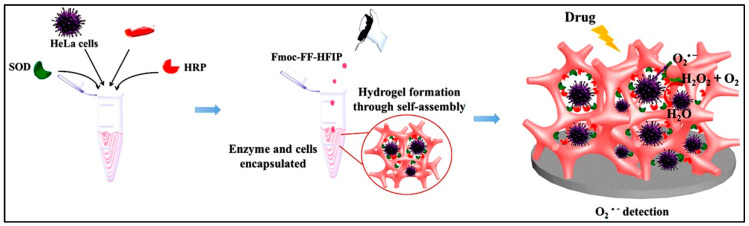
Schematic illustration of constructing a 3D cell culture-based electrochemical platform and its cell-monitoring assay. Reprinted with permission from Ref. [218], Copyright 2017, American Chemical Society.

Another exciting application for biosensors is the detection of the superoxide anions released from the cells in 3D culture in response to drug molecules. Lian and co-workers developed Fmoc dipeptide as a matrix incorporated with HeLa cells and two cascade enzymes, HRP, and superoxide dismutase (SOD), as illustrated in Figure 8. This system demonstrated accurate and sensitive detection of released anions and their generation dynamics under physiological conditions [218].

Regardless of the beneficial application of peptide-based hydrogel as biosensors, the recognition system can be further modified by introducing several functional moieties to enhance the sensing performance. One such approach is the development of hybrid systems by combining peptide hydrogels with metal ions. Gong and co-workers reported a hybrid composite comprising self-assembling dipeptide and gold NPs, with HRP as a model enzyme for electrochemical hydrogen peroxide sensors. This electrochemical biosensing platform demonstrated enhanced performance, attributed to the synergistic effect of the biocompatible dipeptide and good charge transport of the hybrid structure [219].

Overall, with the versatility in the type and stimuli responsiveness in peptide hydrogel, the bright future of hydrogels in biosensors is highly evident. When these properties are aided with transducers, several different outputs can be generated, which can be used to develop ultrasensitive biosensors with varied sensing ranges.

### 4.8. Bioimaging by Peptide Hydrogels

Among biomedical applications, bioimaging is of great importance due to the ability of multi-dimensional visualization of biological processes and biomaterials in living animals. It is recognized as a non-invasive technique enabling to track of biological events in a real-time manner by combining advanced materials with imaging probes [27,220]. It can provide valuable information about signaling networks, biological processes, and pharmaceutical impacts of different materials [27]. In recent years, supramolecular fluorescent hydrogel (SFH)-based bioimaging probes have been introduced for theranostic (therapy and diagnostics) application owing to their biocompatibility, biodegradability, stimuli responsivity, and 3D cross-linked structures [220]. Among these, peptide-based hydrogels are recognized as excellent biomaterials with significant qualities in bioimaging applications [221]. Several studies have shown their advantages in bioimaging. Molecular peptide hydrogelators have fast renal clearance and can cross several body barriers, confirming remarkable biosafety and uptake efficiency. Additionally, peptide hydrogels are programmable due to their responsiveness to different stimuli such as temperature, light, pH, redox, enzymes, and so on [222,223,224,225,226,227]. According to these features, the self-assembly of peptides can potentially be activated under specific stimuli, and the in situ formation of peptide hydrogels have the potential to promote the accumulation of imaging agents in the target regions [228]. So, smart, sensitive, and specific bioimaging will be achieved. Furthermore, peptide hydrogels could increase the stability of bioimaging agents by protecting them from cell excretion to reach a long retention time and sustainable bioimaging [229,230,231]. Hence, peptide-based supramolecular hydrogels are valuable structures in diagnostic applications. There is a report by Xu et al. on the fabrication of SFH using amphiphilic peptide hydrogelators in combination with a fluorophore by which targeted cancer diagnostics, and bioimaging can be performed [232]. This kind of designed hydrogels can respond to specific stimuli in the cancer microenvironment, and they can also act as drug carriers instead of imaging probe. Zhang et al. discovered the formation of Trp–Phe dipeptide NPs (DNPs) that can transfer the peptide’s inherent fluorescent signal from the ultraviolet to the visible range. The DNPs are modified with MUC1 aptamers, which enable the recognition of the overexpressed MUC1 proteins located on the membrane of A549 human carcinoma epithelial cells for cancer targeting and biosensing [233]. Another study reported that a simple dipeptide, di-phe, self-assembled into various ordered structures, could produce intense photoluminescence with emission maxima at em = 450 nm [234].

In recent years, the application of peptide hydrogels was established in different bioimaging techniques such as magnetic resonance imaging (MRI) [235], PAI [236,237], optical imaging (OI) [238,239,240,241], computed tomography (CT) [242], radionuclide imaging (RI) [243,244], and ultrasound imaging (USI) [245]. To improve the sensitivity and specificity of these modalities, bioimaging agents such as contrast agents, fluorophores, and radioactive isotopes are applied [246,247,248,249]. For instance, the nanostructure of superparamagnetic iron oxide NPs (SPION) with peptide-based nanomaterials was applied to target lesion sites by targeting the capability of the peptide. A study has reported a strategy for the improvement of MRI using fluorescence-labeled SPION conjugated with CREKA, a fibrin-binding peptide, to perform molecular imaging of microthrombus by enhancing T1 relaxation time [250]. In another study, Liang et al. reported an activable hydrogelator with the enzyme-responsive ability for MRI imaging. Alkaline phosphatase is known as an overexpressed enzyme in some number of malignant tumor cells and can activate the hydrogelator via dephosphorylation. The activation and subsequent self-assembly of the hydrogelator into nanofibers resulted in the enhancement of T_2_-weighted MRI [251]. Peptide hydrogels also demonstrate outstanding potential in OI or fluorescence imaging by carrying fluorescent dyes. For example, Wang et al. designed and fabricated a NIR dye-conjugated peptide hydrogelator diagnosing cancer-associated fibroblasts. This suggested hydrogelator was activated by the fibroblast activation protein-α and formed nanofibers by the self-assembly process. Surprisingly, nanofibers’ formation improves the blood circulation time in comparison to ICG and had a strong fluorescence signal even 2 days after intravenous injection [240]. Radionuclides can also be encapsulated in the peptide hydrogels that are essential for RI by positron emission tomography (PET) and single photon emission CT (SPECT) [27]. Oyen et al. designed an ^111^in-based peptide hydrogelator using covalent conjugation of DOTA-chelated ^111^In with a hexapeptide amphiphile. It was shown that in vivo visualization of drug release could be achieved by SPECT imaging using this designed structure [244]. CT is another bioimaging technique with the capability to produce anatomic structures in clinical detection and provide high resolution images of hard tissues. An iodinated peptide hydrogel was suggested to detect bacterial alkaline phosphatase (ALP) activity using nano-CT. The peptide hydrogelator Nap-Phe-Phe(I)-Tyr(H_2_PO_3_)-OH was prepared, and after exposure to ALP, it was activated by dephosphorylation and self-assembled into hydrogels. This approach could lead to accumulation of iodine on the surface of bacteria as an ALP-rich region and provide high CT contrast [242]. New research indicated that imaging sensitivity, specificity, and efficiency could be enhanced via peptide-based photoacoustic tomography contrast agents [252,253]. A smart ICG-encapsulated peptide hydrogel was prepared by Huang et al. for in vivo tumor PAI. The designed peptide hydrogelator had the potential to be activated by phosphatase-induced dephosphorylation and self-assembled to nanofibers. It was revealed that this nanostructure could enhance PAI signals with higher tumor accumulation, longer retention time, and stronger FL signals compared to free ICG [237].

In summary, peptide hydrogels exhibit high biocompatibility, prominent loading capacity, and long intracellular retention, presenting several advantages to improve the properties of bioimaging agents under different modalities. The hydrogelators can be smartly activated by certain environment stimuli and spontaneously self-assemble into nanofibers and then form hydrogels. So, the imaging agents could accumulate in the stimuli-rich region, consequently exhibiting amplified signals and prolonged retention. Therefore, precise, sensitive, and sustainable in vivo bioimaging of biological events could be achieved using smart peptide-based supramolecular hydrogels.

### 4.9. Stem Cell Therapy (Transplantation) by Peptide Hydrogels

Stem cells (SCs) are specialized potential cells in different tissues that can perpetuate and differentiate to diverse cell types in a tissue or organ, or act as carriers for complex signal delivery. There are different types of SCs, from an origin point of view, including embryonic and adult SCs, of which adult ones are usually found in particular ECM conditions resulting in proliferation and differentiation, as well as cell specialization when needed [254,255]. The first idea of SC therapy was initiated in 1998 by successfully creating ESC [256]. Much research has been carried out on SCs in different fields, including cancer therapy, tissue engineering, cardiac diseases, osteoarthritis, diabetes, regenerative medicine, and neurological disorders [255,257]. According to them, it is suggested that SCs are a promising therapeutic candidate in regenerative applications [255]. For example, pluripotent SCs (PSCs) can differentiate into specified cell lines relying on signal cascades and micro-environmental cues. In contrast, in the lack of mesodermal and endodermal signals, ESCs can be converted to neural cell lines. Besides, mice- or human-isolated hematopoietic SCs (HSCs) can generate immune cells and bone marrow cells. Despite all progress, SC therapy encounters challenges in clinical applications, such as uncontrolled differentiation and functional engraftment of the implanted tissue. To overcome these limitations, cell-based systems that mimic ECM are necessary to establish and 3D assemblies of SC [255]. Recently, numerous biocompatible materials have been introduced as support scaffolds for the 3D culture of SCs, primarily porous materials for the preparation of appropriate 3D micro-environment used for cell growth, growth factor availability, and environmental communication between cells and cells with ECM. So, biocompatibility should first be evaluated in different conditions when researchers design and fabricate a scaffold with efficient mechanical and chemical features and without induction of inflammatory responses. Besides, biodegradability, inert construction, and decoration by immobilized biological components are also concentrated more attention as essential parameters. SCs have sparked a great interest in regenerating injured tissue and organs in spinal cord injury, epilepsy, or neurodegenerative diseases. Many 3D scaffolds in literature are hydrogels prepared from natural materials, hydrophilic or hydrophobic nanomaterials [255]. SC-niche interaction needs to be effectively regulated in tissue regeneration and has been possible via cell-cell and cell-ECM connections as well as the existence of growth factors. It is proved that glycoproteins and proteoglycans cause these actions, which are composed of half of the ECM proteins [258].

As promised by traditional methods in regenerative medicine, hydrogel-based materials have typically been employed as carriers for cell or growth factors [259]. There is a plethora of reports that highlight the advantages of peptide hydrogels to circumvent the limitations of traditional strategies. Some of them are mentioned below and classified by types of SCs:

#### 4.9.1. Neural SCs (NSCs) Transplantation and Delivery

**Figure 9:**
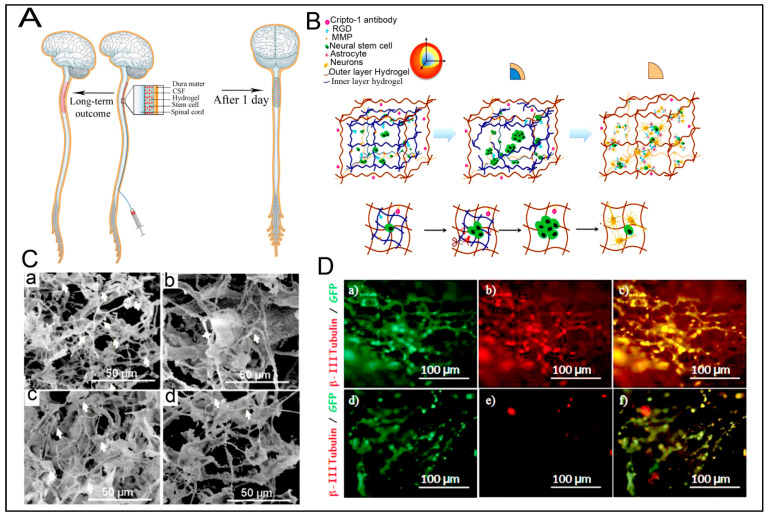
Hydrogel-based scaffolds to support and deliver neural stem cells (NSCs). (**A**) Schematic representation of hydrogel injection encapsulated NSCs into the spinal cord and short- and long-term outcomes. Reproduced with permission from Ref. [257], Copyright 2018, Springer Nature. (**B**) An illustration of intelligent double-layer hydrogel along with matrix metalloproteinases (MMP) and arginine-glycine-aspartate (RGD) peptides at the inner layer and Cripto-1 antibody at the outer layer for NSCs delivery and proliferation. (**C**): (**a**–**d**) Scanning electron microscopy (SEM) images of differentiated NSCs cultured with 50 μg.mL^−1^ of Cripto-1 antibody hydrogel. (**D**) Promoting the survival and differentiation of NSCs transplanted in vivo by the designed hydrogel. (**a**,**d**) are GFP staining, (**b**,**e**) represent β-III Tubulin, and (**c**,**f**) demonstrate the merged channels of GFP and β-III Tubulin. Reprinted with permission from Ref. [261], Copyright 2018, Wiley VCH.

Patients with neurological diseases are travailing from the functional deterioration of the central nervous system (CNS) due to cellular degeneration and death. Plenty of efforts have been recently directed towards developing several approaches for cellular regeneration. It has been demonstrated that SC transplantation can play a therapeutic role in mice when transplanted cells are broadly distributed in the CNS. CH_3_CO-(Arg-Ala-Asp-Ala)_4_-CONH_2_ (ac-(RADA)_4_-CONH_2_) hydrogelator peptides can encapsulate NSC and endow them with the differentiation possibility into neurons, neural progenitor cells, astrocytes, and oligodendrocytes. It is demonstrated that neural cells can survive for at least five weeks, so for long-term studies, self-assembled peptide hydrogel is strongly recommended [260]. Since there is a 1000 times size difference between mouse and human brains, SC therapy needs efficient, targeted delivery to the broad areas of the CNS. Recently, hydrogels have been introduced as appropriate scaffolding biomaterials for SC therapy to provide ECM proteins directing cell fates during migration, differentiation, and regeneration in the CNS. Besides, hydrogels can mimic the SC niche. They have attracted the interest of researchers to use these systems for SC delivery which is achievable due to the ability of hydrogels in precise localization and controlled cell delivery (Figure 9A) [257]. Due to acute inflammatory and immune responses during transplant surgery or SC therapy, the survival of transplanted cells is one of the critical issues. An efficient hydrogel system needs to circumvent this challenge in treating CNS-related disorders. Qiao et al. suggested a smart, double-layer, alginate hydrogels-grafted MMP and RGD polypeptides at the inner layer and Crypto-1 antibody to the outer layer for NSC delivery (Figure 9B). The proliferation of NSCs was carried out in the inner layer obtained by immobilized RGD peptide. Furthermore, blocking Crypto-1 was done via a Crypto-1 antibody in the outer materials by which the differentiation of dopaminergic neurons was improved in vitro. When the inflammatory storm was finished, the NSCs were found at the outer layer because of MMP secreted by transplanted SC to cut the MMP polypeptide on the inner layer. As a result, an NSC culture on a designed hydrogel demonstrated considerable neuronal differentiation and neurite formation and over-expression of the genes related to dopaminergic neurons (Figure 9C,D) [261].

**Figure 10:**
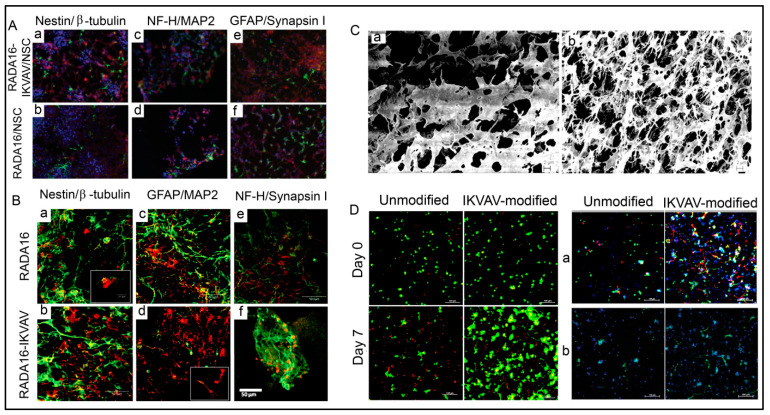
NSCs encapsulation in hydrogel for brain tissue regeneration. (**A**) Immunohistochemistry of the damaged brain tissues at six weeks after transplantation staining of Nesttin/β-tubulin (**a**,**b**), NF-H/MAP2 (**c**,**d**), and GFAP/Synapsin I (**e**,**f**). DAPI was used to stain the nuclei. (**B**) Immunohistochemistry of NSCs encapsulated in peptide hydrogel after two weeks. Cells were stained with protein markers such as Nestin (green) for neural progenitor, GFAP (green) for astrocytes, β-tubulin, MAP2 (red) for premature and mature neurons, NF-H (green), and Synapsin I (red) for neural cytoskeleton, and neurotransmitters. The results has been shown for RADA16 (**a**,**c**,**e**) and RADA16-IKVAV (**b**,**d**,**f**). Reprinted with permission from Ref. [259], Copyright 2013, Elsevier. (**C**) field-emission SEM (FE-SEM) images of unmodified (**a**) and IKVAV-modified (**b**) silk fibroin hydrogel (Scale bar: 2 μm). (**D**) Live/dead assay of hNSCs encapsulated in hydrogels at 0 and seven days with confocal microscopy (Scale bar: 100 μm); and immunohistochemistry of hNSCs stained with (**a**) Nestin (green) and βIII-tubulin (red) and (**b**) MAP-2 (green) and GFAP (red) after 7 days (Scale bar: 100 μm). Reprinted with permission from Ref. [265], Copyright 2017, Wiley VCH.

The adult brain’s poor regenerative ability seeks more research on effective long-term approaches for neurological brain deficit, including cell transplantation. The self-renewal and differentiation potential of NSCs benefits providing diverse neural cells in CNS, and bioactive scaffolds can guarantee cell viability and cell differentiation for transplantation therapy. Self-assembled peptide RADA16 modified by laminin-derived peptide isoleucine-lysine-valine-alanine-valine (IKVAV) was evaluated to provide a functional 3D hydrogel accelerating CNS regeneration. It can be helpful in enhancing the renovation of the damaged brain. It was shown that the in situ hydrogel formation was performed immediately after the designed peptide injection, and RADA16-IKVAV hydrogels could give birth to NSC, reduce glial astrocytes content and induce differentiation into neural cells. Six weeks of post-transplantation studies revealed that the neural differentiation was supported by hydrogels (Figure 10A,B) [259]. The 12-amino acid IKVAV motif present on the α-laminin chain is well recognized as an effective factor on the expression of various markers for cell adhesion, proliferation, differentiation, and migration as well as neurite outgrowth [262,263]. The formation of neural cells is not limited to NSCs. Other stem cells, such as ESCs, are also able to differentiate into neural cells, as was shown by Li et al. They utilized a RADA-based 3D matrix modified with IKVAV peptide to guide ESCs undergoing neuronal differentiation without any soluble additive. IKVAV peptides also can inhibit glial scar formation and subsequently increase neural regeneration [264].

Injectable hydrogels were proposed due to their advantage in replacing the injured environment during the cell therapy process and inhibiting more harm in surgery. For this purpose, owing to low immunogenic and inflammatory responses, silk fibroin-based injectable hydrogels immobilized with the IKVAV as a critical component in the brain ECM were prepared to encapsulate human NSCs (hNSCs) used in brain tissue engineering. It was proved that cell encapsulation in IKVAV-modified hydrogel increased cell viability and growth rate compared to unmodified hydrogels because of the more open, porous structure of modified hydrogel or cell-adhesive activity of IKVAV peptide. Additionally, modified hydrogels provided higher ratios of neurons at seven days after differentiation, but the modification of hydrogels did not result in improving the length of neurite outgrowths (Figure 10C,D) [265].

**Figure 11:**
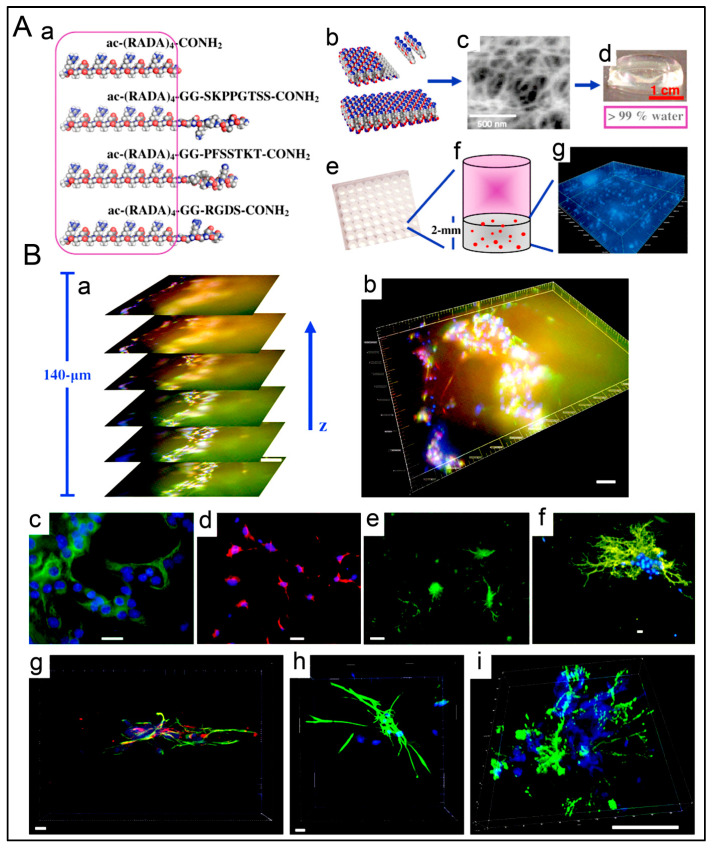
Three-dimensional NSC cultures in peptide hydrogels, Matrigel and Collagen I. (**A**) (**a**): Molecular structures of ac-(RADA_4_-CONH_2_ and modified peptides with di-glycine linker and the functional motifs. (**b**): Peptide nanofiber formation by self-assembly of the peptide monomers. (**c**): SEM image of the peptide nanofiber. (**d**): Transplant peptide hydrogels. (**e**,**f**): Schematic of 96-well plate and a well with peptide hydrogel-encapsulated cells. (**g**): Microscopy image of NSCs encapsulated in the peptide hydrogels. (**B**) Inverted fluorescence microscopy (**a**–**f**) and confocal microscopy (**g**–**i**) images of NSCs cultured in ac-(RADA)_4_-GG-SKPPGTSS-CONH_2_ hydrogel: (**a**,**b**): after 2-week culture, nestin(+) neural progenitors, cells (green) and Tuj1(+) neurons (red) appear at different z-planes; (**c**,**g**): Stained neural progenitors with anti-nestin (green), (**d**,**g**): Stained neurons with anti-Tuj1 (red), (**e**,**h**): Stained astrocytes with anti-GFAP (green), and (**f**,**i**): Stained oligodendrocytes with anti-GalC (green). Cell nuclei were stained with DAPI (blue). (Scale bar: 200 μm (**a**,**b**) and 20 μm in images (**c**–**i**)). Reprinted with permission from Ref. [260], Copyright 2013, Elsevier.

The encapsulation of NSCs was studied into ac-(RADA)_4_ self-assembled peptide hydrogels as 3D neural tissue culture exhibited the differentiation of NSCs into neural cells (neurons, astrocytes, and oligodendrocytes) (Figure 11A). To compare peptide hydrogels, Collagen I, and Matrigel, cell survival experiments were performed, and it was observed that adult mouse NSCs cultured in collagen I scaffold formed clusters and were poorly differentiated. In contrast, Matrigel and peptide hydrogels promoted NSC proliferation and differentiation for 1–2 weeks and at least five months, respectively. So, peptide hydrogels showed better cell survival rates and differentiation (Figure 11B) [260].

Degeneration or promoting neural repair is known to be influenced by neurotrophic growth factors with some limitations such as short half-life and susceptibility to enzymatic degradation, and the presence of BBB against large molecules. So, there is a substantial gap to replace traditional strategies prolonging in situ glial cell-derived neurotrophic growth factor (GDNF) in damaged sites. An N-fFmoc self-assembled peptide was proposed by Rodriguez et al. [266] as a hierarchical scaffold with the purpose of growth factor stabilization and promoting stem cell integration for Parkinson’s disease treatment. The Fmoc-DIKVAV with sustained release of GDNF during one week exhibited spatial and temporal delivery in the rodent model and simulated the niche environment for grafted cells [266]. In stem cell therapy, the cells firstly need to attach and spread on the synthetic scaffolds that it is possible by a specific bioactive peptide derived from the ECM or a non-specific cationic cell adhesive motif with the contribution to cellular adhesion and differentiation. It was assumed that the combination of both approaches in a hydrogel might improve cell adhesion in a lower concentration of RGD peptide. Interestingly, a synergistic effect was exerted on NSC adhesion and differentiation compared to a GRGDSF-peptide alone or the cationic motif. The mechanism of cell adhesion was firstly by electrostatic interactions promoted by cationic motif on the negative surface of PCM, and integrin-receptor interactions were observed [267].

Following spinal cord injury, glial scarring and post-traumatic inflammation are common challenges that self-assembled peptides can overcome. For this purpose, K_2_(OL)_6_K_2_ (QL6) is introduced because of the ability to self-assemble into β-sheets at physiological pH and support neural growth and spinal cord injury repair. QL6 injection revealed decreased apoptosis, glial scarring, and attenuated inflammation. It was also caused to improve axonal conduction and tissue preservation at spinal cord injury [268]. Moreover, Iwasaki et al. demonstrated the synergistic effect of transplanted neural stem/ progenitor cells and injection of self-assembled QL6 on an injury of the cervical spinal cord. At 12 weeks after injury, tissue preservation was significantly observed alongside forelimb neurobehavioral recovery [269].

#### 4.9.2. Hematopoietic Stem Cell Transplantation

HSCs with the potential for self-renewal and differentiation have sparked more interest in SC therapy. The differentiation of PSC toward different hematopoietic cells has provided a novel method as an alternative for bone marrow transplantation. It should be noted that the cell-cell interactions and ECM proteins such as laminin and collagen play an appreciable role in embryonic development and need to be considered in suggested approaches. Shan et al. proposed a self-assembled peptide hydrogel combined with hematopoietic cytokines as a 3D superstructure to generate hematopoietic cells in vitro from small-model PSCs. It was first shown that hematopoietic differentiation was induced by apparent expression of specific markers such as c-kit, CD41, and CD45 in mouse PSCs (mPSCs) into the designed hydrogels by which a 3D environment was supplied for mPSCs differentiation. Furthermore, multi-potential progenitor cells could be developed by mPSCs differentiation. Among them, prepared HSCs on a 3D system could potentially differentiate into lymphocyte cells. The in vivo experiments in mice revealed the short-term engraftment potential of mPSCs, which are efficiently embedded into NOD/SCID mice after three weeks of transplantation [270].

#### 4.9.3. Multipotent Skin-Derived Precursors (SKPs)

**Figure 12:**
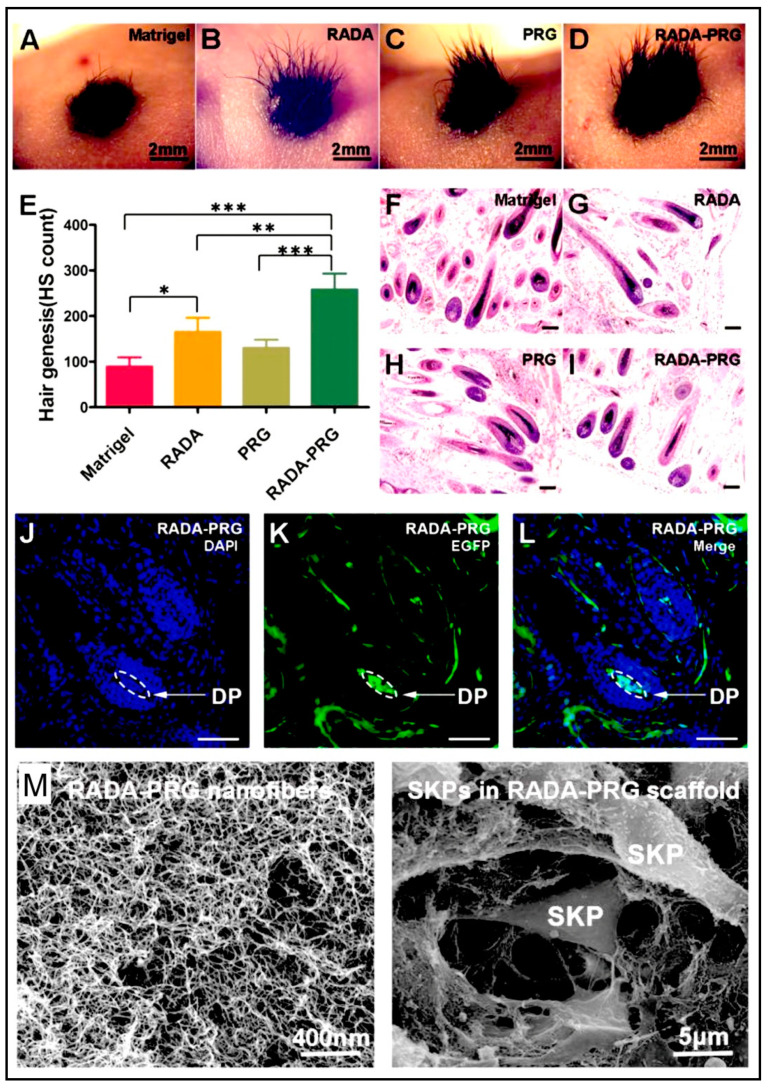
De novo hair follicle regeneration by self-assembling peptide hydrogel. (**A**–**D**) Illustration of hairs genesis after three weeks post-transplantation of neonatal mouse epidermal cells and skin-derived precursors (SKPs) in Matrigel and different peptide hydrogels into excisional wounds in nude mice. (**E**) Calculation of the number of hair shafts and the average number of hair shafts per wound (n = 4, * *p* < 0.05, ** *p* < 0.01, *** *p* < 0.001). (**F**–**L**) Histological analysis of the wound tissue after three weeks post-transplantation of neonatal mouse epidermal cells and SKPs labeled with EGFP gene in Matrigel and different peptide scaffolds. (**J**–**L**). Immunofluorescence analysis showed that SKPs contributed to the dermal papilla in the hair follicle and numerous cells (green) in the dermis. Representative images of wounds receiving transplanted cells in RADA-PRG hydrogel. (**M**) SEM images of RADA-PRG scaffold (left) and its interactions with SKPs (right). Reprinted with permission from Ref. [230], Copyright 2016, Elsevier.

Skin as the first defense against infections and fluid loss is the largest vital organ in the body [271]. Skin SCs induced by hair follicle neogenesis, reside in the dermis of the skin and need appropriate signals and a particular niche for skin morphogenesis [271]. In addition to seeded stem cells, scaffolds also play climacteric roles by mimicking the SC niche to promote cell survival, differentiation, and functional tissue formation. This fact obligates researchers to find suitable materials as a support for SCs during organogenesis. Self-assembled peptide hydrogels formed by RADA16 and PRG and a combination of them enhanced the proliferation of SKPs. It is noteworthy that the SKP encapsulated RADA-PRG hydrogel exhibited enhanced cell survival and proliferation, and the expression of hair genesis genes in vitro and de novo hair biogenesis. Also, they had a better result in cell adhesion. In this proposed scaffold, the RADA16 peptide helped the formation of adequate nanofiber and PRG rendered the integrin-binding motif, and increased cell survival and hair follicle biogenesis (Figure 12) [230].

#### 4.9.4. Mesenchymal Stem Cells (MSCs)

Owing to self-renewal and multi-differentiation capability into mesenchymal and non-mesenchymal cells, hMSCs have directed scientists towards combining them with biomaterials used in culturing and delivery [272]. In principle, culture dimensions directly influence cell behavior and differentiation of stem cells at various matrix rigidities. This behavior stems from the differences between 2D and 3D systems, including mass transport, cell adhesion, cell-cell, and cell-biomaterial interactions, as well as mechano-transduction [273]. Self-assembled peptide hydrogels composed of fibrous KFE-8/KFE-RGE are suitable biomimetic substrates to directly compare 2D and 3D matrices studied by Hogrebe and Gooch on hMSCs differentiation [274]. In the various matrix stiffness, constant RGD peptide, and similar inducers, 2D culturing facilitated efficient osteogenesis, while chondrogenesis has resulted in encapsulation in 3D scaffolds. It was shown that at given stiffness, adipogenesis was induced by 3D matrix better than 2D culture. This study proved that matrix dimensionality and stiffness play an essential role in tissue engineering [274]. The combination of 2D and 3D systems exhibited a promising scaffold that the surface features of the 2D design can be effectively transcripted in 3D cultures. An injecTable 3D composite composed of graphene oxide (GO) and polypeptide thermogel was proposed for adipogenic differentiation of MSCs derived from tonsils, by which the expression of adipogenic biomarkers was enhanced. Additionally, the incorporated MSCs supplied insulin as an adipogenic differentiation factor, which was adhered to GO, partially denatured in the presence of GO, and participated in adipogenic differentiation. In conclusion, the suggested thermogel composite could provide tissue volume and a 3D matrix for adipogenic differentiation [273].

Another main factor in hydrogel development is their bio-functionality reached by functional sequences such as RGD and its analog, RGE, peptides. Due to the very hydrophilic nature of RGD peptides, mixing with F-moc-diPhe increased the stability of hydrogelators. A 3D self-assembled network of Fmoc-diPhe and F-moc-RGD peptides was fabricated to present bioactive moieties at the hydrogel surface appropriate for cell adhesion. Furthermore, proliferation and cell survival were increased by F-moc-diPhe/Fmoc-RGD hydrogel in the case of MSCs-induced osteogenic, adipogenic, and chondrogenic differentiation in both in vitro and in vivo conditions. It is proved that as-prepared hydrogels can improve cell attachment, proliferation, and multi-differentiation useful for musculoskeletal tissue engineering because of the viable cell culturing and a mild immune response [275].

Under certain conditions, neurons and glial cells can be developed by differentiating bone marrow-derived MSCs in vitro and in vivo. Bone marrow MSCs modified by neurotrophic factor genes have unveiled higher biocompatibility and differentiation potential when embedded in a functionalized peptide hydrogel. The modification of MSCs was investigated by specific surface markers such as CD90, CD29, and CD45. RADA16-PRG hydrogel was synthesized as a carrier for modified MSCs. Cell growth and proliferation, the expression level of growth factor mRNA, neuron-specific enolase (NSE), and glial fibrillary acidic protein were evaluated to determine hydrogel efficiency. It is observed that the modified hydrogels compared with control revealed a significant increase in gene expression, cell growth and proliferation, and enhancement of NSE and reporter protein levels. Luo et al. concluded that bone marrow-derived MSCs were differentiated into neural cells when they were modified with neurotrophic growth factor and seeded in RADA16-PRG peptide hydrogels. There was a synergetic relationship between gene modification and designed hydrogels valid for spinal cord injury [276].

**Figure 13:**
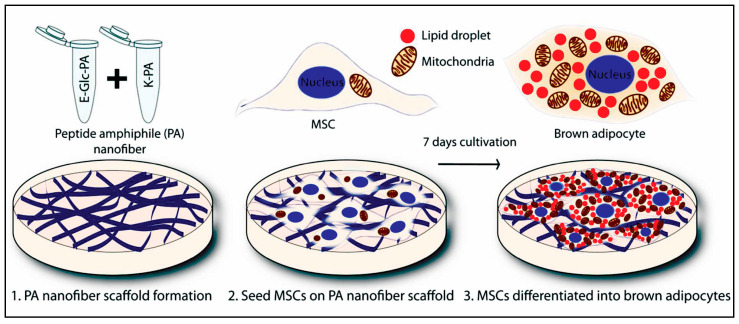
Schematic representation of supramolecular glycopeptide nanofibers for MSCs differentiation into brown adipocytes. Reprinted with permission from Ref. [278], Copyright 2017, American Chemical Society.

The functionalization of peptide hydrogels via spatial organization can significantly influence cell functions and biological mechanisms [277]. In this regard, peptide hydrogels can be designed and fabricated for diverse applications relying on the bioactive peptide epitopes. Considering the roles of saccharides in cell attachment and the effect of glycosaminoglycans in ECM, bioactive glycol-PA (Glc-PA) were used to create peptide nanofibers, including E-Glc-PA and K-Glc-PA with flanking amino acids of Glu and Lys, respectively, placed around bioactive sequences. Then, the influence of spatial organization was investigated on cellular responses of rat-derived MSCs cultures on designed PA nanofibers. It was demonstrated that the brown fat adipogenesis of MSCs significantly induced by E-Glc-PA/K-PA rather than K-Glc-PA/E-PA and controls. As a result, the spatial organization of bioactive groups developed different conformational signals by which a significant difference was created in MSCs behavior. The suggested cost-effective supramolecular structure revealed a great potential to culture SCs and tune the differentiation profile into mature brown adipocytes without the requirement for any other differentiation factors (Figure 13) [278].

#### 4.9.5. Embryonic Stem Cells

Patients with sensorineural hearing loss often have a problem in their inner ear. In these cases, the use of cochlear implants can be promising as standard care with the regeneration of spiral ganglion neurons (SGNs) of the cochlea [279]. The implantation of human ESCs into the inner ear can promote the regeneration of SGNs in animal models. At the same time in the clinic, the efficiency of cell production, differentiation, niche condition, and long-term survival should be improved [280]. Among all, supportive cell niches play a vital role in regulating SC proliferation, differentiation, and survival. For this reason, self-assembled PAs are known as appropriate structures to construct a functional niche and meet needs as mentioned earlier. Self-assembled PAs are affected by hydrogen bonds between amino acids and physiological conditions such as Ca^2+^ concentrations to form 3D gels, by which cell migration outgrowth is prepared. It is demonstrated that IKVAV-PA gels are functional biomaterials to develop a robust SC niche in vitro and in vivo and enhance the survival and differentiation of hESC-derived otic neural progenitor cells in the inner ear. Additionally, IKVAV-PA gels were successfully injected into the human cadaveric temporal bone and revealed positive effects for SC delivery in the clinic. So, the combination of ESC transplantation with injectable PA gels is suggested to create an appropriate microenvironment for inner ear regeneration [281].

#### 4.9.6. Pluripotent Stem Cells Regenerative Application

Induced PSCs (iPSCs) attracted a great deal of attention regarding their potential applications in regenerative medicine for transplantation, in vitro disease modeling, and drug screening [282]. They are remarkable potential bio-systems able to differentiate into osteoblasts and osteocytes [283]. In recent years, bone tissue engineering has been introduced as a novel strategy for repairing bone defects and combining SCs and growth factors with a porous biocompatible and biodegradable scaffold. In a study, in vivo bone regeneration was investigated through iPSCs delivered in self-assembled 16-amino acid peptide hydrogel. Micro-CT analysis demonstrated an increased regeneration in bone tissue with iPSC seeded hydrogels compared to a salt solution or nanofiber scaffold [284].

**Figure 14:**
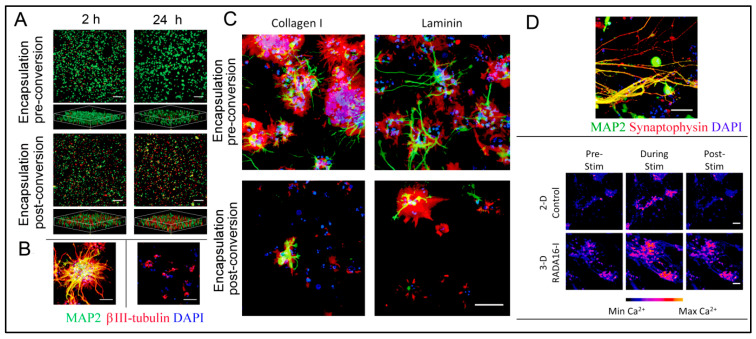
Reprogramming and transplantation of hPSC-derived neurons supported by SAPNS hydrogels. (**A**) Live/dead assay of cells encapsulated in RADA16-I SAPNS hydrogels. (**B**) Immunochemical assay revealed neurite outgrowth at pre-induction and post-induction. (**C**) Secretion of collagen I and laminin by hydrogel-encapsulated iPSCs. (**D**) Immunocytochemical assay of neuron maturing with synaptophysin expression (**up**) and live-cell calcium images from time-lapse videos (**bottom**). Reprinted with permission from Ref. [286], Copyright 2016, American Chemical Society.

In recent years, the transplanTable 3D scaffolds have attracted more attention due to their potential to support cells and providing required surviving conditions and mechanical maintenance [285]. It has been previously shown that human neurons can be derived from human iPSCs by cell reprogramming. Accordingly, RADA16-I-based nanofibrous scaffolds were designed as a 3D in vitro niche to evaluate the reprogramming and maturation of iPSC-derived neurons by lentiviral-mediated transfection [286]. Also, a microfluidic-based method was used to fabricate RADA16-I microspheres for in vivo delivery of neurons into a mouse brain for the first time. It is demonstrated that the encapsulation of human infected iPSC (iPSC-RN) was successfully carried out in designed hydrogels, and eight days after induction, robust neurite outgrowth and expression of neural markers were observed (Figure 14A,B). Despite the lack of bioactive domains in the RADA16-I hydrogel, it could improve the proliferation of iPSCs by which the secretion of collagen I and laminin as the ECM proteins was possible (Figure 14C). However, ECM protein secretion was stopped after neural induction, along with limitations in viability and neurite outgrowth. Electrical stimuli 12 days after induction were responded to by 89 ± 3.4% of encapsulated neurons exhibiting the scaffold’s ability to mature the induced neurons functionally (Figure 14D). Finally, microfluidic-base synthesized RADA16-I microspheres were injected into the striatum of NOD-SCID IL2Rγc mice, and in vivo cell survival was screened three weeks after transplantation to show the potential of the designed hydrogel as a neural transplantation vehicle. Overall, RADA16-I hydrogels were introduced as an effective scaffold in reprogramming and transplantation processes and promising for in vivo regenerative medicine [286].

### 4.10. Peptide-Based Hydrogel Assemblies for Wound Regeneration

Wound healing is an intricate process comprising a plethora of cellular and molecular stages, including the homeostasis, inflammatory, proliferative (granulation, contraction, and epithelialization), and maturation (remodeling) phases [287,288,289,290,291]. Due to certain conditions such as bacterial infection, renal disease, ischemia, diabetes, and local hypoxia developing the complex wound with a length of healing time, and life-threatening ability, the focus of fundamental research is more on antimicrobial properties and cost-effective materials [288]. Proteolytic enzymes such as elastase, MMP, plasmin, and reactive oxygen and nitrogen species (ROS & RNS) are exuded from chronic wounds. They are interrupting the balance between the degradation and maturation process and also changing the oxidant/antioxidant condition in non-healing cells. It is previously studied that ROS can be served as an MMP inducer and cause necrosis and permanent damage in injured tissues. To solve this problem, Barros et al. proposed that the decline in ROS/RNS and elastase levels can pave the road for chronic wounds to accelerate wound healing via normal process [288]. They successfully found small peptides including Pep_4_ (KRCCPDTCGIKCL) and Pep_4_M (KRMMPDTMGIKML) from the antimicrobial domain of an endogenous elastase inhibitor useful in wound dressing applications [288].

Providing a moist environment, inhibiting secondary infections, absorbing the wound exudates, reducing wound necrosis, preventing wound desiccation, and the delivery and stimulation of the growth factors are the main features to select a desirable wound dressing [287]. Considering the potential of biodegradable and biocompatible hydrogels in swelling, in situ gelling, small molecule delivery, and their hydrophilicity, there is a plethora of reports that have developed hydrogel-based platforms to heal various wounds [292,293,294]. The swelling capability minimizes the risk of bacterial infections since it avoids the formation of fluid-filled pockets. Likewise, in situ gelling can form a cross-linked network and provide a platform to control small molecule (drugs or growth factors) delivery and the complete closure of the wound. Tending toward the fabrication of healing materials with antimicrobial properties has attracted more attention to antimicrobial agents such as antibiotics, AgNPs, and antimicrobial biopeptides with potent inhibition activity against a broad range of bacteria [295]. The hydrogel wound dressing is an essential application of hydrogels, concentrating more attention on the wound and burn management [287].

Owing to biocompatibility, chemical and physical degradation, high water content, porosity, mechanical strength, and tissue-like viscoelasticity, as well as cell adhesion properties, peptide hydrogels have attracted the interest of the scientific community for biomedical applications. Some reported hydrogels have exhibited good mechanical features and biodegradability useful to wound sites for tissue repair [296,297,298]. Hydrogels with a 3D network structure and tunable physicochemical properties have opened a new gateway in biomedical fields, including commercial wound dressing, contact lenses, and hygiene products [299]. Various commercial products are introduced in the market to aid external wound healing. For instance, the products formulated with hydrocolloids, alginate, polyurethane, silicone, or nylon can be mentioned. However, the investigation of new materials becomes the focus of fundamental research for wound healing applications. In this regard, hydrogels formed by synthetic polymers, especially peptide-derived hydrogels, have sparked great interest in tissue regeneration. In recent years, a series of peptide sequences with three to seven amino acid residues are identified able to be self-assembled in water and form cost-effective, non-immunogenic, elastic, and biocompatible hydrogels [290]. Self-assembling peptides are forming various reversible supramolecular structures including fibrils, membranes, and gels by weak non-covalent interactions and mimic the natural fibrillar proteins in ECM [294]. Aggregation of these structures results in 3D networks revealed the capability to support cell growth and differentiation for in vitro and in vivo applications [300] and as carriers for transplanted cells. As an example, Ac-(RARADADA)2-CONH_2_and Ac-(RADA)4-CONH_2_ are organized β-sheet superstructures known as appropriate scaffolds for wound healing, 3D culture, and synapse growth [301].

In the case of peptide-derived hydrogels, the optimization of different sequences is necessary to find nanostructures with greater stiffness, elasticity, and shape fidelity in water after a prolonged period. As an example, cysteine-mediated disulfide cross-links introduced to ultrasmall peptide sequence by which the conjugation of peptide fiber to bioactive signals and gels purification were quickly possible. Furthermore, their elasticity and shape maintenance were rendered by oxidation. Using these features, the prepared peptide gels were esteemed as a candidate for diverse applications in regenerative therapies, especially in wound healing [302]. Consequently, several hydrogels based on a Cys-containing peptide, LIVAGKC, were considered to further the characterization suitable for skin applications, in particular, efficiency in cutaneous wound healing as an ideal cost-effective, straightforward, and easy to handle dressing with a removal ability in comparison to commercial DuoDerm and Tegaderm products. The cross-linked LIVAGKC functionalized with CRGD endowed these properties. In this study, it was unveiled that the properties of peptide-based hydrogels can be controlled via crucial mutation at a single amino acid residue; thereby, each prepared sequence exhibited unique physical properties, including gelation concentration, stiffness, elasticity, and transparency. Measuring the rate of water lost from the gel sample proved that peptide nanofibers impressively entrapped water to moisten the wound for at least seven days without physical changes in gels. In vivo investigations in mice suggested accelerated wound healing by topical usage of peptide hydrogels infused with a supplemented medium by serum and growth factors [290].

The last decade has witnessed increased interest in applying self-assembled peptide hydrogels as a promising alternative to the traditional scaffold in regeneration medicine. They spontaneously form nanofibers and subsequently a scaffold-like tissue-bridging structure. Peptide self-assembly can be further served as regenerative agents for soft tissues (e.g., blood vessels and skin), forming new blood vessels from existing ones through angiogenesis [4,303]. Nanoscale peptide fibers can induce a direct interaction between peptide scaffold, ECM, and damaged tissue on both sides of the lesion, allowing cell migration into the scaffold. For instance, one of the ionic self-complementary peptides, the RADA16-I peptide, has attracted plenty of attention due to its capability to promote neural cell proliferation and synapse formation, fill wound sites, and repair injured optical pathways. RADA16-I can confer several features such as the formation of a nanofiber network similar to ECM to provide an in vivo cell growth microenvironment; degradation to L-amino acids utilizable by circumambient tissue; lack of biological and chemical contaminants common in animal-derived materials like collagen; and avoiding the tissue rejection due to non-immunogenic responses [297]. However, the acidic pH of RADA16-I can restrict further broad applications because it may damage the cells and host tissues in 3D culture. To enhance the efficiency and overcome this limitation, Sun et al. proposed a stable nanofiber hydrogel composed of IKVAV from laminin and the sequence RGD from fibronectin at natural pH [294]. Contrary to RADA16-I, 3D-IKVAV/-RGD presented high viability in neural progenitor cells/stem cells without the need for a growth factor for differentiation. In addition, nerve regeneration and promotion of myelination were proved in vivo on Schwann cells by designed hydrogels compared to RADA 16-I [298]. Loo et al. [292] introduced an intelligent L-lysine-containing peptide hydrogel with an innate tendency to self-assembly and formation of helical fibers. So, it can be a perfect building block to accelerate wound closure in vivo in rat burn model as they maintain an ideal water hydration condition for partial-thickness burn wound healing. On these terms, they carried out a comparative study between two peptide-based hydrogels and a standard-of-care (Mepitel^®^, a silicon-coated polyamide net). Early exploration demonstrated earlier onset and completion of autolytic debridement to promote epithermal and dermal regeneration without the need for exogenous growth factors. Hypertrophic scar formation and infection risks play a pivotal role in determining the rate of wound closure. It was shown that the ultrashort peptide hydrogels filled a niche sorely neglected by current treatment options. In this case, to enhance wound healing, the regenerative properties can be further increased by the incorporation of bioactive moieties such as drugs, antimicrobials, growth factors, and cytokines. It is noteworthy that peptide hydrogels are more highlighted as scaffolds for skin regeneration able to serve in case of deep, partial, and full-thickness burns with a non-immunogenic response. Aiming to develop cost-effective “just-add-water” formulations, the high stable hydrogels at room temperature can be reconstituted by adding a specific volume of clean water to lyophilized peptide powder [292].

Xie et al. reported an in situ formation of novel biodegradable hydrogels using a copolymer network of poly(ethylene glycol) maleate citrate (PEGMC) and poly(ethylene glycol) diacrylate (PEGDA) as a biodegradable dressing. It can be advantageous to conform to the skin wound shape and prevent bacterial invasion in vitro and in vivo in rats using functionalization by antimicrobial peptides including CHRG01, ABU-CHRG01 (ABU), TemporinA (TEMP-A), and Ala5-Tritrp7 (ALA5). They successfully developed an optimized hydrogel with ideal mechanical and physical performance, biodegradability, and antimicrobial properties, achievable due to biocidal peptides. The feasible usage of prepared hydrogels on the rat skin wound model was illustrated by a preliminary in vivo study that promoted wound healing and prevented infections [295].

**Figure 15:**
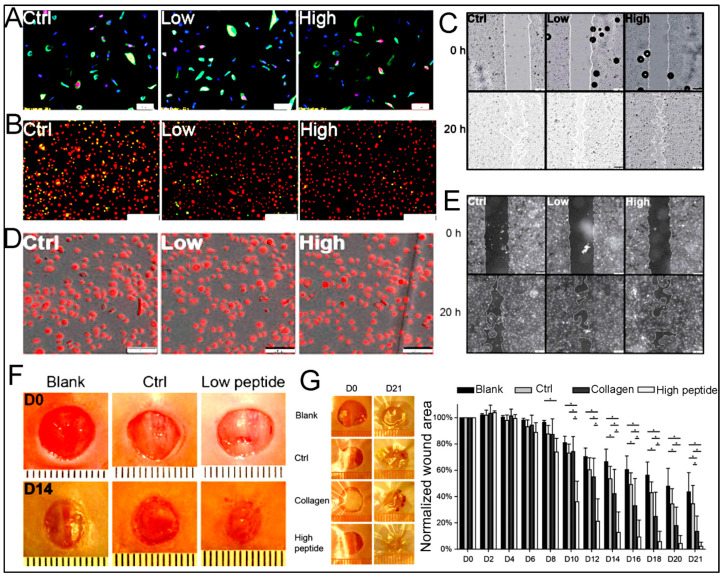
Regeneration and re-epithelialization evaluation of non-healing diabetic wounds with peptide hydrogels treatment. (**A**) Kct-positive human epidermal keratinocytes (HEK) cultured in low (100 μM) and high (650 μM) concentrations of QHREDGS peptide exhibited no significant amount of BrdU and similar proliferation rates in all samples. (Scale bar: 50 μm; Kct: green; BrdU: red; and DAPI: blue). (**B**) The EarlyTox Cell integrity assay demonstrated HEK survival after hydrogen peroxide treatment due to peptide protection (Scale bar: 200 μm). (**C**) HEK-wounding experiments on designed films in different concentrations of the conjugated peptide by which HEK migration was accelerated in a concentration-dependent manner (scale bar: 200 μm). (**D**) DHEK attachment was demonstrated on the chitosan-collagen films in different concentrations of QHREDGS peptide (scale bar: 200 μm). The presence of the peptide caused the promotion of DHEK attachment on chitosan films rather than on chitosan-collagen films. (**E**) DHEK wounding experiment on designed films immobilized with peptide (scale bar: 200 μm). DHEK migration was increased with peptide in comparison with control. (**F**) Gross images of the initial wounds on days 0 and 14 treated with no hydrogel, peptide-free hydrogel, and low-dose peptide-immobilized hydrogel. (**G**) Gross images of initial wounds on days 0 and 21 treated by no hydrogel, peptide-free hydrogel, ColActive collagen dressing, and High dose peptide immobilized hydrogel. Wound size measurement showed an accelerated wound closure in treatment with peptide. Reprinted with permission from Ref. [304], Copyright 2016, National Academy of Sciences.

Xiao et al. [304] proposed a wound healing approachable to promote keratinocyte migration, protect the cells against ROS, and change the ECM to improve cell attachment. The glutamine-histidine-arginine-glutamic acid-aspartic acid-glycine-serine (QHREDGS) peptide was introduced as an angiopoietin-1–derived peptide capable of interacting with integrins with a plethora of applications such as endothelial cell metabolism enhancement, neonatal rat cardiomyocyte attachment, survival promotion, osteoblast matrix deposition, and mineralization induction. It is assumed that QHREDGS peptide can also function in diabetic wound healing. According to the hypothesis, the QHREDGS peptide within chitosan–collagen film coating immobilized on normal and diabetic keratinocytes to find its influence on the attachment, survival, and migration of cells. It is also assessed in the diabetic mice model to prove wound repair promotion in vivo. The in vitro study on normal neonatal human epidermal keratinocytes (HEKs) illustrated no significant effect of the peptide on proliferation and migration rate (Figure 15A). At the same time, the HEKs under oxidative stress presented a dose-dependent survival increase in the presence of QHREDGS (Figure 15B). To enhance the efficiency, the QHREDGS peptide was covalently immobilized to a chitosan-collagen hydrogel. It is indicated that the HEK attachment was promoted by the QHREDGS peptide but the presence of collagen masks this impact. Due to the importance of keratinocytes migration in wound healing, the effect of the immobilized peptide was assessed on HEK migration and confirmed the influential role of the immobilized QHREDGS peptide to accelerate collective HEK migration in a dose-dependent manner (Figure 15C). Likewise, the immobilized QHREDGS peptide was examined on adult diabetic HEKs (DHEKs) and found similar results in the promotion of DHEK attachment to chitosan-only films as shown in Figure 15D. The effect of immobilized QHREDGS peptide on DHEK survival under oxidative stress conditions showed significant improvement in DHEK survival (Figure 15E). The capability of immobilized QHREDGS peptide to accelerate wound healing in diabetic mice models was also shown and it resulted in more minor wounds after 14 days of therapy (Figure 15F). To compare the proposed system with an FDA-approved treatment, the potential of a high-peptide hydrogel against CloActive collagen dressing was demonstrated in vivo, and closed wounds were found after 21 days in the high-peptide hydrogels (Figure 15G). As a result, the QHREDGS peptide can act as an alternative candidate to promote diabetic wound healing [304].

To treat the chronic wound defect, injectable self-assembled peptide (RADA-16) hydrogels conjugated with substance P were proposed for skin regeneration in diabetic rat models. Furthermore, substance P (an 11-amino acid neuropeptide) used in designed hydrogels remained in the wound area for three weeks due to its homing effect and mobilized the endogenous MSCs from bone marrow to wound sites which had a therapeutic impact on the wound. It is confirmed that wound healing was promoted by P conjugated-self assembled peptide with increased mobilization of MSCs and without cell transplantation in the type I diabetic model [305].

Connexin43 (Cx43) is identified by pre-clinical studies as a therapeutic target in dermal wound healing that can accelerate wound re-epithelialization. In a survey by Grek et al. [306], a 25-amino acid synthetic peptide mimetic of the C-terminus of Cx43, ACT1, was evaluated on non-healing neuropathic diabetic foot ulcers with standard care treated in adults with ulcers of at least four weeks duration. It was concluded that ACT1 embedded in a hydroxyethyl-cellulose hydrogel could significantly reduce the ulcer area, accelerate wound closure, and cause 100% ulcer re-epithelialization without adverse effects and any immunogenic responses. Likewise, the combination of adipose tissue-derived stem cells’ activity in wound healing and Exendin-4 (a glucagon-like peptide-1 receptor agonist) effects on diabetes revealed an accelerated reduction in wound size and skin reconstruction. As a result, migration, invasion, and proliferation were surpassed in human endothelial cells and keratinocytes via combination therapy of Ex-4 and SCs [306]. These proof-of-concept studies can be utilized for further experiments in the wound healing process and use the mentioned peptides in superstructures such as hydrogels.

#### 4.10.1. Peptide Hydrogel as a Tool in Angiogenesis-Mediated Wound Healing

**Figure 16:**
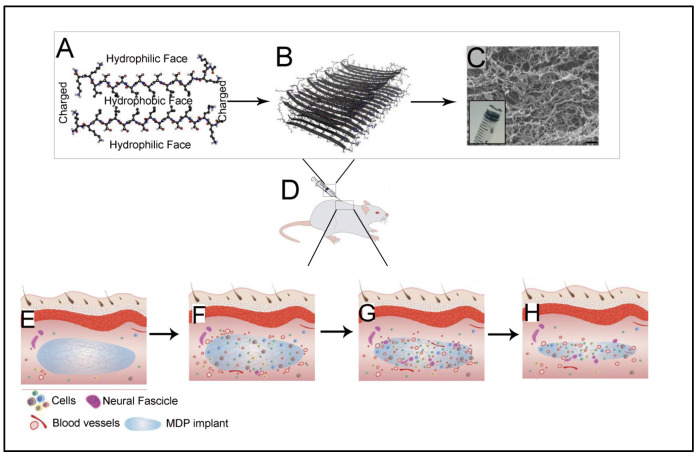
Angiogenesis and neurogenesis elicitation by nanofibrous peptide hydrogel. (**A**) A dimer of K_2_(SL)_6_K_2_ peptides represents a hydrophobic sandwich; (**B**) Nanofiber formation by self-assembled K_2_(SL)_6_K_2_ peptides; (**C**) SEM representation of the K_2_(SL)_6_K_2_ hydrogel (Scale bar: 200 nm); (**D**) Schematic illustration of hydrogel injection into dorsal tissue; (**E**–**H**) Hydrogel remodeling representation during six weeks initiated with cell infiltration followed by angiogenesis and innervations and finishing with slow biodegradation. Reprinted with permission from Ref. [293], Copyright 2018, Elsevier.

Tissue regeneration is strongly dependent on angiogenesis. This process plays an essential role in diverse wound healing [307]. It is proven that the semi-occlusive usage of hydrogel dressing can initiate the angiogenesis process due to temporary hypoxia, by which the growth of tissue in an adequate supply of oxygen and nutrients to the wound surface is ensured [287]. For instance, Moore et al. suggested a simple nanofibrous peptide (K_2_(SL)_6_K_2_) hydrogel innately promoting a robust angiogenic response in vivo while containing no drugs and proteins, nor cells or bioactive sequence. It is finely revealed that this implantable hydrogel can infiltrate many target cells, provoke an inflammatory reaction in a few days and result in the maturation and remodeling process (Figure 16). The selected amino acid sequence was an amphiphilic peptide with self-assembling property in an aqueous solution by which β-sheet secondary structure could form nanofibers acting as native ECM. Due to the inflammatory response and hydrogel density, the peptide hydrogel has sparked great interest as a candidate material for tissue regeneration applications [293].

In another study, owing to self-assembled ability, antimicrobial activity, feasible modification for biological activities, and ECM mimetic behavior, the placental growth factor-1(PlGF-1)-loaded supramolecular composite hydrogel generated by multi-domain peptide nanofibers and liposomes attracted a great deal of attention acting as time-controlled drug delivery systems. The fabricated composite was examined for PlGF-1 release and its ability in spatial- and temporal-controlled angiogenesis induction in vitro and in vivo. It is proved that the modulation of HUVEC VEGF receptor activation in vitro and mature blood vasculature in vivo is endowed by MDP(Lipo(PlGF-1)) implant. There is scientific consensus on MLCs ability to serve as matrices for tissue regeneration, by which high levels of cellular infiltration and preparation of suitable scaffold for stable vasculature are possible [308].

#### 4.10.2. Antibacterial Peptide Hydrogel for Wound Healing

**Figure 17:**
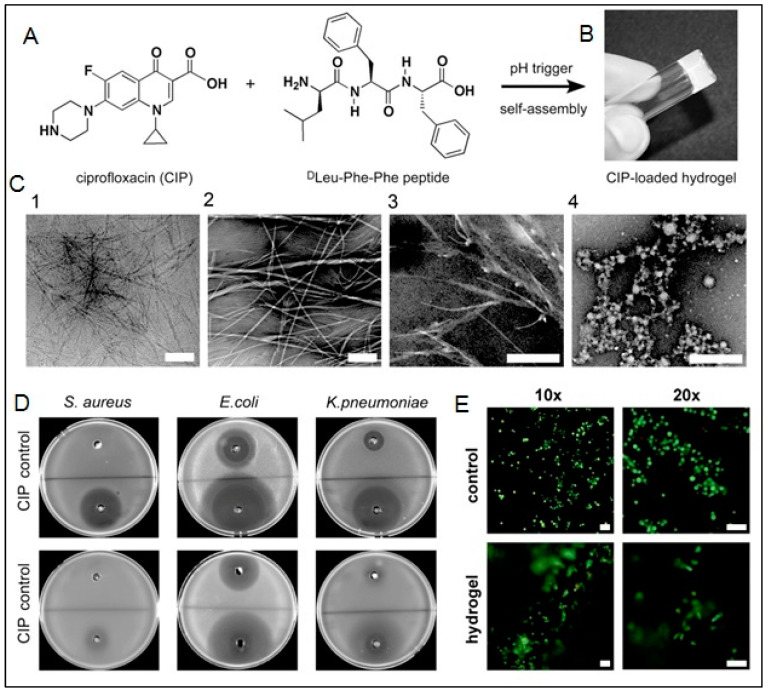
Design and fabrication of antimicrobial nano-hydrogel. (**A**) Chemical structure of ciprofloxacin (CIP) and tripeptide ^D^Leu-Phe-Phe; (**B**) hydrogel formation by self-assembly with pH change; (**C**) Cryo-TEM (**C1**) and TEM (**C2**–**C4**) images of hydrogels loaded by CIP (**C1**,**C3**,**C4**) and tripeptide (**C2**) (Scale bar: 200 nm). (**D**) Microgel well diffusion assay measured the antibacterial efficacy of CIP-gel on *Staphylococcus aureus*, *Escherichia coli,* and *Klebsiella pneumonia* at low (**top**) and high (**bottom**) bacterial densities; (**E**) Live/dead assay for L929 fibroblast cultures on hydrogel or plastic used as control after three days (Scale bar: 50 μm). Reprinted with permission from Ref. [310], Copyright 2013, Elsevier.

Chronic and acute wounds are seldom hampered by infections developed initially by gram-positive bacteria as a normal skin biota. After more than four weeks, gram-negative bacteria result in chronic infections [309]. In recent years, most studies have concentrated on research efforts to develop cost-effective, antibacterial substances for wound dressing. The utilization of nanotechnology in the treatment of wound infections attracted tremendous attention due to its high surface area-to-volume ratio, controlled physical properties (porosity, size, shape, etc.), and feasible sustained drug release. Fluoroquinolone ciprofloxacin, as an effective antibiotic, has been used widely in topical applications, including skin and eye infections. For the first time, Marchesan et al. [310] reported the self-assembly of soluble antibiotic and a hydrophobic tripeptide (^D^Leu-Phe-Phe) to prepare a macroscopic hydrogel with high drug loading (30% *w*/*w*). It could act as a novel stable antibacterial formulations and control release of the ciprofloxacin. The antibacterial effect of a self-assembled hydrogel loaded by ciprofloxacin was evaluated against *Staphylococcus aureus*, *Escherichia coli*, and *Klebsiella pneumonia* exhibited sustained drug release. It should be mentioned that the hydrogels alone also revealed a mild antibacterial effect on gram-negative bacteria without producing undesirable side effects or cytotoxicity on L929 fibroblast cells. According to these results, the designed nanostructure can prolong drug release and manage wound healing infections (Figure 17) [310]. Besides, antibacterial NPs such as AgNPs become the focus of some research in the case of wound management and antibacterial Ag-hydrogels prepared by various materials via different approaches. Among all, self-assembled Ag-doped diPhe-constructed hydrogels demonstrated an Ag dose-dependent antibacterial activity against *Staphylococcus aureus* with a synergistic combination of antibacterial properties of Ag and structural features of hydrogels. There was no antibacterial activity in hydrogels without silver. The proposed hydrogel not only plays a key role in ECM simulation to entrap Ag but also can prepare the moist environment to promote accelerated wound healing [311].

Antimicrobial hydrogels are prepared by cationic polymers or peptides, by which immunogenicity and inflammation, as well as cytotoxicity to mammalian cells, are concluded. To circumvent these challenges, supramolecular biocompatible and fibrous hydrogels were synthesized via self-assembly of oligopeptides modified by a 9-fluorenylmethoxycarbonylin aqueous solution. So, modified phe as a novel hydrogelator was co-assembled with antibacterial peptides to generate antibacterial hydrogels with selective Gram-positive antibacterial activity to treat wound infections’ bacteria [312].

In wound healing, wound infection resistance to some antibacterial treatments is more concerning [309]. Due to their fibrous architecture and high water content, as well as ECM-like functionality, self-assembled peptides are considered promising candidates for wound dressing. Furthermore, ultrashort self-assembled peptides are introduced that have an extra advantage of antibacterial activity. As an example, Lacerty et al. reported an ultrashort cationic dipeptide conjugated to naphthalene with antimicrobial properties to prevent infections in wound healing. The antibacterial activity of hydrogels was explored against bacterial biofilms common in implant-related infections, revealing concentration-dependent antibiofilm activity [313]. In another study, the ultrashort peptide hydrogels impregnated stable AgNPs were synthesized by self-assembly in water applicable in wound healing and assessed for antibacterial properties. The hydrogel was made from aliphatic amino acids, Ac-LIVAGK-NH2, as a matrix in which AgNPs were synthesized with silver nitrate by in situ UV irradiation method in a size-controlled manner. Using this strategy, the initial burst release within 48 h, followed by the sustained release of AgNPs, was observed for 14 days, necessary for antibacterial therapy. Inhibition of gram-negative (*E. coli* and *P. aeruginosa*) and positive (*S. aureus*) bacterial strains was proved by the disc diffusion method. The best results were for *P. aeruginosa* responsible in multidrug resistance. Owing to the low AgNPs content, sustained release, and biocompatibility on HDFa cells, the suggested hydrogels are proposed as a promising candidate in wound healing application [168].

## 5. Conclusions and Outlook

The present review has summarized supramolecular peptide hydrogels as self-assembled 3D structures, including their fundamental building blocks and formation together with their applications in various biomedical fields such as targeted drug delivery, wound healing, and 3D cell culture scaffolds. Owing to their inherent low toxicity, high biocompatibility, mechanical tunability, plus the capability to target small therapeutic drugs, some of these well-defined self-assembled peptide hydrogels have started to open their way into many clinical products for tissue engineering [314,315]. Table 2 has outlined some of the recent works performed regarding the use of self-assembled supramolecular peptide structures in clinical trials.

Although to date, self-assembled supramolecular peptide structures have shown outstanding results, there are still some issues to be tackled for these structures to enter any clinical trial phases. For instance, the in vivo toxicity of these nanostructures following systemic administration and the biodistribution of these peptide-based hydrogels are among the most important issues that should be addressed in more detail. A comprehensive evaluation of these two items will help researchers to design more biocompatible self-assembled peptide structures, which can be used for in vivo applications.

Additionally, more effort should be made to design peptide carriers with a more extended range of functions. To do so, the addition of polymers or nanomaterials to produce the pH, thermos, or magneto-responsive peptide hydrogels is of great importance. Furthermore, when used as a scaffold for SC transplantation, special attention should be devoted to the design of peptide hydrogels with the optimum mechanical properties as mechanical signals play an inevitable role in determining SC’s fate upon integration into a bio-scaffold [316].

The sterilization process of peptide-based materials is another concern that should be addressed, especially when used as injectable hydrogels. It is necessary to ensure hydrogel sterilization without compromising the functionality of the peptide hydrogels.

Lastly, when designing a peptide-based hydrogel for drug delivery and tissue engineering applications, the risk of recognition by the adaptive immune system and elimination should be brought under certain attention. Even though so many short-sequence peptides are not likely to provoke an immune response, longer sequences may evoke such immunological reactions [317]. Nevertheless, modification of hydrogel surface charge or using specific software to compare the hydrogel sequence with a database of known immune epitopes seems to be some useful suggested solutions to manage this issue [318].

**Table 2:** A list of the performed works in clinical trials for self-assembled supramolecular peptide structures.

Commercial Name	Target Organ	Peptide Structure	Application Field	Ref.
Curodont repair	Early occlusal caries	P11-4	Dentistry tissue regeneration	[314]
-	Initial buccal caries	P11-4	Dentistry tissue regeneration	[319]
-	Early buccal carious lesions	P11-4	Dentistry tissue regeneration	[320]
-	Surface caries lesions	P11-4	Dentistry tissue regeneration	[321]
-	Orthodontic treatment-induced carious lesions	P11-4	Dentistry tissue regeneration	[322]
PuraStat	Bleeding small blood vessels in cardiac surgery	RADA16	Hemostatic agent	[323]
SAPB-T45K	Skin lesion excision	T45K	Hemostatic agent+ Faster wound healing	[324]
Purastat	Endoscopic submucosal dissection	RADA16	Hemostatic agent	[325]
PuraMatrix	Peritoneal effusion	RADA16	Hemostatic agent+ Faster wound healing	[326]

## Figures and Tables

**Table 1:** The chemical properties of different types of amino acids [6,18].

Amino Acids	Properties
(1) Aliphatic hydrophobic i. Alanine (Ala, A) ii. Leucine (Leu, L) iii. Isoleucine (Ile, I) iv. Valine (Val, V) v. Methionine (Met, M)	Imparts a general hydrophobic environment
(2) Aromatic hydrophobic i. Phenylalanine (Phe, F) ii. Tyrosine (Tyr, Y) iii. Tryptophan (Trp, W)	Involved in π-π stacking, which is important for protein and peptide folding.
(3) Hydrophilic, uncharged: i. Asparagine (Asn, N) ii. Glutamine (Gln, Q) iii. Serine (Ser, S) iv. Threonine (Thr, T)	The -OH or -CONH groups are involved in hydrogen bonding interactions
(4) Positively charged (Basic) i. Histidine (His, H) ii. Arginine (Arg, R) iii. Lysine (Lys, K)	Involved in specific charge-charge interactions, by either exploiting attraction between oppositely charged groups or using repulsive forces between two equal charges.
(5) Negatively charged (Acidic) i. Glutamic acid (Glu, E) ii. Aspartic acid (Asp, D)	Involved in specific charge-charge interactions, by either exploiting attraction between oppositely charged groups or using repulsive forces between two equal charges.
(6) Specialized i. Cysteine (Cys, C) ii. Glycine (Gly, G) iii. Proline (Pro, P)	i. A target for chemical modification, either inter-peptide or between a peptide and other structures. ii. Responsible for a high degree of flexibility, by removing steric hindrances. iii. Responsible for a high degree of rigidity due to locked conformation.

## Data Availability

No new data were created.

## References

[1] Gavel P.K., Parmar H.S., Tripathi V., Kumar N., Biswas A., Das A.K. (2018). Investigations of anti-inflammatory activity of a peptide-based hydrogel using rat air pouch model. ACS Appl. Mater. Interfaces.

[2] Gavel P.K., Dev D., Parmar H.S., Bhasin S., Das A.K. (2018). Investigations of peptide-based biocompatible injectable shape-memory hydrogels: Differential biological effects on bacterial and human blood cells. ACS Appl. Mater. Interfaces.

[3] Du X., Zhou J., Shi J., Xu B. (2015). Supramolecular hydrogelators and hydrogels: From soft matter to molecular biomaterials. Chem. Rev..

[4] Acar H., Srivastava S., Chung E.J., Schnorenberg M.R., Barrett J.C., LaBelle J.L., Tirrell M. (2017). Self-assembling peptide-based building blocks in medical applications. Adv. Drug Deliv. Rev..

[5] Habibi N., Kamaly N., Memic A., Shafiee H. (2016). Self-assembled peptide-based nanostructures: Smart nanomaterials toward targeted drug delivery. Nano Today.

[6] Eskandari S., Guerin T., Toth I., Stephenson R.J. (2017). Recent advances in self-assembled peptides: Implications for targeted drug delivery and vaccine engineering. Adv. Drug Deliv. Rev..

[7] Vermonden T., Censi R., Hennink W.E. (2012). Hydrogels for protein delivery. Chem. Rev..

[8] Liu S., Zhao M., Zhou Y., Li L., Wang C., Yuan Y., Li L., Liao G., Bresette W., Chen Y. (2020). A self-assembling peptide hydrogel-based drug co-delivery platform to improve tissue repair after ischemia-reperfusion injury. Acta Biomater..

[9] Stephanopoulos N., Ortony J.H., Stupp S.I. (2013). Self-assembly for the synthesis of functional biomaterials. Acta Mater..

[10] Gavel P.K., Kumar N., Parmar H.S., Das A.K. (2020). Evaluation of a peptide-based coassembled nanofibrous and thixotropic hydrogel for dermal wound healing. ACS Appl. Bio Mater..

[11] Mondal S., Das S., Nandi A.K. (2020). A review on recent advances in polymer and peptide hydrogels. Soft Matter.

[12] Aldilla V.R., Nizalapur S., Martin A., Marjo C.E., Rich A., Yee E., Suwannakot P., Black D.S., Thordarson P., Kumar N. (2017). Design, synthesis, and characterisation of glyoxylamide-based short peptides as self-assembled gels. New J. Chem..

[13] Aviño F., Matheson A.B., Adams D.J., Clegg P.S. (2017). Stabilizing bubble and droplet interfaces using dipeptide hydrogels. Org. Biomol. Chem..

[14] Cardoso A.Z., Mears L.L., Cattoz B.N., Griffiths P.C., Schweins R., Adams D.J. (2016). Linking micellar structures to hydrogelation for salt-triggered dipeptide gelators. Soft Matter.

[15] Ferreira N., Ferreira L., Cardoso V., Boni F., Souza A., Gremião M. (2018). Recent advances in smart hydrogels for biomedical applications: From self-assembly to functional approaches. Eur. Polym. J..

[16] He C., Zhuang X., Tang Z., Tian H., Chen X. (2012). Stimuli-sensitive synthetic polypeptide-based materials for drug and gene delivery. Adv. Healthc. Mater..

[17] Zhang Z., Ai S., Yang Z., Li X. (2021). Peptide-based supramolecular hydrogels for local drug delivery. Adv. Drug Deliv. Rev..

[18] Ulijn R.V., Smith A.M. (2008). Designing peptide based nanomaterials. Chem. Soc. Rev..

[19] Koutsopoulos S. (2016). Self-assembling peptide nanofiber hydrogels in tissue engineering and regenerative medicine: Progress, design guidelines, and applications. J. Biomed. Mater. Res. Part A.

[20] Bairagi D., Biswas P., Basu K., Hazra S., Hermida-Merino D., Sinha D.K., Hamley I.W., Banerjee A. (2019). Self-assembling peptide-based hydrogel: Regulation of mechanical stiffness and thermal stability and 3D cell culture of fibroblasts. ACS Appl. Bio Mater..

[21] Curvello R., Raghuwanshi V.S., Garnier G. (2019). Engineering nanocellulose hydrogels for biomedical applications. Adv. Colloid Interface Sci..

[22] Worthington P., Pochan D.J., Langhans S.A. (2015). Peptide hydrogels–versatile matrices for 3D cell culture in cancer medicine. Front. Oncol..

[23] Sun L., Zheng C., Webster T.J. (2017). Self-assembled peptide nanomaterials for biomedical applications: Promises and pitfalls. Int. J. Nanomed..

[24] Van Vlierberghe S., Dubruel P., Schacht E. (2011). Biopolymer-based hydrogels as scaffolds for tissue engineering applications: A review. Biomacromolecules.

[25] Sun W., Xue B., Li Y., Qin M., Wu J., Lu K., Wu J., Cao Y., Jiang Q., Wang W. (2016). Polymer-supramolecular polymer double-network hydrogel. Adv. Funct. Mater..

[26] Fu K., Wu H., Su Z. (2021). Self-assembling peptide-based hydrogels: Fabrication, properties, and applications. Biotechnol. Adv..

[27] Liu X., Sun X., Liang G. (2021). Peptide-based supramolecular hydrogels for bioimaging applications. Biomater. Sci..

[28] Ahn W., Lee J.-H., Kim S.R., Lee J., Lee E.J. (2021). Designed protein-and peptide-based hydrogels for biomedical sciences. J. Mater. Chem. B.

[29] Liu J., Zhao X. (2011). Design of self-assembling peptides and their biomedical applications. Nanomedicine.

[30] Yu T., Greish K., McGill L.D., Ray A., Ghandehari H. (2012). Influence of geometry, porosity, and surface characteristics of silica nanoparticles on acute toxicity: Their vasculature effect and tolerance threshold. ACS Nano.

[31] Liang Y., He J., Guo B. (2021). Functional hydrogels as wound dressing to enhance wound healing. ACS Nano.

[32] Li J., Xing R., Bai S., Yan X. (2019). Recent advances of self-assembling peptide-based hydrogels for biomedical applications. Soft Matter.

[33] Sedighi M., Jalili H., Ranaei S.S.O., Amrane A. (2016). Potential health effects of enzymatic protein hydrolysates from Chlorella vulgaris. Appl. Food Biotechnol..

[34] Sadeghi S., Jalili H., Ranaei Siadat S., Sedighi M. (2018). Anticancer and antibacterial properties in peptide fractions from hydrolyzed spirulina protein. J. Agric. Sci. Technol..

[35] Darvish M., Jalili H., Ranaei-Siadat S.-O., Sedighi M. (2018). Potential cytotoxic effects of peptide fractions from Dunaliella salina protein hydrolyzed by gastric proteases. J. Aquat. Food Prod. Technol..

[36] Lodish A., Berk A., Zipursky S.L., Matsudaira P., Baltimore D., Darnell J. (2000). Molecular Cell Biology.

[37] Sedighi M., Mahmoudi Z., Ghasempour A., Shakibaie M., Ghasemi F., Akbari M., Abbaszadeh S., Mostafavi E., Santos H.A., Shahbazi M.-A. (2023). Nanostructured multifunctional stimuli-responsive glycopolypeptide-based copolymers for biomedical applications. J. Control. Release.

[38] De Santis E., Ryadnov M.G. (2015). Peptide self-assembly for nanomaterials: The old new kid on the block. Chem. Soc. Rev..

[39] Thiruvengadathan R., Korampally V., Ghosh A., Chanda N., Gangopadhyay K., Gangopadhyay S. (2013). Nanomaterial processing using self-assembly-bottom-up chemical and biological approaches. Rep. Prog. Phys..

[40] Dou X.Q., Feng C.L. (2017). Amino acids and peptide-based supramolecular hydrogels for three-dimensional cell culture. Adv. Mater..

[41] Lim J., Lin Q., Xue K., Loh X. (2019). Recent advances in supramolecular hydrogels for biomedical applications. Mater. Today Adv..

[42] Hilderbrand A.M., Ford E.M., Guo C., Sloppy J.D., Kloxin A.M. (2020). Hierarchically structured hydrogels utilizing multifunctional assembling peptides for 3D cell culture. Biomater. Sci..

[43] Fichman G., Gazit E. (2014). Self-assembly of short peptides to form hydrogels: Design of building blocks, physical properties and technological applications. Acta Biomater..

[44] Matson J.B., Zha R.H., Stupp S.I. (2011). Peptide self-assembly for crafting functional biological materials. Curr. Opin. Solid State Mater. Sci..

[45] Diaferia C., Netti F., Ghosh M., Sibillano T., Giannini C., Morelli G., Adler-Abramovich L., Accardo A. (2020). Bi-functional peptide-based 3D hydrogel-scaffolds. Soft Matter.

[46] Kumar P., Pillay V., Modi G., E Choonara Y., C du Toit L., Naidoo D. (2011). Self-assembling peptides: Implications for patenting in drug delivery and tissue engineering. Recent Pat. Drug Deliv. Formul..

[47] Pauling L., Corey R.B. (1951). The pleated sheet, a new layer configuration of polypeptide chains. Proc. Natl. Acad. Sci. USA.

[48] Zhang S., Holmes T., Lockshin C., Rich A. (1993). Spontaneous assembly of a self-complementary oligopeptide to form a stable macroscopic membrane. Proc. Natl. Acad. Sci. USA.

[49] Du E.Y., Ziaee F., Wang L., Nordon R.E., Thordarson P. (2020). The correlations between structure, rheology, and cell growth in peptide-based multicomponent hydrogels. Polym. J..

[50] Lee M., Jo Y.-B., Yoon J., Shin S. (2017). The Two Faces of Peptide Self-Assembly. Biophys. J..

[51] Wang Q., Zhang X., Zheng J., Liu D. (2014). Self-assembled peptide nanotubes as potential nanocarriers for drug delivery. RSC Adv..

[52] Varanko A., Saha S., Chilkoti A. (2020). Recent trends in protein and peptide-based biomaterials for advanced drug delivery. Adv. Drug Deliv. Rev..

[53] Lamm M.S., Rajagopal K., Schneider J.P., Pochan D.J. (2005). Laminated morphology of nontwisting β-sheet fibrils constructed via peptide self-assembly. J. Am. Chem. Soc..

[54] Worthington P., Langhans S., Pochan D. (2017). β-Hairpin peptide hydrogels for package delivery. Adv. Drug Deliv. Rev..

[55] Rughani R.V., Schneider J.P. (2008). Molecular design of β-hairpin peptides for material construction. MRS Bull..

[56] Delfi M., Sartorius R., Ashrafizadeh M., Sharifi E., Zhang Y., De Berardinis P., Zarrabi A., Varma R.S., Tay F.R., Smith B.R. (2021). Self-assembled peptide and protein nanostructures for anti-cancer therapy: Targeted delivery, stimuli-responsive devices and immunotherapy. Nano Today.

[57] Branco M.C., Pochan D.J., Wagner N.J., Schneider J.P. (2010). The effect of protein structure on their controlled release from an injectable peptide hydrogel. Biomaterials.

[58] Salem M., Rohani S., Gillies E.R. (2014). Curcumin, a promising anti-cancer therapeutic: A review of its chemical properties, bioactivity and approaches to cancer cell delivery. RSC Adv..

[59] Sinthuvanich C., Haines-Butterick L.A., Nagy K.J., Schneider J.P. (2012). Iterative design of peptide-based hydrogels and the effect of network electrostatics on primary chondrocyte behavior. Biomaterials.

[60] Yan C., Mackay M.E., Czymmek K., Nagarkar R.P., Schneider J.P., Pochan D.J. (2012). Injectable solid peptide hydrogel as a cell carrier: Effects of shear flow on hydrogels and cell payload. Langmuir.

[61] Salick D.A., Kretsinger J.K., Pochan D.J., Schneider J.P. (2007). Inherent antibacterial activity of a peptide-based β-hairpin hydrogel. J. Am. Chem. Soc..

[62] De Leon-Rodriguez L.M., Park Y.-E., Naot D., Musson D.S., Cornish J., Brimble M.A. (2020). Design, characterization and evaluation of β-hairpin peptide hydrogels as a support for osteoblast cell growth and bovine lactoferrin delivery. RSC Adv..

[63] Woolfson D.N., Ryadnov M.G. (2006). Peptide-based fibrous biomaterials: Some things old, new and borrowed. Curr. Opin. Chem. Biol..

[64] Wu Y., Collier J.H. (2017). α-Helical coiled-coil peptide materials for biomedical applications. Wiley Interdiscip. Rev. Nanomed. Nanobiotechnol..

[65] Woolfson D.N. (2010). Building fibrous biomaterials from α-helical and collagen-like coiled-coil peptides. Pept. Sci. Orig. Res. Biomol..

[66] Kopeček J. (2007). Hydrogel biomaterials: A smart future?. Biomaterials.

[67] Hill L.K., Meleties M., Katyal P., Xie X., Delgado-Fukushima E., Jihad T., Liu C.-F., O’Neill S., Tu R.S., Renfrew P.D. (2019). Thermoresponsive protein-engineered coiled-coil hydrogel for sustained small molecule release. Biomacromolecules.

[68] Lee S., Trinh T.H., Yoo M., Shin J., Lee H., Kim J., Hwang E., Lim Y.-B., Ryou C. (2019). Self-assembling peptides and their application in the treatment of diseases. Int. J. Mol. Sci..

[69] Li T., Lu X.-M., Zhang M.-R., Hu K., Li Z. (2022). Peptide-based nanomaterials: Self-assembly, properties and applications. Bioact. Mater..

[70] Reches M., Gazit E. (2003). Casting metal nanowires within discrete self-assembled peptide nanotubes. Science.

[71] Song Y., Challa S.R., Medforth C.J., Qiu Y., Watt R.K., Peña D., Miller J.E., van Swol F., Shelnutt J.A. (2004). Synthesis of peptide-nanotube platinum-nanoparticle composites. Chem. Commun..

[72] Yemini M., Reches M., Rishpon J., Gazit E. (2005). Novel electrochemical biosensing platform using self-assembled peptide nanotubes. Nano Lett..

[73] Yemini M., Reches M., Gazit E., Rishpon J. (2005). Peptide nanotube-modified electrodes for enzyme− biosensor applications. Anal. Chem..

[74] Mahler A., Reches M., Rechter M., Cohen S., Gazit E. (2006). Rigid, self-assembled hydrogel composed of a modified aromatic dipeptide. Adv. Mater..

[75] Reches M., Gazit E. (2005). Self-assembly of peptide nanotubes and amyloid-like structures by charged-termini-capped diphenylalanine peptide analogues. Isr. J. Chem..

[76] Reches M., Gazit E. (2004). Formation of closed-cage nanostructures by self-assembly of aromatic dipeptides. Nano Lett..

[77] Yang Z., Liang G., Xu B. (2006). Supramolecular hydrogels based on β-amino acid derivatives. Chem. Commun..

[78] Yang Z., Ho P.-L., Liang G., Chow K.H., Wang Q., Cao Y., Guo Z., Xu B. (2007). Using β-lactamase to trigger supramolecular hydrogelation. J. Am. Chem. Soc..

[79] Liu Z., Tang X., Feng F., Xu J., Wu C., Dai G., Yue W., Zhong W., Xu K. (2021). Molecular design of peptide amphiphiles for controlled self-assembly and drug release. J. Mater. Chem. B.

[80] Zhao C., Chen H., Wang F., Zhang X. (2021). Amphiphilic self-assembly peptides: Rational strategies to design and delivery for drugs in biomedical applications. Colloids Surf. B Biointerfaces.

[81] Palmer L.C., Stupp S.I. (2008). Molecular self-assembly into one-dimensional nanostructures. Acc. Chem. Res..

[82] Paramonov S.E., Jun H.W., Hartgerink J.D. (2006). Modulation of Peptide−Amphiphile Nanofibers via Phospholipid Inclusions. Biomacromolecules.

[83] Paramonov S.E., Jun H.-W., Hartgerink J.D. (2006). Self-assembly of peptide−amphiphile nanofibers: The roles of hydrogen bonding and amphiphilic packing. J. Am. Chem. Soc..

[84] Yanlian Y., Ulung K., Xiumei W., Horii A., Yokoi H., Shuguang Z. (2009). Designer self-assembling peptide nanomaterials. Nano Today.

[85] Li J., Wang J., Zhao Y., Zhou P., Carter J., Li Z., Waigh T.A., Lu J.R., Xu H. (2020). Surfactant-like peptides: From molecular design to controllable self-assembly with applications. Coord. Chem. Rev..

[86] Mello L.R., Aguiar R.B., Yamada R.Y., Moraes J.Z., Hamley I.W., Alves W.A., Reza M., Ruokolainen J., Silva E.R. (2020). Amphipathic design dictates self-assembly, cytotoxicity and cell uptake of arginine-rich surfactant-like peptides. J. Mater. Chem. B.

[87] Castelletto V., Seitsonen J., Ruokolainen J., Hamley I.W. (2021). Alpha helical surfactant-like peptides self-assemble into pH-dependent nanostructures. Soft Matter.

[88] Peng F., Chen Y., Liu J., Xing Z., Fan J., Zhang W., Qiu F. (2021). Facile design of gemini surfactant-like peptide for hydrophobic drug delivery and antimicrobial activity. J. Colloid Interface Sci..

[89] Fuhrhop J.-H., Wang T. (2004). Bolaamphiphiles. Chem. Rev..

[90] Nuraje N., Bai H., Su K. (2013). Bolaamphiphilic molecules: Assembly and applications. Prog. Polym. Sci..

[91] Qiu F., Tang C., Chen Y. (2018). Amyloid-like aggregation of designer bolaamphiphilic peptides: Effect of hydrophobic section and hydrophilic heads. J. Pept. Sci..

[92] Edwards-Gayle C.J., Castelletto V., Hamley I.W., Barrett G., Greco F., Hermida-Merino D., Rambo R.P., Seitsonen J., Ruokolainen J. (2019). Self-assembly, antimicrobial activity, and membrane interactions of arginine-capped peptide bola-amphiphiles. ACS Appl. Bio Mater..

[93] Tapeinou A., Matsoukas M.T., Simal C., Tselios T. (2015). Review cyclic peptides on a merry-go-round; towards drug design. Pept. Sci..

[94] Chapman R., Danial M., Koh M.L., Jolliffe K.A., Perrier S. (2012). Design and properties of functional nanotubes from the self-assembly of cyclic peptide templates. Chem. Soc. Rev..

[95] Montenegro J., Ghadiri M.R., Granja J.R. (2013). Ion channel models based on self-assembling cyclic peptide nanotubes. Acc. Chem. Res..

[96] Sun L., Fan Z., Wang Y., Huang Y., Schmidt M., Zhang M. (2015). Tunable synthesis of self-assembled cyclic peptide nanotubes and nanoparticles. Soft Matter.

[97] Ghadiri M.R., Granja J.R., Milligan R.A., McRee D.E., Khazanovich N. (1993). Self-assembling organic nanotubes based on a cyclic peptide architecture. Nature.

[98] Brea R.J., Reiriz C., Granja J.R. (2010). Towards functional bionanomaterials based on self-assembling cyclic peptide nanotubes. Chem. Soc. Rev..

[99] Fuertes A., Amorín M., Granja J.R. (2020). Versatile symport transporters based on cyclic peptide dimers. Chem. Commun..

[100] Li C., Chen X., Zhang F., He X., Fang G., Liu J., Wang S. (2017). Design of cyclic peptide based glucose receptors and their application in glucose sensing. Anal. Chem..

[101] Zhao Y., Leman L.J., Search D.J., Garcia R.A., Gordon D.A., Maryanoff B.E., Ghadiri M.R. (2017). Self-Assembling Cyclic d, l-α-Peptides as Modulators of Plasma HDL function. A supramolecular approach toward antiatherosclerotic agents. ACS Cent. Sci..

[102] Smith A.M., Williams R.J., Tang C., Coppo P., Collins R.F., Turner M.L., Saiani A., Ulijn R.V. (2008). Fmoc-diphenylalanine self assembles to a hydrogel via a novel architecture based on π–π interlocked β-sheets. Adv. Mater..

[103] Jayawarna V., Ali M., Jowitt T.A., Miller A.F., Saiani A., Gough J.E., Ulijn R.V. (2006). Nanostructured hydrogels for three-dimensional cell culture through self-assembly of fluorenylmethoxycarbonyl–dipeptides. Adv. Mater..

[104] Zhou M., Smith A.M., Das A.K., Hodson N.W., Collins R.F., Ulijn R.V., Gough J.E. (2009). Self-assembled peptide-based hydrogels as scaffolds for anchorage-dependent cells. Biomaterials.

[105] Afami M.E., El Karim I., About I., Krasnodembskaya A.D., Laverty G., Lundy F.T. (2021). Multicomponent Peptide Hydrogels as an Innovative Platform for Cell-Based Tissue Engineering in the Dental Pulp. Pharmaceutics.

[106] Huang R., Qi W., Feng L., Su R., He Z. (2011). Self-assembling peptide–polysaccharide hybrid hydrogel as a potential carrier for drug delivery. Soft Matter.

[107] Tang C., Smith A.M., Collins R.F., Ulijn R.V., Saiani A. (2009). Fmoc-diphenylalanine self-assembly mechanism induces apparent p K a shifts. Langmuir.

[108] Chu C.-W., Ravoo B.J. (2017). Hierarchical supramolecular hydrogels: Self-assembly by peptides and photo-controlled release via host–guest interaction. Chem. Commun..

[109] Chandrudu S., Simerska P., Toth I. (2013). Chemical methods for peptide and protein production. Molecules.

[110] Carpino L.A., Ghassemi S., Ionescu D., Ismail M., Sadat-Aalaee D., Truran G.A., Mansour E., Siwruk G.A., Eynon J.S., Morgan B. (2003). Rapid, continuous solution-phase peptide synthesis: Application to peptides of pharmaceutical interest. Org. Process Res. Dev..

[111] Nishiuchi Y., Inui T., Nishio H., Bódi J., Kimura T., Tsuji F.I., Sakakibara S. (1998). Chemical synthesis of the precursor molecule of the Aequorea green fluorescent protein, subsequent folding, and development of fluorescence. Proc. Natl. Acad. Sci. USA.

[112] Merryfield R. (1963). Solid phase peptide synthesis. J. Am. Chem. Soc.

[113] Alewood P., Alewood D., Miranda L., Love S., Meutermans W., Wilson D. (1997). [2] Rapid in situ neutralization protocols for Boc and Fmoc solid-phase chemistries. Methods Enzymol..

[114] Gudlur S., Sukthankar P., Gao J., Avila L.A., Hiromasa Y., Chen J., Iwamoto T., Tomich J.M. (2012). Peptide nanovesicles formed by the self-assembly of branched amphiphilic peptides. PLoS ONE.

[115] Natarajan P., Sukthankar P., Changstrom J., Holland C.S., Barry S., Hunter W.B., Sorensen C.M., Tomich J.M. (2018). Synthesis and characterization of multifunctional branched amphiphilic peptide bilayer conjugated gold nanoparticles. ACS Omega.

[116] Mauro N., Fiorica C., Varvarà P., Di Prima G., Giammona G. (2016). A facile way to build up branched high functional polyaminoacids with tunable physicochemical and biological properties. Eur. Polym. J..

[117] Kho J., Pham P.C., Kwon S., Huang A.Y., Rivers J.P., Wang H., Ecroyd H., Donald W.A., McAlpine S.R. (2021). De Novo Design, Synthesis, and Mechanistic Evaluation of Short Peptides That Mimic Heat Shock Protein 27 Activity. ACS Med. Chem. Lett..

[118] Teng Q., Wu H., Sun H., Liu Y., Wang H., Wang Z.-G. (2022). Switchable Enzyme-mimicking catalysts Self-Assembled from de novo designed peptides and DNA G-quadruplex/hemin complex. J. Colloid Interface Sci..

[119] Wang Y., Yang L., Wang M., Zhang J., Qi W., Su R., He Z. (2021). Bioinspired phosphatase-like mimic built from the self-assembly of de novo designed helical short peptides. ACS Catal..

[120] Eliyahu-Gross S., Bitton R. (2013). Environmentally responsive hydrogels with dynamically tunable properties as extracellular matrix mimetic. Rev. Chem. Eng..

[121] Wang M., Lv Y., Liu X., Qi W., Su R., He Z. (2016). Enhancing the activity of peptide-based artificial hydrolase with catalytic Ser/His/Asp triad and molecular imprinting. ACS Appl. Mater. Interfaces.

[122] Black K.A., Lin B.F., Wonder E.A., Desai S.S., Chung E.J., Ulery B.D., Katari R.S., Tirrell M.V. (2015). Biocompatibility and characterization of a peptide amphiphile hydrogel for applications in peripheral nerve regeneration. Tissue Eng. Part A.

[123] Tomatsu I., Peng K., Kros A. (2011). Photoresponsive hydrogels for biomedical applications. Adv. Drug Deliv. Rev..

[124] Muraoka T., Koh C.Y., Cui H., Stupp S.I. (2009). Light-triggered bioactivity in three dimensions. Angew. Chem..

[125] Szkolar L., Guilbaud J.B., Miller A.F., Gough J.E., Saiani A. (2014). Enzymatically triggered peptide hydrogels for 3D cell encapsulation and culture. J. Pept. Sci..

[126] Chronopoulou L., Lorenzoni S., Masci G., Dentini M., Togna A.R., Togna G., Bordi F., Palocci C. (2010). Lipase-supported synthesis of peptidic hydrogels. Soft Matter.

[127] Chronopoulou L., Togna A.R., Guarguaglini G., Masci G., Giammaruco F., Togna G.I., Palocci C. (2012). Self-assembling peptide hydrogels promote microglial cells proliferation and NGF production. Soft Matter.

[128] Isaacson K.J., Jensen M.M., Watanabe A.H., Green B.E., Correa M.A., Cappello J., Ghandehari H. (2018). Self-Assembly of Thermoresponsive Recombinant Silk-Elastinlike Nanogels. Macromol. Biosci..

[129] Cappello J., Crissman J., Crissman M., Ferrari F., Textor G., Wallis O., Whitledge J., Zhou X., Burman D., Aukerman L. (1998). In-situ self-assembling protein polymer gel systems for administration, delivery, and release of drugs. J. Control. Release.

[130] Dinerman A.A., Cappello J., Ghandehari H., Hoag S.W. (2002). Swelling behavior of a genetically engineered silk-elastinlike protein polymer hydrogel. Biomaterials.

[131] Huang W., Rollett A., Kaplan D.L. (2015). Silk-elastin-like protein biomaterials for the controlled delivery of therapeutics. Expert Opin. Drug Deliv..

[132] Megeed Z., Cappello J., Ghandehari H. (2002). Controlled release of plasmid DNA from a genetically engineered silk-elastinlike hydrogel. Pharm. Res..

[133] Greish K., Araki K., Li D., O’Malley Jr B.W., Dandu R., Frandsen J., Cappello J., Ghandehari H. (2009). Silk-elastinlike protein polymer hydrogels for localized adenoviral gene therapy of head and neck tumors. Biomacromolecules.

[134] Gustafson J., Greish K., Frandsen J., Cappello J., Ghandehari H. (2009). Silk-elastinlike recombinant polymers for gene therapy of head and neck cancer: From molecular definition to controlled gene expression. J. Control. Release.

[135] Gustafson J.A., Price R.A., Greish K., Cappello J., Ghandehari H. (2010). Silk-elastin-like hydrogel improves the safety of adenovirus-mediated gene-directed enzyme− Prodrug therapy. Mol. Pharm..

[136] Price R., Gustafson J., Greish K., Cappello J., McGill L., Ghandehari H. (2012). Comparison of silk-elastinlike protein polymer hydrogel and poloxamer in matrix-mediated gene delivery. Int. J. Pharm..

[137] Nagarsekar A., Crissman J., Crissman M., Ferrari F., Cappello J., Ghandehari H. (2002). Genetic synthesis and characterization of pH-and temperature-sensitive silk-elastinlike protein block copolymers. J. Biomed. Mater. Res..

[138] Nagarsekar A., Crissman J., Crissman M., Ferrari F., Cappello J., Ghandehari H. (2003). Genetic engineering of stimuli-sensitive silkelastin-like protein block copolymers. Biomacromolecules.

[139] Machado R., Da Costa A., Sencadas V., Garcia-Arévalo C., Costa C.M., Padrao J., Gomes A., Lanceros-Méndez S., Rodríguez-Cabello J.C., Casal M. (2013). Electrospun silk-elastin-like fibre mats for tissue engineering applications. Biomed. Mater..

[140] Narayan O.P., Mu X., Hasturk O., Kaplan D.L. (2021). Dynamically tunable light responsive silk-elastin-like proteins. Acta Biomater..

[141] Petros R.A., DeSimone J.M. (2010). Strategies in the design of nanoparticles for therapeutic applications. Nat. Rev. Drug Discov..

[142] Byrne J.D., Betancourt T., Brannon-Peppas L. (2008). Active targeting schemes for nanoparticle systems in cancer therapeutics. Adv. Drug Deliv. Rev..

[143] Bazak R., Houri M., El Achy S., Hussein W., Refaat T. (2014). Passive targeting of nanoparticles to cancer: A comprehensive review of the literature. Mol. Clin. Oncol..

[144] Sedighi M., Rahimi F., Shahbazi M.-A., Rezayan A.H., Kettiger H., Einfalt T., Huwyler J., Witzigmann D. (2019). Controlled tyrosine kinase inhibitor delivery to liver cancer cells by gate-capped mesoporous silica nanoparticles. ACS Appl. Bio Mater..

[145] Franks S.J., Firipis K., Ferreira R., Hannan K.M., Williams R.J., Hannan R.D., Nisbet D.R. (2020). Harnessing the self-assembly of peptides for the targeted delivery of anti-cancer agents. Mater. Horiz..

[146] Han K., Ma Z., Han H. (2018). Functional peptide-based nanoparticles for photodynamic therapy. J. Mater. Chem. B.

[147] Cai Y., Zheng C., Xiong F., Ran W., Zhai Y., Zhu H.H., Wang H., Li Y., Zhang P. (2021). Recent Progress in the Design and Application of Supramolecular Peptide Hydrogels in Cancer Therapy. Adv. Healthc. Mater..

[148] Han S.S., Li Z.Y., Zhu J.Y., Han K., Zeng Z.Y., Hong W., Li W.X., Jia H.Z., Liu Y., Zhuo R.X. (2015). Dual-pH sensitive charge-reversal polypeptide micelles for tumor-triggered targeting uptake and nuclear drug delivery. Small.

[149] Liu J., Liu J., Xu H., Zhang Y., Chu L., Liu Q., Song N., Yang C. (2014). Novel tumor-targeting, self-assembling peptide nanofiber as a carrier for effective curcumin delivery. Int. J. Nanomed..

[150] Xu X., Li Y., Li H., Liu R., Sheng M., He B., Gu Z. (2014). Smart Nanovehicles Based on pH-Triggered Disassembly of Supramolecular Peptide-Amphiphiles for Efficient Intracellular Drug Delivery. Small.

[151] Yazdi M.K., Zarrintaj P., Ghavami M., Alizadeh R., Saeb M.R., Mozafari M. (2020). 7—Protein and peptide-based delivery systems. Nanoengineered Biomaterials for Advanced Drug Delivery.

[152] Raza F., Zhu Y., Chen L., You X., Zhang J., Khan A., Khan M.W., Hasnat M., Zafar H., Wu J. (2019). Paclitaxel-loaded pH responsive hydrogel based on self-assembled peptides for tumor targeting. Biomater. Sci..

[153] Abbas M., Zou Q., Li S., Yan X. (2017). Self-assembled peptide-and protein-based nanomaterials for antitumor photodynamic and photothermal therapy. Adv. Mater..

[154] Li S.-Y., Cheng H., Qiu W.-X., Liu L.-H., Chen S., Hu Y., Xie B.-R., Li B., Zhang X.-Z. (2015). Protease-activable cell-penetrating peptide–protoporphyrin conjugate for targeted photodynamic therapy in vivo. ACS Appl. Mater. Interfaces.

[155] Han K., Wang S.-B., Lei Q., Zhu J.-Y., Zhang X.-Z. (2015). Ratiometric biosensor for aggregation-induced emission-guided precise photodynamic therapy. ACS Nano.

[156] Zhao D.-H., Yang J., Xia R.-X., Yao M.-H., Jin R.-M., Zhao Y.-D., Liu B. (2018). High quantum yield Ag_2_S quantum dot@ polypeptide-engineered hybrid nanogels for targeted second near-infrared fluorescence/photoacoustic imaging and photothermal therapy. Chem. Commun..

[157] Lakshmanan A., Zhang S., Hauser C.A. (2012). Short self-assembling peptides as building blocks for modern nanodevices. Trends Biotechnol..

[158] Yan X., Zhu P., Li J. (2010). Self-assembly and application of diphenylalanine-based nanostructures. Chem. Soc. Rev..

[159] Pigliacelli C., Sanjeeva K.B., Nonappa, Pizzi A., Gori A., Bombelli F.B., Metrangolo P. (2019). In situ generation of chiroptically-active gold-peptide superstructures promoted by iodination. ACS Nano.

[160] Krajina B.A., Proctor A.C., Schoen A.P., Spakowitz A.J., Heilshorn S.C. (2018). Biotemplated synthesis of inorganic materials: An emerging paradigm for nanomaterial synthesis inspired by nature. Prog. Mater. Sci..

[161] Meier C., Lifincev I., Welland M.E. (2015). Conducting core–shell nanowires by amyloid nanofiber templated polymerization. Biomacromolecules.

[162] Wang C., Sun Y., Wang J., Xu H., Lu J.R. (2015). Copper (II)-Mediated Self-Assembly of Hairpin Peptides and Templated Synthesis of CuS Nanowires. Chem.–Asian J..

[163] Ahmed S., Mondal J.H., Behera N., Das D. (2013). Self-assembly of peptide-amphiphile forming helical nanofibers and in situ template synthesis of uniform mesoporous single wall silica nanotubes. Langmuir.

[164] Acharya G., Shin C.S., McDermott M., Mishra H., Park H., Kwon I.C., Park K. (2010). The hydrogel template method for fabrication of homogeneous nano/microparticles. J. Control. Release.

[165] Adhikari B., Banerjee A. (2010). Short-Peptide-Based Hydrogel: A Template for the In Situ Synthesis of Fluorescent Silver Nanoclusters by Using Sunlight. Chem.–Eur. J..

[166] Ni M., Zhuo S. (2019). Applications of self-assembling ultrashort peptides in bionanotechnology. RSC Adv..

[167] Jain R., Khandelwal G., Roy S. (2019). Unraveling the Design Rules in Ultrashort Amyloid-Based Peptide Assemblies toward Shape-Controlled Synthesis of Gold Nanoparticles. Langmuir.

[168] Reithofer M.R., Lakshmanan A., Ping A.T., Chin J.M., Hauser C.A. (2014). In situ synthesis of size-controlled, stable silver nanoparticles within ultrashort peptide hydrogels and their anti-bacterial properties. Biomaterials.

[169] Zhang Z., Xu Y., Zhang Y., Ma B., Ma Z., Han H. (2022). Antifouling and sensitive biosensor based on multifunctional peptide and urease@ ZIFs for metal matrix protease-7. Sens. Actuators B Chem..

[170] Kim C.-J., Park J.-E., Hu X., Albert S.K., Park S.-J. (2020). Peptide-Driven shape control of low-dimensional DNA nanostructures. ACS Nano.

[171] Mitragotri S., Lahann J. (2009). Physical approaches to biomaterial design. Nat. Mater..

[172] Ravi M., Paramesh V., Kaviya S., Anuradha E., Solomon F.P. (2015). 3D cell culture systems: Advantages and applications. J. Cell. Physiol..

[173] Das A.K., Gavel P.K. (2020). Low molecular weight self-assembling peptide-based materials for cell culture, antimicrobial, anti-inflammatory, wound healing, anticancer, drug delivery, bioimaging and 3D bioprinting applications. Soft Matter.

[174] Tibbitt M.W., Anseth K.S. (2009). Hydrogels as extracellular matrix mimics for 3D cell culture. Biotechnol. Bioeng..

[175] Huang H., Ding Y., Sun X.S., Nguyen T.A. (2013). Peptide hydrogelation and cell encapsulation for 3D culture of MCF-7 breast cancer cells. PLoS ONE.

[176] Huang H., Herrera A.I., Luo Z., Prakash O., Sun X.S. (2012). Structural transformation and physical properties of a hydrogel-forming peptide studied by NMR, transmission electron microscopy, and dynamic rheometer. Biophys. J..

[177] Mallia V.A., George M., Blair D.L., Weiss R.G. (2009). Robust organogels from nitrogen-containing derivatives of (R)-12-hydroxystearic acid as gelators: Comparisons with gels from stearic acid derivatives. Langmuir.

[178] Jacob R.S., Ghosh D., Singh P.K., Basu S.K., Jha N.N., Das S., Sukul P.K., Patil S., Sathaye S., Kumar A. (2015). Self healing hydrogels composed of amyloid nano fibrils for cell culture and stem cell differentiation. Biomaterials.

[179] Hull C.W. (2015). The birth of 3D printing. Res.-Technol. Manag..

[180] Gungor-Ozkerim P.S., Inci I., Zhang Y.S., Khademhosseini A., Dokmeci M.R. (2018). Bioinks for 3D bioprinting: An overview. Biomater. Sci..

[181] Murphy S.V., Atala A. (2014). 3D bioprinting of tissues and organs. Nat. Biotechnol..

[182] Chia H.N., Wu B.M. (2015). Recent advances in 3D printing of biomaterials. J. Biol. Eng..

[183] Melchels F.P., Domingos M.A., Klein T.J., Malda J., Bartolo P.J., Hutmacher D.W. (2012). Additive manufacturing of tissues and organs. Prog. Polym. Sci..

[184] Jungst T., Smolan W., Schacht K., Scheibel T., Groll J. (2016). Strategies and molecular design criteria for 3D printable hydrogels. Chem. Rev..

[185] Loo Y., Hauser C.A. (2015). Bioprinting synthetic self-assembling peptide hydrogels for biomedical applications. Biomed. Mater..

[186] Jammalamadaka U., Tappa K. (2018). Recent advances in biomaterials for 3D printing and tissue engineering. J. Funct. Biomater..

[187] Loo Y., Lakshmanan A., Ni M., Toh L.L., Wang S., Hauser C.A. (2015). Peptide bioink: Self-assembling nanofibrous scaffolds for three-dimensional organotypic cultures. Nano Lett..

[188] Raphael B., Khalil T., Workman V.L., Smith A., Brown C.P., Streuli C., Saiani A., Domingos M. (2017). 3D cell bioprinting of self-assembling peptide-based hydrogels. Mater. Lett..

[189] Graham A.D., Olof S.N., Burke M.J., Armstrong J.P., Mikhailova E.A., Nicholson J.G., Box S.J., Szele F.G., Perriman A.W., Bayley H. (2017). High-resolution patterned cellular constructs by droplet-based 3D printing. Sci. Rep..

[190] Byrne M.E., Park K., Peppas N.A. (2002). Molecular imprinting within hydrogels. Adv. Drug Deliv. Rev..

[191] Neves M.I., Wechsler M.E., Gomes M.E., Reis R.L., Granja P.L., Peppas N.A. (2017). Molecularly imprinted intelligent scaffolds for tissue engineering applications. Tissue Eng. Part B Rev..

[192] Byrne M.E., Salian V. (2008). Molecular imprinting within hydrogels II: Progress and analysis of the field. Int. J. Pharm..

[193] Matsumoto K., Kawamura A., Miyata T. (2017). Conformationally regulated molecular binding and release of molecularly imprinted polypeptide hydrogels that undergo helix–coil transition. Macromolecules.

[194] Wu X.L., Kim J.H., Koo H., Bae S.M., Shin H., Kim M.S., Lee B.H., Park R.W., Kim I.S., Choi K. (2010). Tumor-targeting peptide conjugated pH-responsive micelles as a potential drug carrier for cancer therapy. Bioconjug. Chem..

[195] Zhou M., Zou X., Cheng K., Zhong S., Su Y., Wu T., Tao Y., Cong L., Yan B., Jiang Y. (2022). The role of cell-penetrating peptides in potential anti-cancer therapy. Clin. Transl. Med..

[196] Sarangthem V., Kim Y., Singh T.D., Seo B.Y., Cheon S.H., Lee Y.J., Lee B.H., Park R.W. (2016). Multivalent Targeting Based Delivery of Therapeutic Peptide using AP1-ELP Carrier for Effective Cancer Therapy. Theranostics.

[197] Samec T., Boulos J., Gilmore S., Hazelton A., Alexander-Bryant A. (2022). Peptide-based delivery of therapeutics in cancer treatment. Mater Today Bio.

[198] Qi Y., Min H., Mujeeb A., Zhang Y., Han X., Zhao X., Anderson G.J., Zhao Y., Nie G. (2018). Injectable hexapeptide hydrogel for localized chemotherapy prevents breast cancer recurrence. ACS Appl. Mater. Interfaces.

[199] Kalafatovic D., Nobis M., Son J., Anderson K.I., Ulijn R.V. (2016). MMP-9 triggered self-assembly of doxorubicin nanofiber depots halts tumor growth. Biomaterials.

[200] Yuan C.S., Deng Z.W., Qin D., Mu Y.Z., Chen X.G., Liu Y. (2021). Hypoxia-modulatory nanomaterials to relieve tumor hypoxic microenvironment and enhance immunotherapy: Where do we stand?. Acta Biomater..

[201] Volovat S.R., Negru S., Stolniceanu C.R., Volovat C., Lungulescu C., Scripcariu D., Cobzeanu B.M., Stefanescu C., Grigorescu C., Augustin I. (2021). Nanomedicine to modulate immunotherapy in cutaneous melanoma (Review). Exp. Ther. Med..

[202] Abudula T., Bhatt K., Eggermont L.J., O’Hare N., Memic A., Bencherif S.A. (2020). Supramolecular Self-Assembled Peptide-Based Vaccines: Current State and Future Perspectives. Front. Chem..

[203] Li L., Ma B., Wang W. (2020). Peptide-Based Nanomaterials for Tumor Immunotherapy. Molecules.

[204] Li S., Zhang W., Xue H., Xing R., Yan X. (2020). Tumor microenvironment-oriented adaptive nanodrugs based on peptide self-assembly. Chem. Sci..

[205] Rizvi S.F.A., Mu S., Zhao C., Zhang H. (2022). Fabrication of self-assembled peptide nanoparticles for in vitro assessment of cell apoptosis pathway and in vivo therapeutic efficacy. Mikrochim. Acta.

[206] Moon Y., Shim M.K., Choi J., Yang S., Kim J., Yun W.S., Cho H., Park J.Y., Kim Y., Seong J.K. (2022). Anti-PD-L1 peptide-conjugated prodrug nanoparticles for targeted cancer immunotherapy combining PD-L1 blockade with immunogenic cell death. Theranostics.

[207] Wei Z., Yi Y., Luo Z., Gong X., Jiang Y., Hou D., Zhang L., Liu Z., Wang M., Wang J. (2022). Selenopeptide Nanomedicine Activates Natural Killer Cells for Enhanced Tumor Chemoimmunotherapy. Adv. Mater..

[208] Leach D.G., Dharmaraj N., Piotrowski S.L., Lopez-Silva T.L., Lei Y.L., Sikora A.G., Young S., Hartgerink J.D. (2018). STINGel: Controlled release of a cyclic dinucleotide for enhanced cancer immunotherapy. Biomaterials.

[209] Cheng K., Ding Y., Zhao Y., Ye S., Zhao X., Zhang Y., Ji T., Wu H., Wang B., Anderson G.J. (2018). Sequentially Responsive Therapeutic Peptide Assembling Nanoparticles for Dual-Targeted Cancer Immunotherapy. Nano Lett..

[210] Li M., Yang Y., Wei J., Cun X., Lu Z., Qiu Y., Zhang Z., He Q. (2018). Enhanced chemo-immunotherapy against melanoma by inhibition of cholesterol esterification in CD8(+) T cells. Nanomedicine.

[211] Wang T., Wang D., Yu H., Feng B., Zhou F., Zhang H., Zhou L., Jiao S., Li Y. (2018). A cancer vaccine-mediated postoperative immunotherapy for recurrent and metastatic tumors. Nat. Commun..

[212] Mehrotra P. (2016). Biosensors and their applications—A review. J. Oral Biol. Craniofac. Res..

[213] Jayawarna V., Smith A., Gough J., Ulijn R. (2007). Three-dimensional cell culture of chondrocytes on modified di-phenylalanine scaffolds. Biochem. Soc. Trans..

[214] Kim J.H., Lim S.Y., Nam D.H., Ryu J., Ku S.H., Park C.B. (2011). Self-assembled, photoluminescent peptide hydrogel as a versatile platform for enzyme-based optical biosensors. Biosens. Bioelectron..

[215] Fusco G., Chronopoulou L., Galantini L., Zerillo A., Rasik Z.M., Antiochia R., Favero G., D’Annibale A., Palocci C., Mazzei F. (2018). Evaluation of novel Fmoc-tripeptide based hydrogels as immobilization supports for electrochemical biosensors. Microchem. J..

[216] Lian M., Chen X., Lu Y., Yang W. (2016). Self-assembled peptide hydrogel as a smart biointerface for enzyme-based electrochemical biosensing and cell monitoring. ACS Appl. Mater. Interfaces.

[217] King P.J., Saiani A., Bichenkova E.V., Miller A.F. (2016). A de novo self-assembling peptide hydrogel biosensor with covalently immobilised DNA-recognising motifs. Chem. Commun..

[218] Lian M., Xu L., Zhu X., Chen X., Yang W., Wang T. (2017). Seamless signal transduction from three-dimensional cultured cells to a superoxide anions biosensor via in situ self-assembly of dipeptide hydrogel. Anal. Chem..

[219] Gong Y., Chen X., Lu Y., Yang W. (2015). Self-assembled dipeptide–gold nanoparticle hybrid spheres for highly sensitive amperometric hydrogen peroxide biosensors. Biosens. Bioelectron..

[220] Mehwish N., Dou X., Zhao Y., Feng C.-L. (2019). Supramolecular fluorescent hydrogelators as bio-imaging probes. Mater. Horiz..

[221] Kim B.J., Xu B. (2020). Enzyme-instructed self-assembly for cancer therapy and imaging. Bioconjug. Chem..

[222] Ji W., Liu G., Wang F., Zhu Z., Feng C. (2016). Galactose-decorated light-responsive hydrogelator precursors for selectively killing cancer cells. Chem. Commun..

[223] Li R., Horgan C.C., Long B., Rodriguez A.L., Mather L., Barrow C.J., Nisbet D.R., Williams R.J. (2015). Tuning the mechanical and morphological properties of self-assembled peptide hydrogels via control over the gelation mechanism through regulation of ionic strength and the rate of pH change. RSC Adv..

[224] Odriozola I., Loinaz I., Pomposo J.A., Grande H.J. (2007). Gold–glutathione supramolecular hydrogels. J. Mater. Chem..

[225] Pappas C.G., Frederix P.W., Mutasa T., Fleming S., Abul-Haija Y.M., Kelly S.M., Gachagan A., Kalafatovic D., Trevino J., Ulijn R.V. (2015). Alignment of nanostructured tripeptide gels by directional ultrasonication. Chem. Commun..

[226] Singh A., Joseph J.P., Gupta D., Sarkar I., Pal A. (2018). Pathway driven self-assembly and living supramolecular polymerization in an amyloid-inspired peptide amphiphile. Chem. Commun..

[227] Wojciechowski J.P., Martin A.D., Thordarson P. (2018). Kinetically controlled lifetimes in redox-responsive transient supramolecular hydrogels. J. Am. Chem. Soc..

[228] Yoshii T., Onogi S., Shigemitsu H., Hamachi I. (2015). Chemically reactive supramolecular hydrogel coupled with a signal amplification system for enhanced analyte sensitivity. J. Am. Chem. Soc..

[229] Mayr J., Saldías C., Díaz D.D. (2018). Release of small bioactive molecules from physical gels. Chem. Soc. Rev..

[230] Wang X., Wang J., Guo L., Wang X., Chen H., Wang X., Liu J., Tredget E.E., Wu Y. (2016). Self-assembling peptide hydrogel scaffolds support stem cell-based hair follicle regeneration. Nanomed. Nanotechnol. Biol. Med..

[231] Yin L., Xu S., Feng Z., Deng H., Zhang J., Gao H., Deng L., Tang H., Dong A. (2017). Supramolecular hydrogel based on high-solid-content mPECT nanoparticles and cyclodextrins for local and sustained drug delivery. Biomater. Sci..

[232] Gao Y., Shi J., Yuan D., Xu B. (2012). Imaging enzyme-triggered self-assembly of small molecules inside live cells. Nat. Commun..

[233] Fan Z., Sun L., Huang Y., Wang Y., Zhang M. (2016). Bioinspired fluorescent dipeptide nanoparticles for targeted cancer cell imaging and real-time monitoring of drug release. Nat. Nanotechnol..

[234] Gan Z., Xu H. (2017). Photoluminescence of diphenylalanine peptide nano/microstructures: From mechanisms to applications. Macromol. Rapid Commun..

[235] Lock L.L., Li Y., Mao X., Chen H., Staedtke V., Bai R., Ma W., Lin R., Li Y., Liu G. (2017). One-component supramolecular filament hydrogels as theranostic label-free magnetic resonance imaging agents. ACS Nano.

[236] Wang L.V. (2009). Multiscale photoacoustic microscopy and computed tomography. Nat. Photonics.

[237] Huang P., Gao Y., Lin J., Hu H., Liao H.-S., Yan X., Tang Y., Jin A., Song J., Niu G. (2015). Tumor-specific formation of enzyme-instructed supramolecular self-assemblies as cancer theranostics. ACS Nano.

[238] Gui S., Huang Y., Hu F., Jin Y., Zhang G., Zhang D., Zhao R. (2018). Bioinspired peptide for imaging Hg2+ distribution in living cells and zebrafish based on coordination-mediated supramolecular assembling. Anal. Chem..

[239] Huang Z., Yao Q., Chen J., Gao Y. (2018). Redox supramolecular self-assemblies nonlinearly enhance fluorescence to identify cancer cells. Chem. Commun..

[240] Zhao X.X., Li L.L., Zhao Y., An H.W., Cai Q., Lang J.Y., Han X.X., Peng B., Fei Y., Liu H. (2019). In Situ Self-Assembled Nanofibers Precisely Target Cancer-Associated Fibroblasts for Improved Tumor Imaging. Angew. Chem..

[241] An H.-W., Hou D., Zheng R., Wang M.-D., Zeng X.-Z., Xiao W.-Y., Yan T.-D., Wang J.-Q., Zhao C.-H., Cheng L.-M. (2020). A near-infrared peptide probe with tumor-specific excretion-retarded effect for image-guided surgery of renal cell carcinoma. ACS Nano.

[242] Zheng Z., Tang A., Guan Y., Chen L., Wang F., Chen P., Wang W., Luo Y., Tian Y., Liang G. (2016). Nanocomputed tomography imaging of bacterial alkaline phosphatase activity with an iodinated hydrogelator. Anal. Chem..

[243] Morris O., Elsawy M., Fairclough M., Williams K.J., McMahon A., Grigg J., Forster D., Miller A., Saiani A., Prenant C. (2017). In vivo characterisation of a therapeutically relevant self-assembling 18F-labelled β-sheet forming peptide and its hydrogel using positron emission tomography. J. Label. Compd. Radiopharm..

[244] Oyen E., Martin C., Caveliers V., Madder A., Van Mele B., Hoogenboom R., Hernot S., Ballet S. (2017). In vivo imaging of the stability and sustained cargo release of an injectable amphipathic peptide-based hydrogel. Biomacromolecules.

[245] Wang X., Yu X., Wang X., Qi M., Pan J., Wang Q. (2019). One-step nanosurface self-assembly of d-peptides renders bubble-free ultrasound theranostics. Nano Lett..

[246] Li H., Meade T.J. (2019). Molecular magnetic resonance imaging with Gd (III)-based contrast agents: Challenges and key advances. J. Am. Chem. Soc..

[247] Lusic H., Grinstaff M.W. (2013). X-ray-computed tomography contrast agents. Chem. Rev..

[248] Weber J., Beard P.C., Bohndiek S.E. (2016). Contrast agents for molecular photoacoustic imaging. Nat. Methods.

[249] Wu X., Shi W., Li X., Ma H. (2019). Recognition moieties of small molecular fluorescent probes for bioimaging of enzymes. Acc. Chem. Res..

[250] Song Y., Huang Z., Xu J., Ren D., Wang Y., Zheng X., Shen Y., Wang L., Gao H., Hou J. (2014). Multimodal SPION-CREKA peptide based agents for molecular imaging of microthrombus in a rat myocardial ischemia-reperfusion model. Biomaterials.

[251] Dong L., Qian J., Hai Z., Xu J., Du W., Zhong K., Liang G. (2017). Alkaline phosphatase-instructed self-assembly of gadolinium nanofibers for enhanced T2-weighted magnetic resonance imaging of tumor. Anal. Chem..

[252] Lee S., Xie J., Chen X. (2010). Peptides and peptide hormones for molecular imaging and disease diagnosis. Chem. Rev..

[253] Nie L., Chen X. (2014). Structural and functional photoacoustic molecular tomography aided by emerging contrast agents. Chem. Soc. Rev..

[254] Khurana V., Kwatra D., Shah S., Mandal A., Mitra A.K. (2017). Emerging nanotechnology for stem cell therapy. Emerging Nanotechnologies for Diagnostics, Drug Delivery and Medical Devices.

[255] Liu Z., Tang M., Zhao J., Chai R., Kang J. (2018). Looking into the future: Toward advanced 3D biomaterials for stem-Cell-based regenerative medicine. Adv. Mater..

[256] Elbuluk A., Einhorn T.A., Iorio R. (2017). A comprehensive review of stem-cell therapy. JBJS Rev..

[257] Oliveira J.M., Carvalho L., Silva-Correia J., Vieira S., Majchrzak M., Lukomska B., Stanaszek L., Strymecka P., Malysz-Cymborska I., Golubczyk D. (2018). Hydrogel-based scaffolds to support intrathecal stem cell transplantation as a gateway to the spinal cord: Clinical needs, biomaterials, and imaging technologies. NPJ Regen. Med..

[258] Du X., Zhou J., Guvench O., Sangiorgi F.O., Li X., Zhou N., Xu B. (2014). Supramolecular assemblies of a conjugate of nucleobase, amino acids, and saccharide act as agonists for proliferation of embryonic stem cells and development of zygotes. Bioconjug. Chem..

[259] Cheng T.-Y., Chen M.-H., Chang W.-H., Huang M.-Y., Wang T.-W. (2013). Neural stem cells encapsulated in a functionalized self-assembling peptide hydrogel for brain tissue engineering. Biomaterials.

[260] Koutsopoulos S., Zhang S. (2013). Long-term three-dimensional neural tissue cultures in functionalized self-assembling peptide hydrogels, matrigel and collagen I. Acta Biomater..

[261] Qiao S., Liu Y., Han F., Guo M., Hou X., Ye K., Deng S., Shen Y., Zhao Y., Wei H. (2018). An intelligent neural stem cell delivery system for neurodegenerative diseases treatment. Adv. Healthc. Mater..

[262] Li H., Johnson N., Usas A., Lu A., Poddar M., Wang Y., Huard J. (2013). Stem Cells Transl. Med.

[263] Sehgal R.R., Banerjee R. (2013). IKVAV-functionalized self-assembling peptide hydrogel for improved neural stem cell transplantation. Nanomedicine.

[264] Li Q., Chow K.L., Chau Y. (2014). Three-dimensional self-assembling peptide matrix enhances the formation of embryoid bodies and their neuronal differentiation. J. Biomed. Mater. Res. Part A.

[265] Sun W., Incitti T., Migliaresi C., Quattrone A., Casarosa S., Motta A. (2017). Viability and neuronal differentiation of neural stem cells encapsulated in silk fibroin hydrogel functionalized with an IKVAV peptide. J. Tissue Eng. Regen. Med..

[266] Rodriguez A., Bruggeman K., Wang Y., Wang T., Williams R., Parish C., Nisbet D. (2018). Using minimalist self-assembling peptides as hierarchical scaffolds to stabilise growth factors and promote stem cell integration in the injured brain. J. Tissue Eng. Regen. Med..

[267] Sallouh M., Jarocki M., Sallouh O., Degen P., Faissner A., Weberskirch R. (2017). The synergistic effect of cationic moieties and GRGDSF-peptides in hydrogels on neural stem cell behavior. Macromol. Biosci..

[268] Liu Y., Ye H., Satkunendrarajah K., Yao G.S., Bayon Y., Fehlings M.G. (2013). A self-assembling peptide reduces glial scarring, attenuates post-traumatic inflammation and promotes neurological recovery following spinal cord injury. Acta Biomater..

[269] Iwasaki M., Wilcox J.T., Nishimura Y., Zweckberger K., Suzuki H., Wang J., Liu Y., Karadimas S.K., Fehlings M.G. (2014). Synergistic effects of self-assembling peptide and neural stem/progenitor cells to promote tissue repair and forelimb functional recovery in cervical spinal cord injury. Biomaterials.

[270] Shan W., Wang B., Xu Y., Li X., Li X., Wang H., Lin Y., Tie R., Zhao Q., Wang J. (2020). Generation of hematopoietic cells from mouse pluripotent stem cells in a 3D culture system of self-assembling peptide hydrogel. J. Cell. Physiol..

[271] Yang R., Yang S., Zhao J., Hu X., Chen X., Wang J., Xie J., Xiong K. (2020). Progress in studies of epidermal stem cells and their application in skin tissue engineering. Stem Cell Res. Ther..

[272] Khayambashi P., Iyer J., Pillai S., Upadhyay A., Zhang Y., Tran S.D. (2021). Hydrogel Encapsulation of Mesenchymal Stem Cells and Their Derived Exosomes for Tissue Engineering. Int. J. Mol. Sci..

[273] Patel M., Moon H.J., Ko D.Y., Jeong B. (2016). Composite system of graphene oxide and polypeptide thermogel as an injecTable 3D scaffold for adipogenic differentiation of tonsil-derived mesenchymal stem cells. ACS Appl. Mater. Interfaces.

[274] Hogrebe N.J., Gooch K.J. (2016). Direct influence of culture dimensionality on human mesenchymal stem cell differentiation at various matrix stiffnesses using a fibrous self-assembling peptide hydrogel. J. Biomed. Mater. Res. Part A.

[275] Wang Y.L., Lin S.P., Nelli S.R., Zhan F.K., Cheng H., Lai T.S., Yeh M.Y., Lin H.C., Hung S.C. (2017). Self-assembled peptide-based hydrogels as scaffolds for proliferation and multi-differentiation of mesenchymal stem cells. Macromol. Biosci..

[276] Luo H., Xu C., Liu Z., Yang L., Hong Y., Liu G., Zhong H., Cai X., Lin X., Chen X. (2019). Neural differentiation of bone marrow mesenchymal stem cells with human brain-derived neurotrophic factor gene-modified in functionalized self-assembling peptide hydrogel in vitro. J. Cell. Biochem..

[277] Kaur H., Roy S. (2021). Designing aromatic N-cadherin mimetic short-peptide-based bioactive scaffolds for controlling cellular behaviour. J. Mater. Chem. B.

[278] Caliskan O.S., Sardan Ekiz M., Tekinay A.B., Guler M.O. (2017). Spatial organization of functional groups on bioactive supramolecular glycopeptide nanofibers for differentiation of mesenchymal stem cells (MSCs) to brown adipogenesis. Bioconjug. Chem..

[279] Buchman C.A., Gifford R.H., Haynes D.S., Lenarz T., O’Donoghue G., Adunka O., Biever A., Briggs R.J., Carlson M.L., Dai P. (2020). Unilateral Cochlear Implants for Severe, Profound, or Moderate Sloping to Profound Bilateral Sensorineural Hearing Loss: A Systematic Review and Consensus Statements. JAMA Otolaryngol.–Head Neck Surg..

[280] Bergman J.E., Davies C., Denton A.J., Ashman P.E., Mittal R., Eshraghi A.A. (2021). Advancements in Stem Cell Technology and Organoids for the Restoration of Sensorineural Hearing Loss. J. Am. Acad. Audiol..

[281] Matsuoka A.J., Sayed Z.A., Stephanopoulos N., Berns E.J., Wadhwani A.R., Morrissey Z.D., Chadly D.M., Kobayashi S., Edelbrock A.N., Mashimo T. (2017). Creating a stem cell niche in the inner ear using self-assembling peptide amphiphiles. PLoS ONE.

[282] Sharma A., Sances S., Workman M.J., Svendsen C.N. (2020). Multi-lineage Human iPSC-Derived Platforms for Disease Modeling and Drug Discovery. Cell Stem Cell.

[283] Helmi S.A., Rohani L., Zaher A.R., El Hawary Y.M., Rancourt D.E. (2021). Enhanced Osteogenic Differentiation of Pluripotent Stem Cells via γ-Secretase Inhibition. Int. J. Mol. Sci..

[284] Hayashi K., Ochiai-Shino H., Shiga T., Onodera S., Saito A., Shibahara T., Azuma T. (2016). Transplantation of human-induced pluripotent stem cells carried by self-assembling peptide nanofiber hydrogel improves bone regeneration in rat calvarial bone defects. Bdj Open.

[285] Mabrouk M., Beherei H.H., Das D.B. (2020). Recent progress in the fabrication techniques of 3D scaffolds for tissue engineering. Mater. Sci. Eng. C.

[286] Francis N.L., Bennett N.K., Halikere A., Pang Z.P., Moghe P.V. (2016). Self-assembling peptide nanofiber scaffolds for 3-D reprogramming and transplantation of human pluripotent stem cell-derived neurons. ACS Biomater. Sci. Eng..

[287] Sirousazar M., Forough M., Farhadi K., Shaabani Y., Molaei R., Tiwari A. (2014). 9-Hydrogels: Properties, preparation, characterization and biomedical, applications in tissue engineering, drug, delivery and wound care. Advanced Healthcare Materials.

[288] Barros S.C., Martins J.A., Marcos J.C., Cavaco-Paulo A. (2012). Influence of secretory leukocyte protease inhibitor-based peptides on elastase activity and their incorporation in hyaluronic acid hydrogels for chronic wound therapy. Pept. Sci..

[289] Kruse C.R., Nuutila K., Lee C.C., Kiwanuka E., Singh M., Caterson E.J., Eriksson E., Sørensen J.A. (2015). The external microenvironment of healing skin wounds. Wound Repair Regen..

[290] Seow W.Y., Salgado G., Lane E.B., Hauser C.A. (2016). Transparent crosslinked ultrashort peptide hydrogel dressing with high shape-fidelity accelerates healing of full-thickness excision wounds. Sci. Rep..

[291] Guo S.a., DiPietro L.A. (2010). Factors affecting wound healing. J. Dent. Res..

[292] Loo Y., Wong Y.-C., Cai E.Z., Ang C.-H., Raju A., Lakshmanan A., Koh A.G., Zhou H.J., Lim T.-C., Moochhala S.M. (2014). Ultrashort peptide nanofibrous hydrogels for the acceleration of healing of burn wounds. Biomaterials.

[293] Moore A.N., Silva T.L.L., Carrejo N.C., Marmolejo C.A.O., Li I.-C., Hartgerink J.D. (2018). Nanofibrous peptide hydrogel elicits angiogenesis and neurogenesis without drugs, proteins, or cells. Biomaterials.

[294] Sun Y., Li W., Wu X., Zhang N., Zhang Y., Ouyang S., Song X., Fang X., Seeram R., Xue W. (2016). Functional self-assembling peptide nanofiber hydrogels designed for nerve degeneration. ACS Appl. Mater. Interfaces.

[295] Xie Z., Aphale N.V., Kadapure T.D., Wadajkar A.S., Orr S., Gyawali D., Qian G., Nguyen K.T., Yang J. (2015). Design of antimicrobial peptides conjugated biodegradable citric acid derived hydrogels for wound healing. J. Biomed. Mater. Res. Part A.

[296] Annabi N., Tamayol A., Uquillas J.A., Akbari M., Bertassoni L.E., Cha C., Camci-Unal G., Dokmeci M.R., Peppas N.A., Khademhosseini A. (2014). 25th anniversary article: Rational design and applications of hydrogels in regenerative medicine. Adv. Mater..

[297] Guan X., Avci-Adali M., Alarçin E., Cheng H., Kashaf S.S., Li Y., Chawla A., Jang H.L., Khademhosseini A. (2017). Development of hydrogels for regenerative engineering. Biotechnol. J..

[298] Zhao R., Li T., Zheng G., Jiang K., Fan L., Shao J. (2017). Simultaneous inhibition of growth and metastasis of hepatocellular carcinoma by co-delivery of ursolic acid and sorafenib using lactobionic acid modified and pH-sensitive chitosan-conjugated mesoporous silica nanocomplex. Biomaterials.

[299] Taylor D.L., in het Panhuis M. (2016). Self-Healing hydrogels. Adv. Mater.

[300] Loo Y., Goktas M., Tekinay A.B., Guler M.O., Hauser C.A., Mitraki A. (2015). Self-assembled proteins and peptides as scaffolds for tissue regeneration. Adv. Healthc. Mater..

[301] Del Valle L.J., Díaz A., Puiggalí J. (2017). Hydrogels for biomedical applications: Cellulose, chitosan, and protein/peptide derivatives. Gels.

[302] Seow W.Y., Hauser C.A. (2013). Tunable mechanical properties of ultrasmall peptide hydrogels by crosslinking and functionalization to achieve the 3D distribution of cells. Adv. Healthc. Mater..

[303] Kim S., Kim J.H., Lee J.S., Park C.B. (2015). Beta-sheet-forming, self-assembled peptide nanomaterials towards optical, energy, and healthcare applications. Small.

[304] Xiao Y., Reis L.A., Feric N., Knee E.J., Gu J., Cao S., Laschinger C., Londono C., Antolovich J., McGuigan A.P. (2016). Diabetic wound regeneration using peptide-modified hydrogels to target re-epithelialization. Proc. Natl. Acad. Sci. USA.

[305] Kim J.E., Lee J.H., Kim S.H., Jung Y. (2018). Skin regeneration with self-assembled peptide hydrogels conjugated with substance P in a diabetic rat model. Tissue Eng. Part A.

[306] Grek C.L., Prasad G., Viswanathan V., Armstrong D.G., Gourdie R.G., Ghatnekar G.S. (2015). Topical administration of a connexin43-based peptide augments healing of chronic neuropathic diabetic foot ulcers: A multicenter, randomized trial. Wound Repair Regen..

[307] Arslan E., Garip I.C., Gulseren G., Tekinay A.B., Guler M.O. (2014). Bioactive supramolecular peptide nanofibers for regenerative medicine. Adv. Healthc. Mater..

[308] Wickremasinghe N.C., Kumar V.A., Shi S., Hartgerink J.D. (2015). Controlled angiogenesis in peptide nanofiber composite hydrogels. ACS Biomater. Sci. Eng..

[309] Xu T., Tian Y., Zhang R., Yu B., Cong H., Shen Y. (2021). Hydrogel vectors based on peptide and peptide-like substances: For treating bacterial infections and promoting wound healing. Appl. Mater. Today.

[310] Marchesan S., Qu Y., Waddington L.J., Easton C.D., Glattauer V., Lithgow T.J., McLean K.M., Forsythe J.S., Hartley P.G. (2013). Self-assembly of ciprofloxacin and a tripeptide into an antimicrobial nanostructured hydrogel. Biomaterials.

[311] Paladini F., Meikle S., Cooper I., Lacey J., Perugini V., Santin M. (2013). Silver-doped self-assembling di-phenylalanine hydrogels as wound dressing biomaterials. J. Mater. Sci. Mater. Med..

[312] Irwansyah I., Li Y.Q., Shi W., Qi D., Leow W.R., Tang M.B., Li S., Chen X. (2015). Gram-positive antimicrobial activity of amino acid-based hydrogels. Adv. Mater..

[313] Laverty G., McCloskey A.P., Gilmore B.F., Jones D.S., Zhou J., Xu B. (2014). Ultrashort cationic naphthalene-derived self-assembled peptides as antimicrobial nanomaterials. Biomacromolecules.

[314] Doberdoli D., Bommer C., Begzati A., Haliti F., Heinzel-Gutenbrunner M., Juric H. (2020). Randomized Clinical Trial investigating Self-Assembling Peptide P11-4 for Treatment of Early Occlusal Caries. Sci. Rep..

[315] Gelain F., Luo Z., Rioult M., Zhang S. (2021). Self-assembling peptide scaffolds in the clinic. NPJ Regen. Med..

[316] Ding X., Zhao H., Li Y., Lee A.L., Li Z., Fu M., Li C., Yang Y.Y., Yuan P. (2020). Synthetic peptide hydrogels as 3D scaffolds for tissue engineering. Adv. Drug Deliv. Rev..

[317] Wen Y., Waltman A., Han H., Collier J.H. (2016). Switching the Immunogenicity of Peptide Assemblies Using Surface Properties. ACS Nano.

[318] Sis M.J., Webber M.J. (2019). Drug delivery with designed peptide assemblies. Trends Pharmacol. Sci..

[319] Sedlakova Kondelova P., Mannaa A., Bommer C., Abdelaziz M., Daeniker L., di Bella E., Krejci I. (2020). Efficacy of P(11)-4 for the treatment of initial buccal caries: A randomized clinical trial. Sci Rep.

[320] Bröseler F., Tietmann C., Bommer C., Drechsel T., Heinzel-Gutenbrunner M., Jepsen S. (2020). Randomised clinical trial investigating self-assembling peptide P(11)-4 in the treatment of early caries. Clin. Oral Investig..

[321] Gözetici B., Öztürk-Bozkurt F., Toz-Akalın T. (2019). Comparative Evaluation of Resin Infiltration and Remineralisation of Noncavitated Smooth Surface Caries Lesions: 6-month Results. Oral Health Prev. Dent..

[322] Welk A., Ratzmann A., Reich M., Krey K.F., Schwahn C. (2020). Effect of self-assembling peptide P(11)-4 on orthodontic treatment-induced carious lesions. Sci. Rep..

[323] Giritharan S., Salhiyyah K., Tsang G.M., Ohri S.K. (2018). Feasibility of a novel, synthetic, self-assembling peptide for suture-line haemostasis in cardiac surgery. J. Cardiothorac. Surg..

[324] Rahmani G., Prats J., Norchi T., Kates S., McInerney V., Woods J., Kelly J. (2018). First Safety and Performance Evaluation of T45K, a Self-Assembling Peptide Barrier Hemostatic Device, After Skin Lesion Excision. Dermatol. Surg..

[325] Subramaniam S., Kandiah K., Chedgy F., Fogg C., Thayalasekaran S., Alkandari A., Baker-Moffatt M., Dash J., Lyons-Amos M., Longcroft-Wheaton G. (2021). A novel self-assembling peptide for hemostasis during endoscopic submucosal dissection: A randomized controlled trial. Endoscopy.

[326] Kondo Y., Nagasaka T., Kobayashi S., Kobayashi N., Fujiwara T. (2014). Management of peritoneal effusion by sealing with a self-assembling nanofiber polypeptide following pelvic surgery. Hepato-Gastroenterology.

